# Identification of new quorum sensing autoinducer binding partners in *Pseudomonas aeruginosa* using photoaffinity probes†
†Electronic supplementary information (ESI) available. See DOI: 10.1039/c7sc01270e



**DOI:** 10.1039/c7sc01270e

**Published:** 2017-08-29

**Authors:** Y. R. Baker, J. T. Hodgkinson, B. I. Florea, E. Alza, W. R. J. D. Galloway, L. Grimm, S. M. Geddis, H. S. Overkleeft, M. Welch, D. R. Spring

**Affiliations:** a Department of Chemistry , University of Cambridge , Lensfield Road , Cambridge , CB2 1EW , UK . Email: spring@ch.cam.ac.uk; b Leiden Institute of Chemistry , Leiden University , Einsteinweg 55 , 2333 CC Leiden , The Netherlands; c Department of Biochemistry , University of Cambridge , 80 Tennis Court Road , Cambridge , CB2 1GA , UK . Email: mw240@cam.ac.uk

## Abstract

Many bacterial species, including the human pathogen *Pseudomonas aeruginosa*, employ a mechanism of intercellular communication known as quorum sensing (QS), which is mediated by signalling molecules termed autoinducers. The *Pseudomonas* Quinolone Signal (PQS) and 2-Heptyl-3*H*-4-Quinolone (HHQ) are autoinducers in *P. aeruginosa*, and they are considered important factors in the progress of infections by this clinically relevant organism. Herein, we report the development of HHQ and PQS photoaffinity-based probes for chemical proteomic studies. Application of these probes led to the identification of previously unsuspected putative HHQ and PQS binders, thereby providing new insights into QS at a proteomic level and revealing potential new small molecule targets for virulence attenuation strategies. Notably, we found evidence that PQS binds RhlR, the cognate receptor in the Rhl QS sub-system of *P. aeruginosa*. This is the first indication of interaction between the Rhl and PQS systems at the protein/ligand level, which suggests that RhlR should be considered a highly attractive target for antivirulence strategies.

## Abstract 2

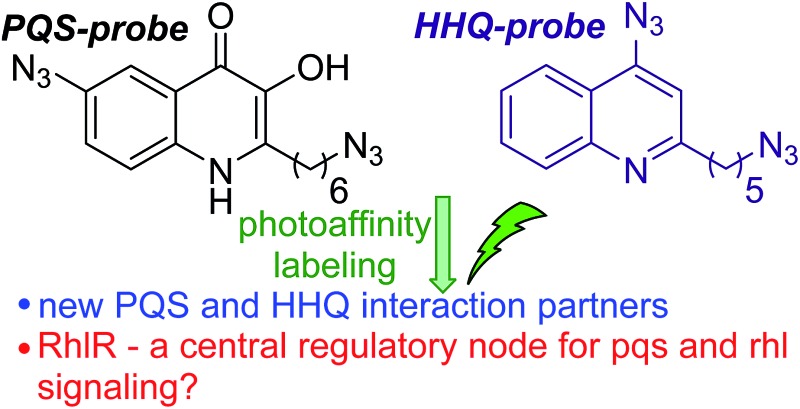
Design, synthesis and application of PQS and HHQ probes for investigating quinolone quorum sensing pathways using photoaffinity labeling.

## Introduction

*Pseudomonas aeruginosa* is a clinically relevant Gram-negative human pathogen that can cause acute or chronic infections, particularly in immunocompromised patients. It is responsible for many nosocomial infections, such as post-surgery infections, catheter associated infections, and ventilator associated pneumonia.^1–4^ *P. aeruginosa* is also a major cause of mortality in cystic fibrosis (CF) patients,^5,6^ and the incidence of multi-drug resistant strains is on the rise globally.^7^ The pathogenicity of this Gram-negative bacterium is attributed to its ability to produce and secrete a large arsenal of virulence factors which are inimical to the physiology of the infected host.^8^ Such virulence factors include: tissue degrading enzymes, endotoxins, exotoxins, siderophores, organic molecules, and adherence components.^9^

**Fig. 1:**
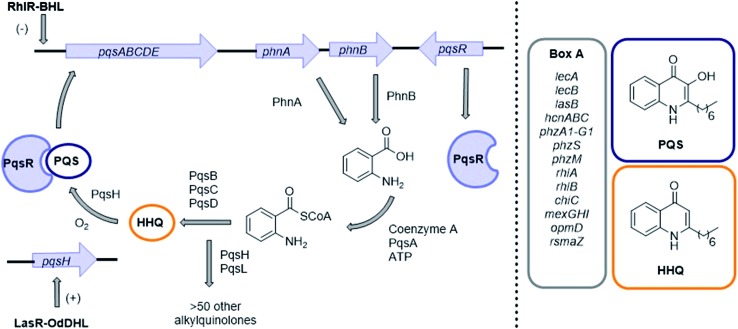
PQS signaling in *P. aeruginosa*. Genes involved in virulence production and regulation at a transcriptional level are highlighted in ‘Box A’ on the right. For a full list of genes under PqsR (also known as MvfR) transcriptional regulation see article by Déziel *et al.* ^25^ HHQ also binds PqsR albeit with a 100-fold lower potency than PQS.^20^ (–) Represents down-regulation (+) represents positive regulation.

A cell–cell signaling phenomenon known as quorum sensing (QS) enables bacteria to regulate gene transcription in a population cell density-dependent manner.^10–14^ QS is mediated by small-molecular weight signaling molecules that are synthesized continually throughout growth and released into the surrounding media. These signaling molecules diffuse between cells and once a critical threshold concentration has been reached, a specific receptor protein, which also acts as a transcriptional regulator, is activated, leading to a population-wide change in gene expression. The transcription of genes involved in signaling molecule biosynthesis are also up-regulated; hence, signaling molecules are often termed autoinducers.^10^ This natural phenomenon allows bacterial cells in populations to act collectively to coordinate gene expression. Different bacterial species utilize different classes of signaling molecules and receptor proteins, and a diverse array of phenotypes is now known to be under QS control.^15^ *P. aeruginosa* produces three QS signaling molecules: (*S*)-3-oxo-dodecanoyl-homoserine lactone (OdDHL), (*S*)-butyl-homoserine lactone (BHL) and 2-heptyl-3-hydroxy-4(1*H*)-quinolone, also known as the *Pseudomonas* Quinolone Signal (PQS).^16^ LasR is the principal receptor protein for OdDHL, and RhlR is the principal receptor for BHL.^17,18^ Another LuxR-type protein, QscR, is also known to bind OdDHL.^19^ PQS and its biosynthetic precursor, 2-Heptyl-3*H*-4-Quinolone (HHQ), bind the receptor protein PqsR, resulting in the up-regulation of many virulence factors including pyocyanin, LecA and elastase.^20–26^ We have also recently identified and characterized a binding interaction between PQS and the efflux pump component MexG.^27^ Independent of PqsR, PQS is associated with several other bacterial phenotypes including pyoverdine production, membrane vesicle formation and efflux pump regulation (Fig. 1).^28–33^

PQS and HHQ have been detected in high abundance in sputum isolates from cystic fibrosis (CF) patients with chronic *P. aeruginosa* infections,^34^ and *P. aeruginosa* has been shown to produce PQS maximally during stationary phase.^23,28^ Since production of these molecules continues long after the initial infection, this suggests that PQS and HHQ are also required for the persistence of *P. aeruginosa* in the airways.^34^

Despite increasing interest in the biological activities of PQS and HHQ, most studies to date have been performed on a genetic rather than proteomic level. However, and given the roles played by both PQS and HHQ in the global control of virulence, we sought to develop chemical probes for studying PQS and HHQ at a proteome level. Previously, we developed immobilized PQS and HHQ probes for affinity pull-down studies.^27^ While these probes successfully pulled-down several previously unreported protein interaction partners, the pull-downs suffered from high levels of background binding, and required pre-fractionation of the proteome. In addition, it was necessary to install a polyethylene glycol (PEG) linker moiety into these probes in order to achieve their immobilization; this moiety could potentially be detrimental to the biological activity of such probes when investigating novel or uncharacterized binding partners. To overcome these limitations, and taking inspiration from work by others in the field of target identification, we turned our attention towards photoaffinity labeling (PAL). PAL is a powerful technique that has previously been used for target identification and for defining possible off-target effects of drug molecules.^35–42^ We here report on the design, synthesis and application of PQS and HHQ probes for investigating quinolone quorum sensing pathways using photoaffinity labeling.

## Results

### Probe design considerations

**Fig. 2:**
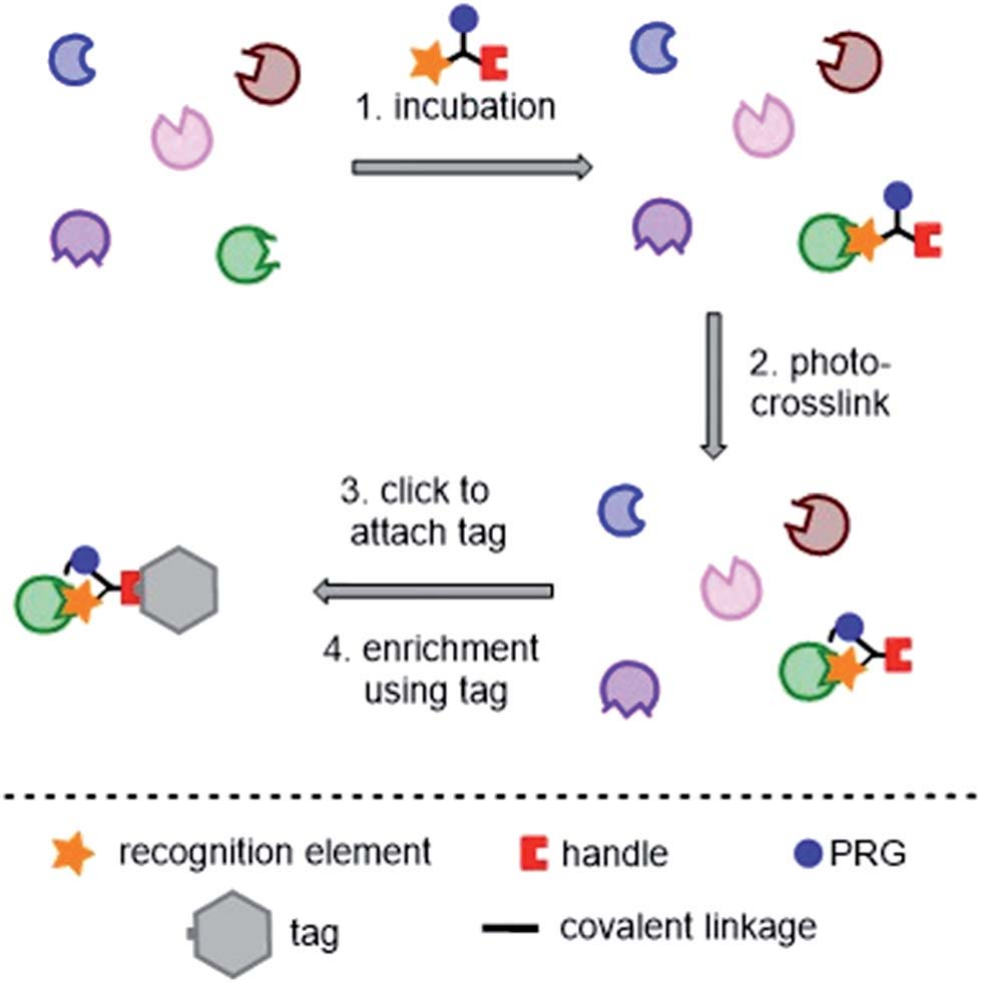
Typical tandem photoaffinity experiment for target identification. PRG = photoreactive group.

Photoaffinity probes for “two-step” or “tandem” PAL experiments contain three key moieties: a recognition element (typically the small molecule of interest), a photoreactive group (PRG), and a ligation handle for the attachment of a reporter tag post-labeling. A typical tandem photoaffinity experiment involves the following stages: (1) the probe is incubated with the proteome of interest (either whole cells or cell lysates); (2) UV light is used to activate the photoreactive group and a covalent bond is formed with nearby proteins (“photo-crosslinking”); (3) a bio-orthogonal reaction is used to attach the “tag” function to the probe *via* the ligation handle; (4) the tag is used to enrich and isolate the labelled proteins, enabling analysis (Fig. 2). Whilst such labeling strategies have greatly improved the success of photoaffinity target identification, non-specific hits and false positives still remain a big problem, and probe design is a major concern in proteomic studies.

PQS and HHQ differ only by the introduction of a hydroxyl group at the 3-position of the quinolone heterocycle. In addition, the interaction between PQS and PqsR is primarily driven by hydrophobic interactions,^43^ and *P. aeruginosa* produces over 50 other alkylquinolones.^44^ Thus, we envisaged that careful design would be necessary to “tune” the probes to investigate true PQS and HHQ protein binders, as well as to reduce non-specific hits. As the first step towards this, we set out to investigate the effect of commonly used ligation handles on the biological activity.

### Ligand handle evaluation

The ligation handles we considered were: (1) an alkyl azide, for use in the Staudinger ligation and copper(i)-catalyzed Huisgen [3 + 2]-azide alkyne cycloaddition (the reaction that became famous as the “copper-click reaction”);^40^ (2) an alkyne group, also for use in the copper-click reaction;^40^ (3) a ketone functional group, for use in the aniline catalyzed ketone-hydrazine condensation,^42^ and (4) the allyl group, for use in a photoactivated 1,3-dipolar cycloaddition reaction.^45^ We considered potential positions on HHQ and PQS where these handles could be installed without affecting biological activity. Several groups, including ours, have investigated the structure activity relationships (SAR) of the PQS signaling molecule.^32,33,43,46–48^ The optimum length of the alkyl chain is seven carbon atoms, and minor variations in the length of the alkyl chain are well tolerated.^32,33^ However, the AHQ binding pocket in PqsR is intolerant of bulky aromatic substituents and non-alkyl chains.^32^ PQS has also been demonstrated to tolerate certain substituents on the 5- or 6-positions of the quinolone ring.^32^ Based on these previous studies, and synthetic tractability, quinolone analogues **1–6** were prepared (Table 1, see ESI for synthesis†). These analogues incorporated modifications at the 5- and 6-positions of the quinolone aromatic region, and the terminal end of the alkyl chain. The biological activity of each analogue was evaluated and compared with the native HHQ and PQS ligands (Table 1). This was achieved by quantifying the ability of each analogue to activate the PqsR receptor using an *Escherichia coli*-based reporter strain containing a plasmid encoding PqsR and a PqsA:LacZ fusion.^49^ The reporter strain was grown in the presence of each of the compounds at a concentration of 60 nM for the PQS analogues, and 1 μM for the HHQ analogues. As PQS has been associated with several bacterial phenotypes independent of PqsR we also wanted to evaluate the analogues in a PqsR independent assay.^28–33^ To achieve this we then evaluated the analogues in a *P. aeruginosa* based assay, utilizing the fact that PQS has been shown to affect the production of the siderophore pyoverdine, independent of PqsR.^32^ Pleasingly, PQS analogue **1** with the azide at the terminal end of the alkyl chain maintained strong agonistic activity in both assays. The use of analogue **2**, with an allyl functionality, resulted in near complete loss of ability to stimulate PqsR-dependent PqsA transcription; a surprising result as we had previously found that activity was enhanced by an octyl carbon chain with a terminal alkene.^32^ Comparing HHQ analogues **3** and **4**, with the alkyne in the 6-position of the aromatic ring or the terminal position of the alkyl chain respectively, biological activity was partially retained, albeit not as much as in the native HHQ ligand. The introduction of a ketone group in analogues **5** and **6** resulted in a substantial reduction in PqsR-dependent agonist activity. Given the strong agonist activity of **1** in comparison with PQS, we envisaged that incorporation of an azide functionality at the terminal end of the heptyl chain as a ligation handle in the final HHQ probe would be a viable option as well.

**Table 1:** Ability of analogues to stimulate transcription from the PqsA promoter and promote pyoverdine production relative to PQS (for **1** and **2**, PQS = 100% stimulation) and HHQ (for **3–6**, HHQ = 100% stimulation)^*a*^

		% Stimulation	
		psqA:LacZ promoter	Pyoverdine production
**1**	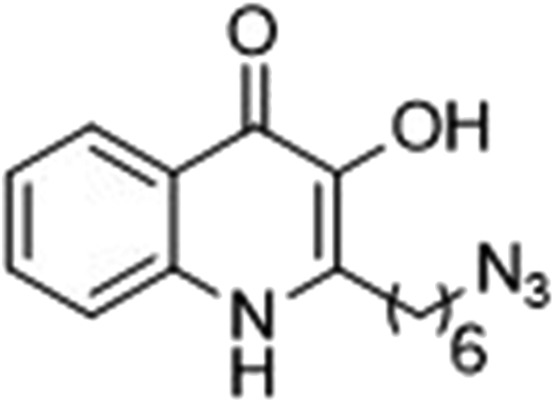	103	132
**2**	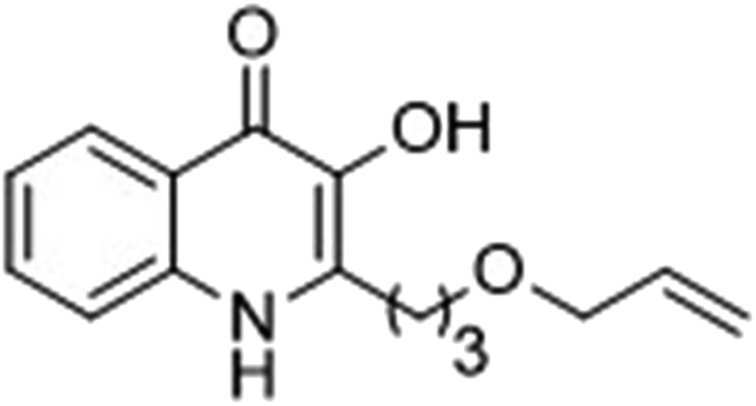	13	29
**3**	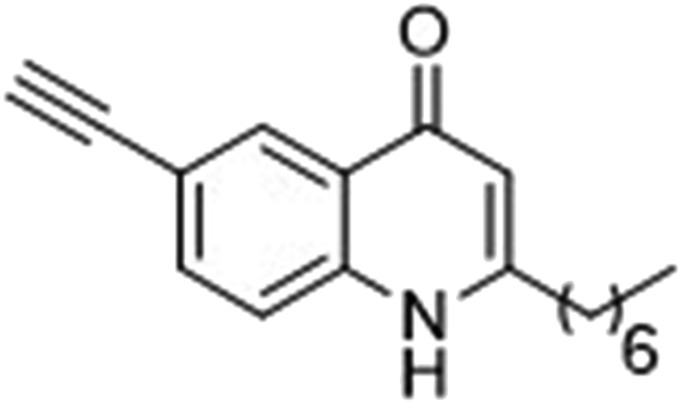	27	61
**4**	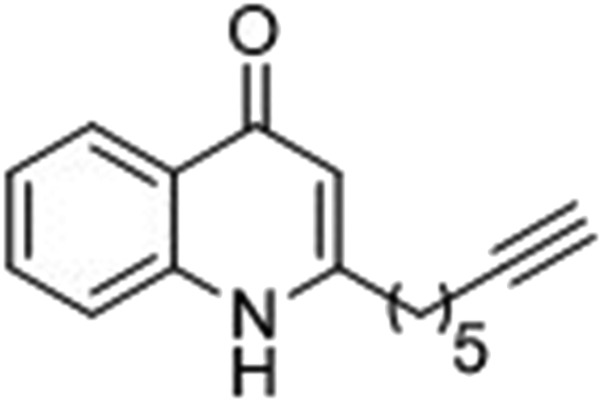	40	69
**5**	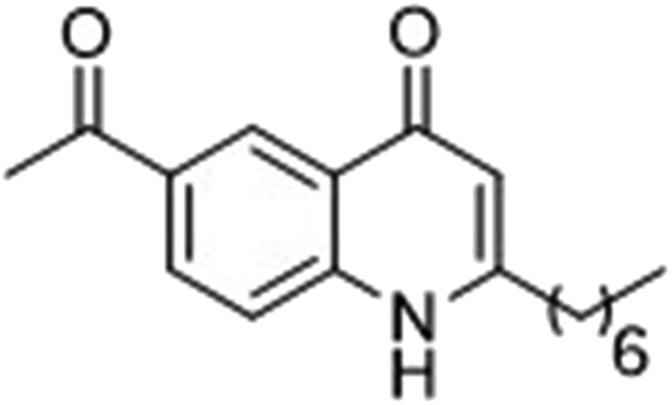	–8	47
**6**	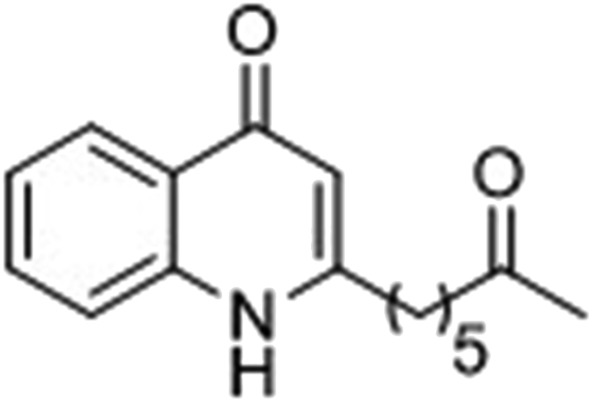	22	39

^*a*^All assays performed in triplicate; error did not exceed ±10% of the mean.

With the azide functionality on the terminal end of the chain selected as the linker handle for the HHQ and PQS probes, we next sought to investigate potential photoreactive groups.

### Photoreactive group evaluation

As the linker handle was to be located on the alkyl chain, we envisaged positioning the photoreactive group on the aromatic region of HHQ and PQS. This would allow the alkyl chain to function as an endogenous pseudo linker, avoiding the need for a linker moiety entirely, thereby minimizing further structural modifications to the PQS and HHQ core structures. The three most commonly used photoreactive groups are diazirines,^41^ aryl azides,^50^ and benzophenones.^38^ Benzophenones were excluded based on their size. It was anticipated that the installation of a diazirine in this position would pose a significant synthetic challenge. However, aryl azides are well known to be comparatively easy to prepare. As such, the aryl azide functionality was chosen for further investigation. To confirm that biological activity would be retained, PQS and HHQ analogues with an azido group in the 6-position (**7** and **8** respectively, Table 2, see ESI for synthesis†), were synthesized and their biological activity evaluated as before. Introduction of the azido group resulted in minor losses of activity in both assays compared with the native ligands, but we speculated that such minor losses in activity would not be detrimental to the final probes. It was initially anticipated that quinoline **9** would show diminished biological activity and could potentially be used as a negative control (Table 2, see ESI for synthesis†). However, and to our surprise, the biological activity was found to be comparable with HHQ. These findings provide the first indications that the carbonyl functionality of the quinolone of HHQ and PQS can be modified to an extent without the loss of biological activity. Based on these SAR data, we proceeded to synthesize the final probes for proteomic studies.

**Table 2:** Ability of analogues to stimulate transcription from the PqsA promoter and to promote pyoverdine production, relative to PQS (for **7**, PQS = 100% stimulation) and HHQ (for **8** and **9**, HHQ = 100% stimulation)^*a*^

		% Stimulation	
		psqA:LacZ promoter	Pyoverdine production
**7**	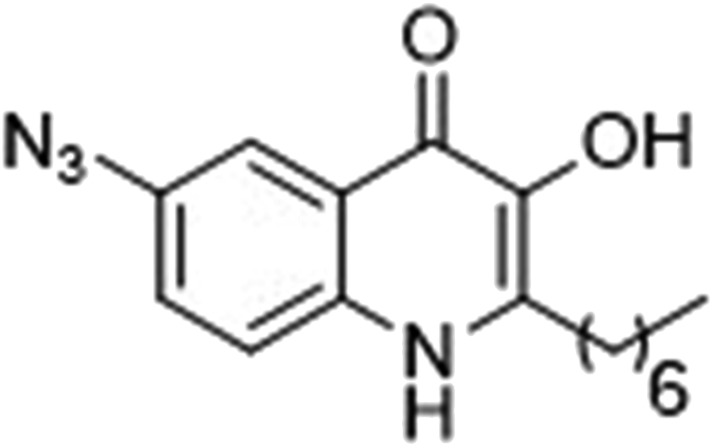	84	75
**8**	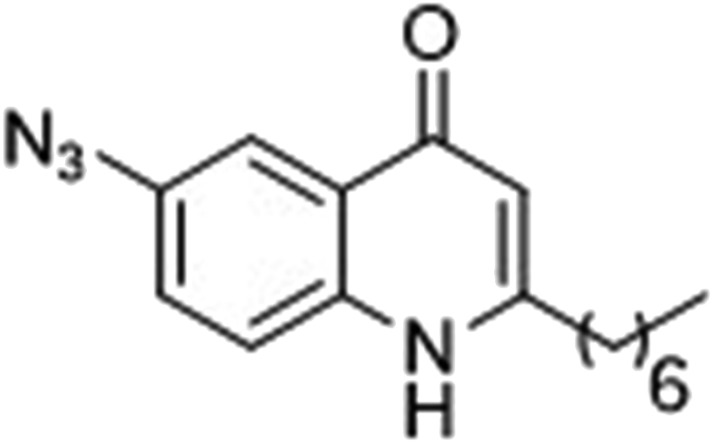	88	54
**9**	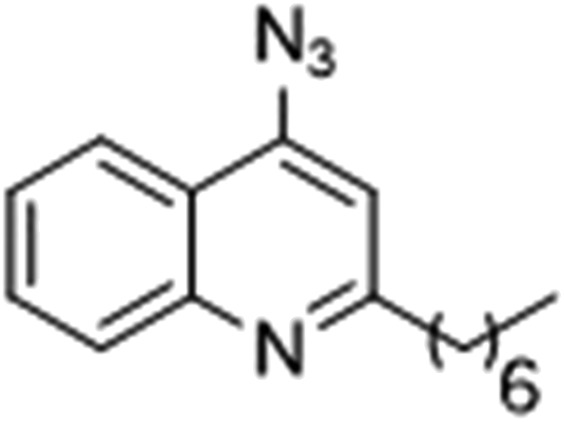	94	128

^*a*^All assays performed in triplicate; error did not exceed ±10% of the mean.

### Design of PQS, HHQ and control probes

**Fig. 3:**
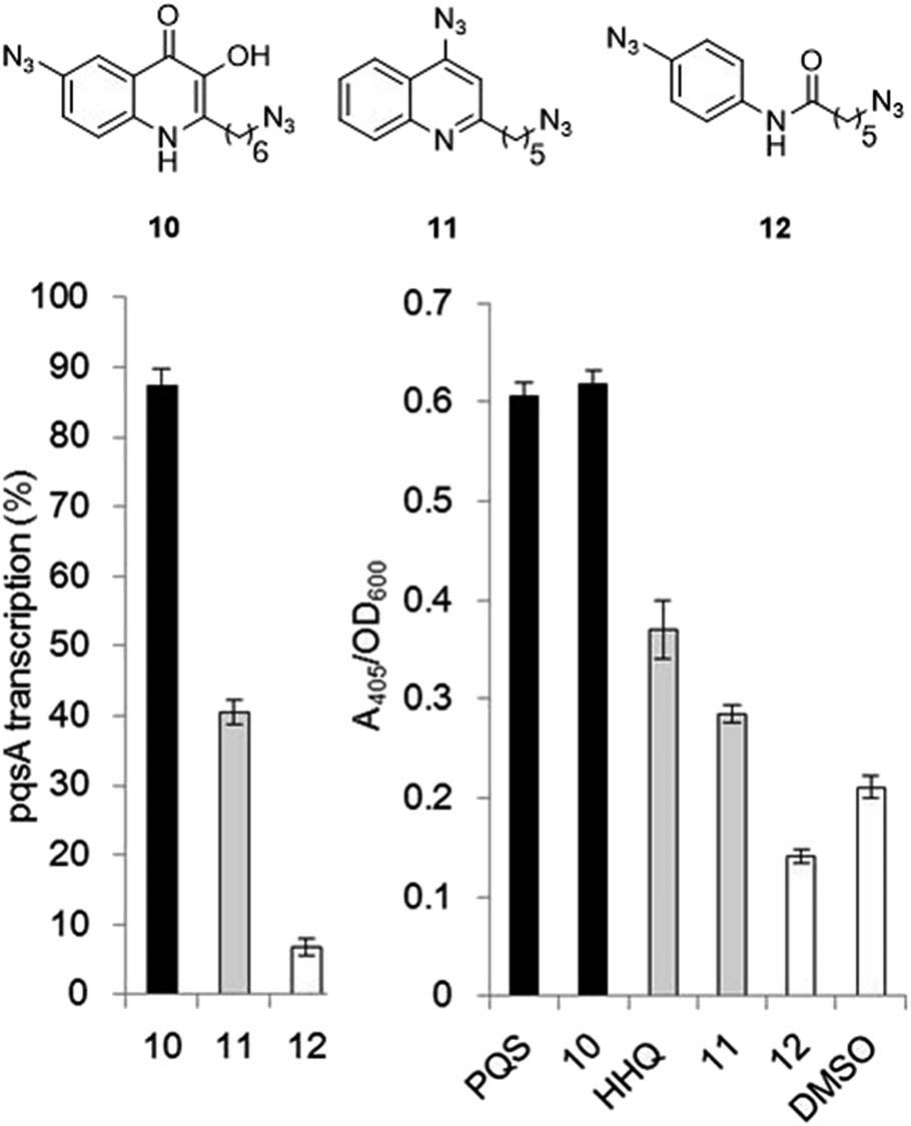
Comparison of the bioactivity associated with the PQS probe **10**, the HHQ probe **11**, and the negative control **12** with native PQS and HHQ in stimulating PqsR-dependent transcription from the PqsA promoter (left panel) and in promoting PqsR-independent production of pyoverdine (right panel). Percentage PqsA:LacZ transcription was compared relative to 60 nM PQS for the PQS probe **10** (black bar), and to 1 μM HHQ for the HHQ probe **11** (grey bar) and negative control probe **12**. ± Represents standard deviation of three independent biological replicates.

The final PQS probe **10**, HHQ probe **11**, and the negative control probe **12** were synthesized (see ESI†) and the activities of these probes were compared with PQS and HHQ in both bioassays as before (Fig. 3). Due to synthetic tractability, a pentyl chain was incorporated in the HHQ probe **11**.

Remarkably, the PQS probe **10** retained almost full activity compared with PQS in both the pyoverdine assay, and in the PqsR-dependent PqsA:LacZ transcription assay. However, and although the HHQ probe **11** retained a good ability to promote pyoverdine production compared to HHQ, it also showed diminished ability to stimulate PqsR-dependent transcription from the PqsA promoter. This notwithstanding, probe **11** was still far more bioactive than the negative control, and we believed the probe to have valid use in target identification.

### Evaluation of the probes in *E. coli*

Before proceeding to studies in *P. aeruginosa*, we wanted to confirm that the probes were able to label the ligand-binding domain of PqsR. The PqsR_LBD_–MBP (Ligand-Binding Domain–Maltose Binding Protein fusion protein) was over-expressed in *E. coli* as previously reported,^51^ and cell lysates containing the protein were prepared by sonication. PqsR_LBD_–MBP was chosen as we had previously found that the full length overexpressed PqsR was insoluble. The lysates were incubated with each of the probes, then irradiated with UV light. Following this, the copper catalyzed azide alkyne cycloaddition (CuAAC) was used to attach a tetramethyl-6-carboxyrhodamine (TAMRA) alkyne tag to the azide moiety of each probe. The samples were then resolved by SDS-PAGE, and the labelled proteins were visualized by fluorescence imaging and Coomassie Brilliant Blue staining. A strongly-labeled single band of around ∼70 kDa (corresponding to the PqsR_LBD_–MBP) was observed. Subsequent analyses revealed that the optimum labeling conditions were obtained using a final probe concentration of 1 μM and an irradiation time of 5 min using UV light with a wavelength of either 302 nM or 365 nm. No labeling of the PqsR_LBD_ was observed with the negative probe even when the probe was present at 1 mM concentration (ESI Fig. S1†).

**Fig. 4:**
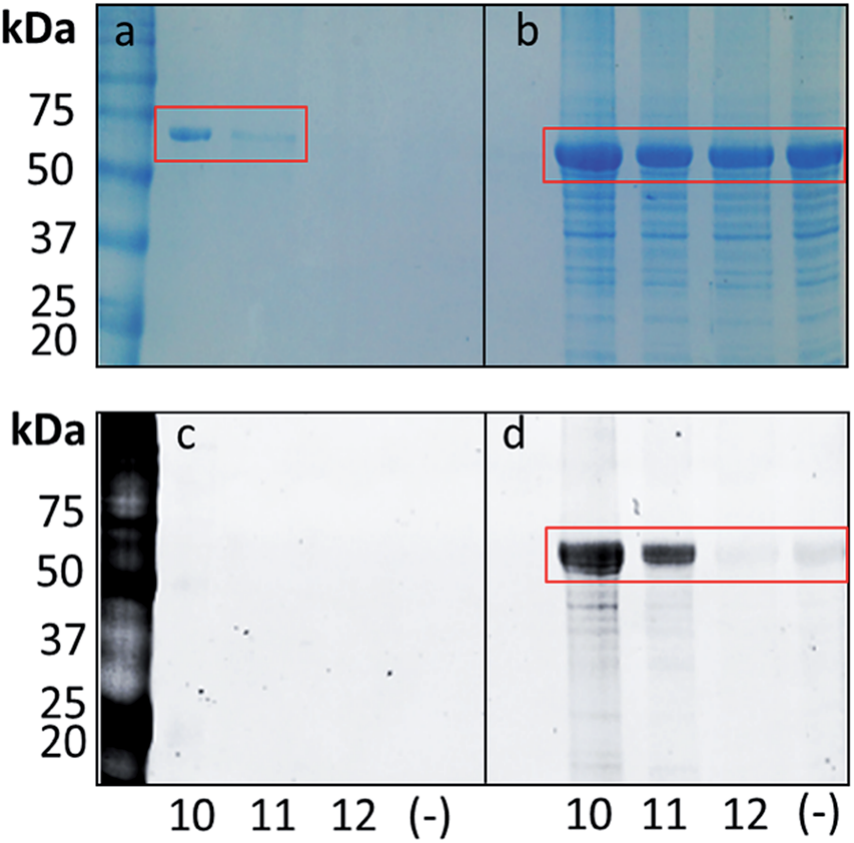
Selective labelling of over-expressed PqsR_LBD_ in intact *E. coli* cells by the PQS probe (**10**), HHQ probe (**11**) and negative control (**12**) (left panels). Bead-captured PqsR_LBD_ obtained after UV irradiation of probe-treated cultures, cell lysis, CuAAC reaction with a biotin moiety, and pull-down with streptavidin coated magnetic beads. The panels show an SDS-PAGE gel visualized after (a) Coomassie Brilliant Blue staining or (c) fluorescence imaging (right panels). The panels show images of an SDS-PAGE gel of cell extracts obtained after UV irradiation of probe-treated cultures, cell lysis, and CoAAC-mediated TAMRA labeling. The gels were visualized using (b) Coomassie Brilliant Blue staining and (d) fluorescence imaging. Molecular mass markers are shown. (–) = no probe added. The ∼70 kDa PqsR_LBD_–MBP band is highlighted by boxing.

Next, we set out to determine if the probes could successfully label and pull-down the PqsR_LBD_–MBP in live cells. The probes were added to early stationary phase cultures and the bacteria were grown for a further 30 min. The cells were then irradiated with UV light (*λ* = 302 nM) to initiate photo-crosslinking, before lysis and resolution of the labelled proteins by SDS-PAGE. The CuAAC with the TAMRA functionalized alkyne was performed by the same method as used in the cell lysate protocol. Comparing Coomassie Brilliant Blue staining of the polyacrylamide gel with visualization by fluorescence imaging (Fig. 4a) we observed very selective labeling in the presence of the PQS and HHQ probes. Adding the probe 30 min prior to UV irradiation gave the highest level of labeling and no significant difference was observed with longer incubation times. Labeling was further improved by increasing the probe concentration to 100 μM and the irradiation time to 15 min (ESI, Fig. S2†). We also demonstrated that it is possible to label PqsR with a biotin moiety. To achieve this, after UV irradiation of the cell culture and cell lysis, a CuAAC reaction with an alkyne functionalized biotin was performed. The samples were then incubated with streptavidin coated magnetic beads, stringently washed to remove non-specific binders, and the biotin labelled proteins eluted by denaturation in SDS loading buffer. The samples were analyzed by SDS-PAGE and Coomassie Brilliant Blue staining (Fig. 4b). PQS probe **10** yielded a strong band at the anticipated molecular mass (*ca.* 70 kDa). Pull-downs with HHQ probe **11** also yielded the PqsR_LBD_–chimera, albeit in lower amounts than the PQS probe. This is perhaps to be expected, given the lower binding affinity of HHQ to PqsR. No labeling was observed with negative control probe **12**. Thus, we confirmed the ability of the probes to label the PqsR_LBD_ in an *E. coli* background both in cell lysates and in live cells.

### Target identification of probes in *P. aeruginosa*

Having established the specificity and labeling efficiency of the probes, we next decided to use them to identify potentially novel 2-alkyl-4-quinolone (AHQ) binding partners in *P. aeruginosa* PAO1 cell lysates. Cells were harvested at early stationary phase (see ESI Fig. S3†) at which point PQS levels have been reported to plateau and PQS-mediated QS has been initiated.^23^ We hypothesized that this would be the optimal time point when protein binders of HHQ and PQS would be present. The cell lysates were incubated with, either the PQS probe **10**, the HHQ probe **11**, the negative probe **12**, or no probe, and irradiated with UV light (*λ* = 302 nM) for 10 min using the conditions identified for the *E. coli* cell lysate experiments. The photo-labelled protein samples were then subjected to CuAAC with biotin alkyne, followed by a pull-down with magnetic beads and stringent washing to remove non-specifically bound proteins. The captured proteins were identified by on-bead digest and subsequent LC-MS/MS analysis (see ESI, Tables S2–S7†). Proteins identified with the negative control probe and no-probe control experiments were eliminated from further analysis. The remaining proteins, which bound specifically to either the PQS or HHQ probes, are shown in Table 3. Given that we identified several proteins whose expression is known to be dependent on QS this way, we next repeated the experiments using a PqsR mutant and a RhlR mutant (Table 3). The PqsR mutant is deficient in alkyl quinolones, including PQS and HHQ, and is also deficient in quinolone-mediated QS. Similarly, the RhlR mutant is deficient in BHL-mediated QS. We speculated that the proteins identified in the wild type *P. aeruginosa*, but not in the QS deficient mutants, would provide further evidence that these are physiologically-relevant hits and are potential *in vivo* binders of PQS and HHQ (as well as supporting the theory that they are under QS regulation). In addition, we also analyzed the proteins pulled down by the probes at an earlier harvesting point in the growth curve (late exponential phase); see ESI, Section 1.3.†

**Table 3:** Proteins labeled in *P. aeruginosa* with PQS and HHQ probes^*a*^

Protein	PQS probe (**10**)			HHQ probe (**11**)			Protein function
	WT	ΔPqsR	ΔRhlR	WT	ΔPqsR	ΔRhlR	
WbpB	253	359	56	—	22	32	UDP-d-GlcNAcA oxidase
PA2069	88	—	—	—	—	—	Probable carbamoyl transferase
FtsZ	90	94	34	25	25	48	Cell division (tubulin homolog)
HcnC	85	—	—	—	—	—	Hydrogen cyanide synthase
PhzD1	83	—	—	—	—	—	Phenazine biosynthesis
PhzF1	78	—	—	—	—	—	Phenazine biosynthesis
Pfpl	69	—	—	—	—	—	Intracellular protease
PqsD	37	—	—	—	—	—	Alkyl quinolone biosynthesis
AstB	37	—	—	48	—	—	*N*-Succinylarginine dihydrolase
RhlR	26	100	—	—	—	—	Quorum sensing regulator

^*a*^Numerical values represent MASCOT scores for proteins identified, WT = Wild Type, — protein not identified in particular strain. The PqsR mutant was MP551 (PqsR:ISphoA/hah-Tc). The RhlR mutant was PDO111 (RhlR:Tn501).

## Discussion

In this work, we developed two probes (**10** and **11**) to identify potentially novel AHQ binders. Surprisingly, and although the PQS and HHQ probes were found to bind to PqsR when the latter was expressed in *E. coli*, we did not identify this protein as an interaction target in *P. aeruginosa*. We do not know why this is, although it seems likely that the binding pocket on PqsR is somehow occluded in the native background. These possibilities are discussed further below. However, our initial disappointment when PqsR was not identified was overcome by the identification of RhlR and PqsD as potential PQS interacting partners in wild type *P. aeruginosa*. As expected, PqsD was not pulled down in the PqsR mutant. PqsD is an enzyme with structural similarity to β-ketoacyl-ACP synthases such as FabH, and is required for the biosynthesis of HHQ and subsequently PQS.^52^ The X-ray crystal structure of PqsD reveals a covalent bond formed between the Cys112 amino acid of PqsD and anthranilate.^53^ The ligated PqsD is hypothesized to be involved in a decarboxylative coupling with malonyl-CoA to generate a reactive intermediate that undergoes further enzymatic steps to yield HHQ. Interestingly, it has been demonstrated that in the presence of PqsD, 3-ketodecanoic acid and anthraniloyl-CoA can form HHQ *in vitro*.^54^ However, 3-keto fatty acids have been ruled out as the direct biological precursors of quinolones *in vivo*.^55^ Further to this, a number of small molecules have been reported to bind and inhibit PqsD, indicating that small molecules can bind to PqsD and potentially regulate its activity (presumably through competitive or even allosteric interactions).^56^ The absence of the native full PqsR may be due to several reasons: the protein may not be sufficiently abundant, it may be tightly associated with DNA, or it may simply be too unstable under the experimental conditions used for enrichment. Alternatively, this could be a result of solubility issues, as the PqsR_LBD_–MBP expressed in the *E. coli* model would be considerably more soluble.^51^ We also noted the absence of MexG, which has been previously identified as a PQS binding partner in our studies with immobilized PQS. This is most likely due to variation between the two studies, including variation in the probes used.^27^ For example, MexG is a small integral membrane protein (approx. 15 kDa) and not highly abundant in cell lysate. In the absence of added detergent, MexG would not be solubilized (and therefore, would not bind efficiently with the streptavidin beads).

Of the eleven proteins identified with the PQS probe, six were present in the wild type (PAO1), but absent in both the RhlR and PqsR mutants. Thus, these six proteins are most likely under QS control and are potential binders of PQS or HHQ. Aside from AstB and PA2069, these proteins are involved in *P. aeruginosa* virulence. PhzD1 and PhzF1 are involved in the biosynthesis of the phenazines, which are well-known virulence factors.^57^ Phenazines are fluorescent, redox active molecules that damage epithelial cells and are toxic to competing organisms in the airways of cystic fibrosis patients.^58^ It has long been known that PQS and the phenazines are linked; in fact, the importance of PQS was first identified in studies aimed at identifying the genes important in phenazine production.^24^ Both the Pqs and Rhl signaling systems have been reported to regulate phenazine production.^23,59–61^ HcnC is a hydrogen cyanide synthase subunit; HCN is a potent virulence factor produced by *P. aeruginosa*.^62^ The cyanide anion is a non-competitive inhibitor of cytochrome c oxidase, affecting electron transport and in turn cellular respiration. Similar to pyocyanin, HCN biosynthesis is known to be under QS control, particularly by the Rhl system.^63,64^ PfpI is an intracellular protease, and ΔPfpI mutants are affected in swarming motility and biofilm formation.^65^ Intriguingly, pfpI transcription is downregulated in a PqsR mutant.^24^ Thus linking this protease to quinolone signaling at a transcriptional and proteomic level, making this protein a very interesting target for further studies. We speculate that FtsZ and WbpB are potential “off-target” binding partners of the quinolones, but could still have physiological relevance. For example, alkyl quinolones have been known to affect colony morphology,^66^ and mutations in FtsZ have also been linked to alteration in morphology phenotypes.^67^

Finally, perhaps our most intriguing and unexpected finding is that RhlR is a potential PQS binder. As expected, RhlR was not pulled down in the RhlR mutant, but was pulled down from extracts of the wild type and the PqsR mutant. As previously noted, RhlR is one of the three main QS receptors in *P. aeruginosa* and acts as a receptor protein for BHL. We were initially surprised considering that RhlR is also a transcriptional regulator, and is therefore probably not present in high abundance, similar to PqsR. Thus, the identification of RhlR in this study reveals a new insight into QS in *P. aeruginosa* which we believe is of significant importance. Numerous studies have previously linked the Rhl and Pqs signaling systems and both these systems have been found to be involved in the regulation of phenazine production.^23,24,59–61^ For example, Brouwer *et al.* have identified a RhlR binding-box nearly 400 nucleotides upstream of the PqsA translational start site.^61^ RhlR binding to this region results in an mRNA transcript that is able to form a stable hairpin secondary structure. Consequently, this mRNA product does not result in the translation of the PQS operon, unlike the PqsR induced mRNA transcript, which is shorter in length. In a separate study, Welsh and co-workers reported that BHL acts as an agonist of RhlR and results in repression of Pqs signaling and a reduction of pyocyanin production.^59^ The Pqs system also positively regulates the Rhl system, through an unknown mechanism.^26^ Excitingly, our findings reveal the possibility that PQS is also a binding partner of RhlR, or, alternatively, that RhlR forms a complex with a protein bound to PQS, as the possibility of a protein–protein interaction cannot be ruled out.

In order to validate the robustness of our pull-down protocol we sought to demonstrate the direct interaction of a representative protein labelled in this study with its corresponding AHQ. PqsD, which was identified as a putative binder of PQS, was selected. Thus, the PqsD protein was recombinantly overexpressed and subsequently purified (see ESI, Appendix 2†). Intrinsic tryptophan fluorescence spectroscopy was employed to measure whether PqsD and PQS interacted *in vitro*. The fluorescence spectra of purified PqsD with the addition of DMSO as a control indicate that there is no quenching of PqsD fluorescence spectra by DMSO (the traces overlapped, see ESI, Appendix 2, Fig. 1A†). However, a small but highly reproducible quenching of PqsD fluorescence is observed upon the addition of PQS (see ESI, Appendix 2, Fig. 1B†), indicating a change in the microenvironment of one or more tryptophan residues in PqsD. These data are consistent with the notion that PQS binds to purified PqsD, one of the proteins identified by the PQS probe as a novel binding partner. This provides proof-of-concept that our photoaffinity-based approach can be used to successfully identify binders of an AHQ and thus serves to validate the results obtained in this study.

## Conclusions

We have reported the development of PQS and HHQ photoaffinity probes capable of labeling the ligand-binding domain of PqsR *in vivo* and *in vitro*, enabling the facile capture and pull-down of this protein. These probes avoided the use of large modifications, did not require linker functionality, and have the potential to be used in live cells to study the biological binding partners of PQS and HHQ. Such studies are not necessarily restricted to *P. aeruginosa*, since they could also be used to analyze inter-species and even inter-kingdom signaling. Subsequent use of the probes in large-scale affinity purification led to the identification of several promising and previously unknown putative PQS binders, which will are the subject of detailed ongoing investigations. Our results indicate that PQS may bind a phenazine biosynthesis protein and also a regulatory protein, RhlR. Indeed, if RhlR does act as a central regulatory node in QS, capable of integrating signals from both the Rhl and Pqs signaling pathways, this would make RhlR a highly attractive target for anti-virulence strategies. In summary, the results of this proteomic study provide tantalizing new evidence that PQS-dependent signaling, BHL-dependent signaling and phenazine production are inter-linked. Our data also highlight the crucial importance of RhlR in QS regulation, not just at a transcriptional level but also at a proteomic level.

## Conflicts of interest

There are no conflicts to declare.
