# Supplementary material for: Identification of new quorum sensing autoinducer binding partners in Pseudomonas aeruginosa using photoaffinity probes
Source: Chem Sci. 2017 Aug 29;8(11):7403–11. doi: 10.1039/c7sc01270e (PMC5674140; doi:10.1039/c7sc01270e)
Supplement: Supplementary file 1 [file SC-008-C7SC01270E-s001.pdf]

Electronic Supplementary Material (ESI) for Chemical Science.
This journal is © The Royal Society of Chemistry 2017

Electronic Supplementary Information

Identification of new quorum sensing autoinducer binding partners in Pseudomonas aeruginosa using photoaffinity probes

Ysobel R. Baker, [a,b,c,†] James T. Hodgkinson,[a,c,‡] Bogdan I. Florea,[b] Esther Alza,[a,¶] Warren R. J. D. Galloway,[a] Larson Grimm[c], Stephen M. Geddis,[a] Herman S. Overkleeft,[b] Martin Welch,[*,c] and David R. Spring[*,a]

[a] Department of Chemistry, University of Cambridge, Lensfield Road, Cambridge, CB2 1EW, United Kingdom

[b] Leiden Institute of Chemistry, Leiden University, Einsteinweg 55, 2333CC Leiden, The Netherlands

[c] Department of Biochemistry, University of Cambridge, 80 Tennis Court Road, Cambridge, CB2 1GA

[†] Present address Y.R.B.: *Department of Chemistry, Physical and Theoretical Chemistry Laboratory, South Parks Road, Oxford OX1 3QZ, U.K.*
[‡] Present address J.T.H.: *Department of Chemistry, University of Leicester, Leicester LE1 7RH, U. K.*
[¶] Present address E.A.: *Institute of Chemical Research of Catalonia (ICIQ), Barcelona Institute of Science and Technology, Av. Països Catalans 16, 43007, Tarragona, Spain*

Corresponding Authors

* spring@ch.cam.ac.uk

* mw240@cam.ac.uk

Table of Contents

1 Supplementary discussion 3

1.1 Evaluation of the probes photoreactivity 3

1.2 Growth curves and harvesting of the bacterial strains used in this study 6

1.3 SDS PAGE analysis of pull-downs 7

2 Supplementary data 9

2.1 Biological data 9

3 Supplementary tables 12

3.1 Peptide tables for proteins in text and supplementary discussion 12

4 Supplementary methods 16

4.1 Biological experimental section 16

4.2 Pull-down experiments for protein identification 19

4.3 Synthetic experimental 21

4.3.1 Synthesis of PQS analogue 1 22

4.3.2 Synthesis of PQS analogue 2 24

4.3.3 Synthesis of HHQ analogue 3 26

4.3.4 Synthesis of HHQ analogue 4 28

4.3.5 Synthesis of HHQ analogue 5 30

4.3.6 Synthesis of HHQ analogue 6 31

4.3.7 Synthesis of PQS analogue 7 32

4.3.8 Synthesis of HHQ analogue 8 34

4.3.9 Synthesis of HHQ analogue 9 35

4.3.10 Synthesis of PQS probe 10 36

4.3.11 Synthesis of HHQ probe 11 39

4.3.12 Synthesis of negative control probe 12 40

4.4 NMR spectra 42

5 Supplementary Appendix 1 - UV traces for probes 10-12 77

6 Supplementary Appendix 2 - PqsD cloning and purification and fluorimetry protocol 79

7 References 81

1 Supplementary discussion

1.1 Evaluation of the probes photoreactivity

Characterisation of probes. The UV absorption spectra for each of the probes were recorded in the range of 200-550 nm under neutral conditions. Whilst all probes had a maximum absorption below 300 nm they all had a second peak in the range of λ= 300-340 nm except for probe 14 (Supplementary Table 1). The UV traces for the probes are given in Supplementary Appendix 1.

Probe		λ_{max} (nm)
**10**	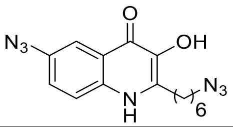	265<br>307<br>336
**11**	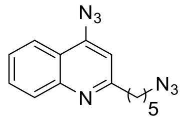	241<br>271<br>315
**12**	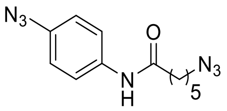	267

Supplementary Table 1. Quinolone probes and results of photo-reactivity studies using UV light.

Optimisation of labelling conditions. The labelling protocol was investigated as follows. The ligand binding domain of PqsR was overexpressed as a maltose binding protein fusion ($PqsR_{LBD}$-MBP) using an *E. coli* strain with the plasmid pMAL-c2X containing a *pqsR* ligand binding domain (LBD) insert.[1] The cells were harvested, lysed in PBS containing a protease inhibitor cocktail tablet (Roche) by sonication and clarified by centrifugation. The cell pellet was discarded and the supernatant used as the cell-lysate samples. The Bradford Assay was then used to determine the protein concentration and the sample was diluted with PBS containing a protease inhibitor cocktail tablet (Roche) to a final concentration of 1 mg/mL.

PQS probe **10** and HHQ probe **11** were added to aliquots of the cell-lysate to a final concentration of 1 μM in 1% DMSO. The samples were then irradiated using the inverted transilluminator setup keeping the samples at 4 °C. Samples were irradiated with either 302 nm or 365 nm UV light for either 5, 10, 15 or 30 min. Click chemistry was used to add a TAMRA (6-carboxytetramethylrhodamine) dye to the labelled proteins before the proteins were precipitated using TCA. The samples were resolved by SDS PAGE and scanned for

fluorescence prior to Coomassie staining. As a control, Probe **12** was also added to a final concentration of 1 µM in 1% DMSO and irradiated for 30 min using 302 nM. Both the PQS probe **10** and the HHQ probe **11** successfully labelled the overexpressed protein while probe **12** did not, confirming that the probes were capable of labelling the $PqsR_{LBD}$-MBP in cell-lysate. (Supplementary Fig. 1).

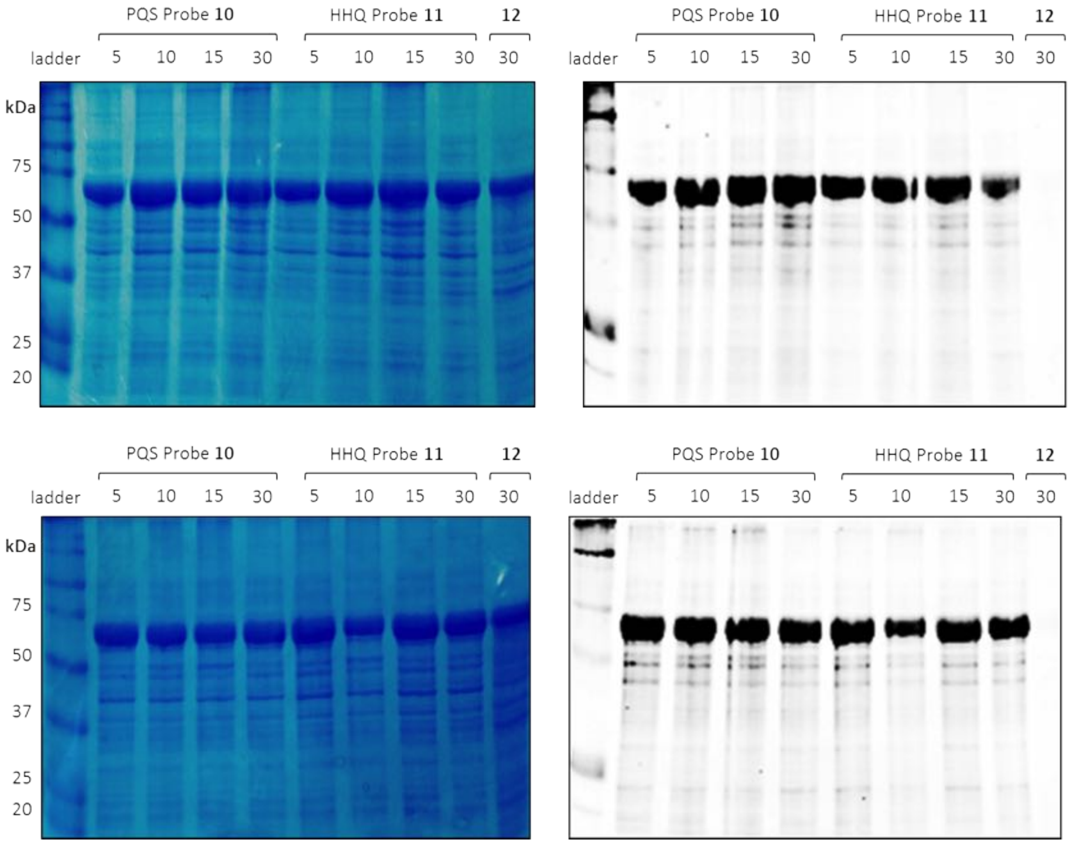

Supplementary Figure 1. Cell-lysate based labelling of $PqsR_{LBD}$-MBP using λ = 302 nm (Top) and 365 nm (bottom). Time of irradiation in minutes is given above each lane. 30 min irradiation was used in the case of control probe **12.** Left) gel stained with Coomassie blue; right) visualisation by fluorescent gel imaging. Time of irradiation is given in minutes.

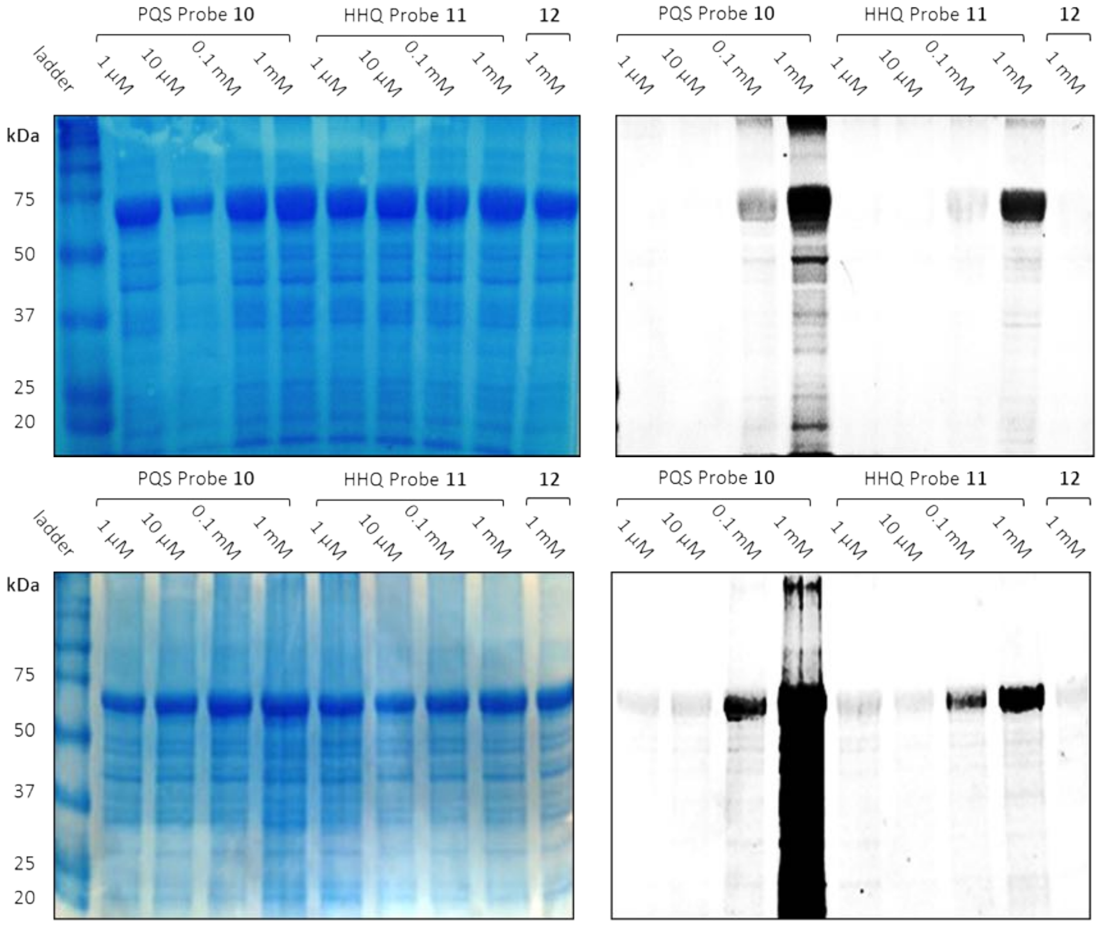

Supplementary Figure 2. Labelling of PqsR$_{LBD}$-MBP in live *E. coli* using λ = 302 nm (top) and 365 nm (bottom). Concentration of probe is given above each lane. Left) gel stained with Coomassie blue; right) visualization by fluorescent gel imaging.

1.2 Growth curves and harvesting of the bacterial strains used in this study

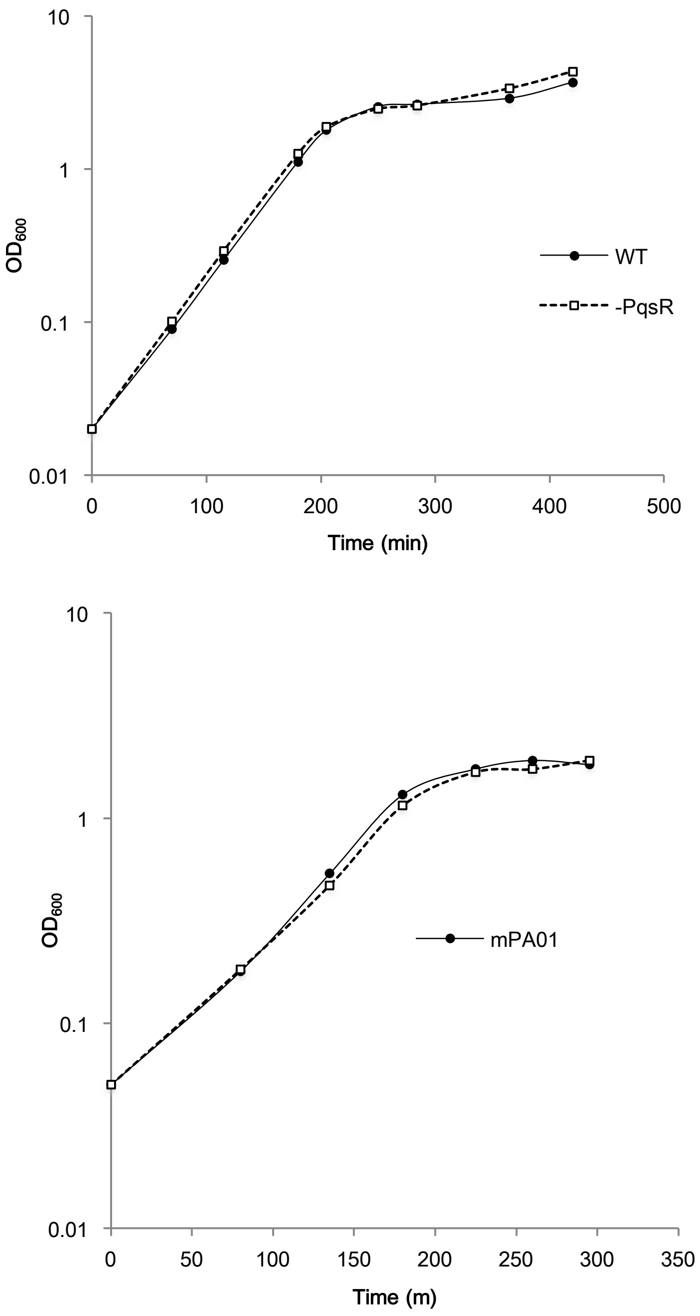

Supplementary Figure 3. Growth curve of cells that were harvested for pull-down experiments. A) Late exponential phase time point; B) Early stationary phase time point.

1.3 SDS PAGE analysis of pull-downs

Late exponential phase pull-downs. Cells were harvested at late exponential phase by centrifugation (Time point A, Supplementary Fig. 3). Late exponential phase was chosen initially as this lies just before the point at which the QS genes are “switched” on. A second reason for this is that Cao et al. reported that PqsR is at maximal levels during late exponential phase growth and is then cleaved and secreted into the supernatant in the stationary phase.[2] The harvested cells were lysed and centrifuged at low speed, keeping both the pellet and the supernatant.

PQS probe **10**, HHQ probe **11** and control probe **12** were used to prepare samples for MS identification. Cell-lysates were incubated with the probes at 10 μM for 30 min before irradiation with UV light (302 nm, 15 min). Copper catalysed click chemistry was used it tag a biotin alkyne to the labelled proteins. The protein samples were then incubated with streptavidin-coated magnetic beads. The beads were then washed stringently and the captured proteins eluted by boiling in gel loading buffer containing SDS and free biotin. The proteins were then separated by SDS-PAGE and silver-stained (Supplementary Fig. 4).

In addition competitive binding experiments were also performed experiment. In this case, 100 μM of PQS was added to a second sample with 10 μM of PQS probe **10** and 100 μM of HHQ was added to a second experiments using 10 μM of HHQ probe **11**. The pull-downs were performed identically to those described above.

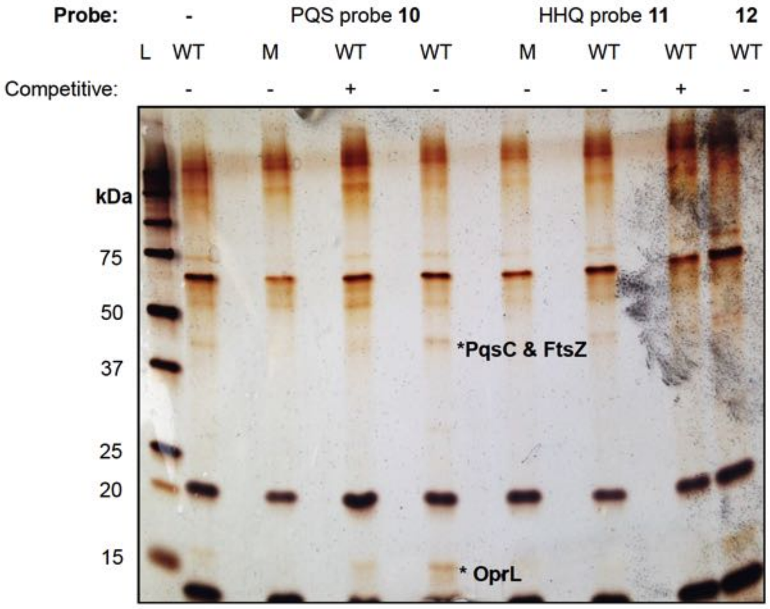

Supplementary Figure 4. Silver stain of proteins isolated from late exponential phase labelled using probes **10**, **11** and **12**. Bands marked with an * were analysed by tryptic digest followed by LC-MS. + indicates the competitive control lane. WT = wild type cell-lysate; M = *pqsR* mutant cell-lysate.

For analysis, the proteins identified in the control lane of the gel at the same migration distance and proteins found in on-bead digest negative control experiments (discussed in further detail later) were removed. Ribosomal proteins were also disregarded, as they are known to be highly abundant and stick to the beads used.

The band at around 40 kDa had evidence of FtsZ (Mascot score of 80) and PqsC (Mascot score 39), a protein required for the synthesis of HHQ and PQS. Unfortunately, the LCMS trace for this sample had low levels of material. Despite this, PqsC had the highest scoring peptide (Supplementary Table 9).

The second band analysed at approximately 17 kDa was found to contain 27 potential proteins, many of which are presumably fragments of larger proteins. For this reason only the proteins that were also identified in the later on-bead digest were considered. The only protein from this list with convincing peptide scores was peptidoglycan-associated lipoprotein pal (also referred to as OprL in the literature) with a Mascot score of 85 and the correct mass for the migration by SDS-PAGE.

The evidence for the pull-down of OprL using the PQS probe **10** is strong, with three high-scoring peptides and the correct migration distance for the protein's molecular weight. The band is also intense when compared with the corresponding competitive binding assay. The band is not seen for probe **11** suggesting the protein interacts with PQS and not HHQ, again supporting the validity of this result. As the band can be seen with probe **12** it is also possible that it is protein that is under QS control which binds lipophilic compounds. Whilst further biochemical studies are required to confirm that PQS does in fact bind OprL, the pull-down of this protein is promising; one possible hypothesis is that the interaction between PQS and OprL is linked to membrane vesicle (MV) formation.

The role of PQS in membrane vesicle formation has been well documented. It has previously been shown that PQS stimulates membrane vesicle formation and it has been proposed that this is through direct association with the lipopolysaccharide in the outer membrane.[3] Multiple studies have identified that MV formation occurs in regions where the membrane is not tethered to the peptidoglycan and is has been suggested that the loss of connections between the peptidoglycan layer and lipoproteins (LP) such as OprL are necessary for MV formation.[4] In fact, loss of the 18 kDa pal homologue in *E. coli* has been shown to significantly increase MV formation.[4]

Building on this hypothesis, Wessel et al. demonstrated that deletion of the peptidoglycan-associated outer membrane proteins OprF and OprI, but not OprL, impact production of MVs by the opportunistic pathogen *P. aeruginosa*.[5] If PQS does bind OprL (as is suggested by this pull-down), the interaction between OprL and PQS might be important in controlling MV formation.

2 Supplementary data

2.1 Biological data

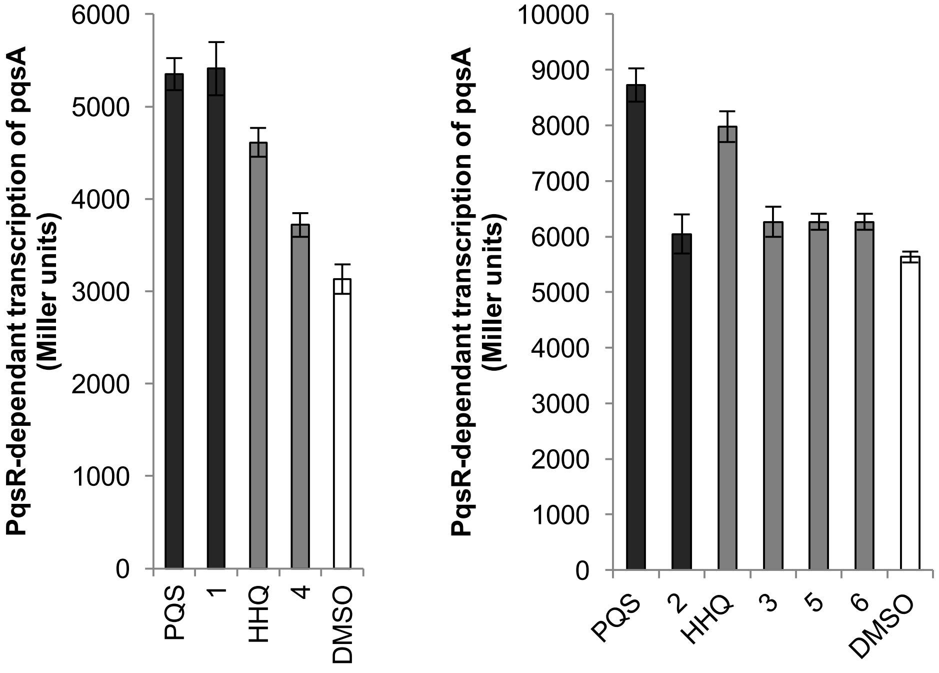

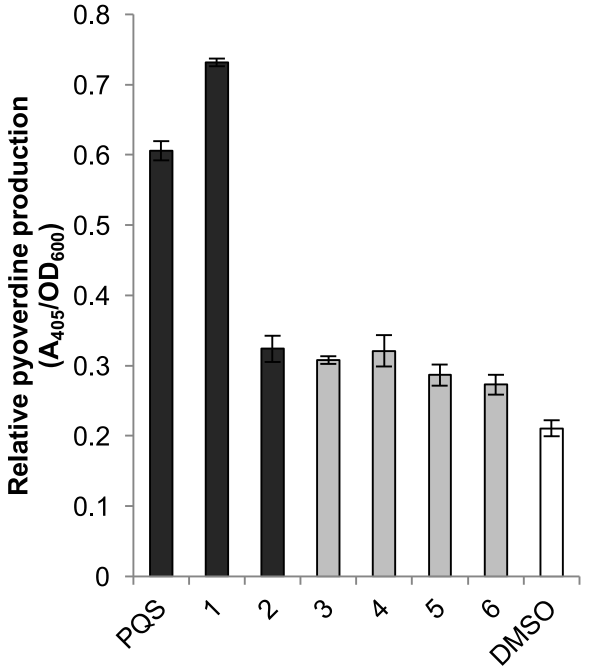

Supplementary Figure 5. Effect of introducing ligation handles groups on quinolone activity. A) PqsR stimulation determined using the method reported by Cugini et al.[6] These compounds were tested. B) Pyoverdine production determined using the method reported by Welsh et al.[7] Dark bars show PQS analogues which were tested at 60 nM and light bars show HHQ analogues which were tested at 1 μM. Error bars represent the standard deviation of three independent repeats.

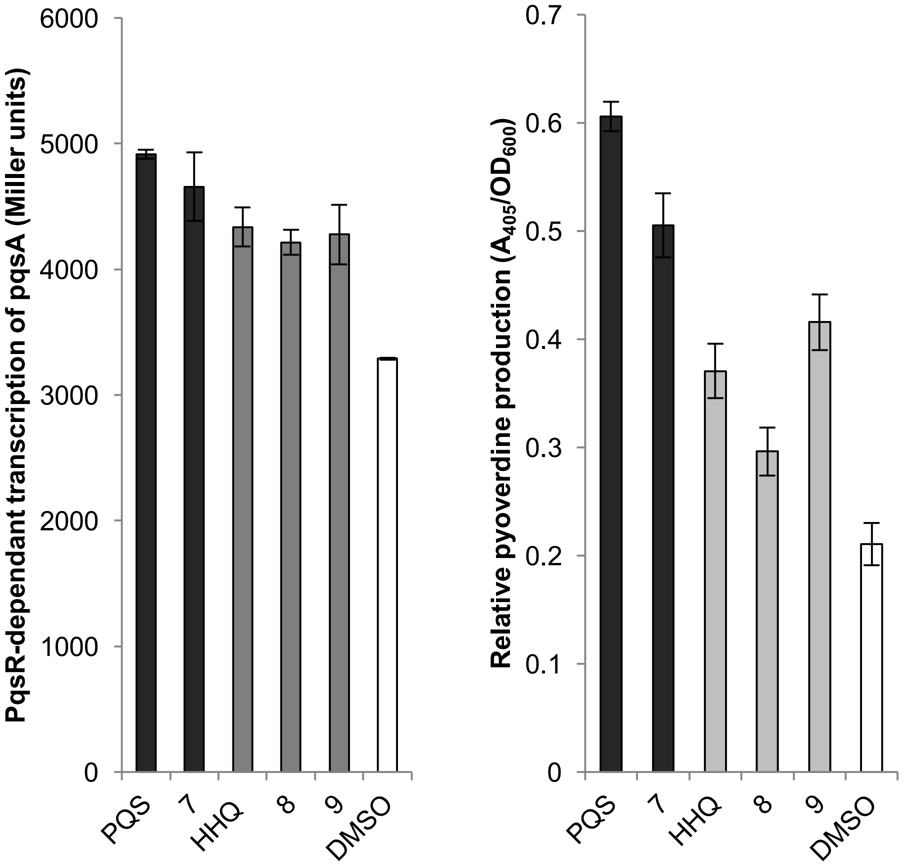

Supplementary Figure 6. Effect of introducing photoreactive groups on quinolone activity. A) PqsR stimulation determined using the method reported by Cugini et al.[6] B) Pyoverdine production determined using the method reported by Welsh et al.[7] Dark bars show PQS analogues which were tested at 60 nM and light bars show HHQ analogues which were tested at 1 μM. Error bars represent the standard deviation of three independent repeats.

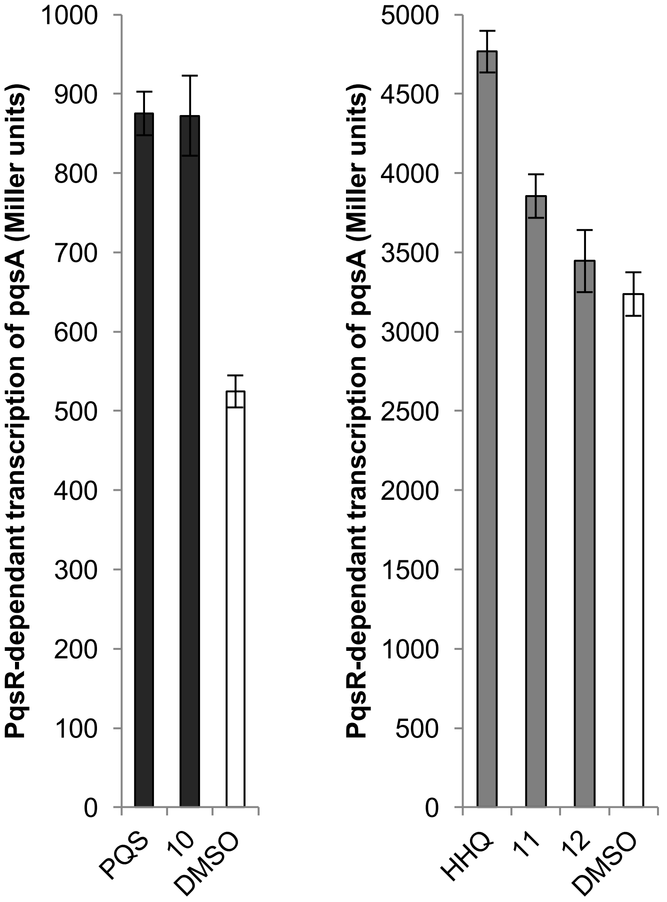

Supplementary Figure 7. Biological activity of final probes. PqsR stimulation determined using the method reported by Cugini et al.[6]

3 Supplementary tables

3.1 Peptide tables for proteins in text and supplementary discussion

Supplementary Table 2. Peptide table for proteins identified using PQS probe **10** in the wild type strain.

accession	prot_desc	prot_score	prot_mass	prot_cover	pep_exp_mz	pep_exp_mr	pep_exp_z	pep_calc_mr	pep_delta	pep_score	pep_expect	pep_seq	prot_empai
G3XD23 G3XD23	WbpB: UDP-N-acetyl-2-amino-2-deoxy-D-glucuronate oxidase	253	35980	18	635.8074	1269.6002	2	1269.5989	0.0013	63.04	0.00000079	AAGYLEYEQAR	0.37
					680.3417	1358.6689	2	1358.6718	-0.0028	59.87	0.0000033	YEVDLTYITSR	0.37
					717.3984	1432.7823	2	1432.7827	-0.0003	72.62	0.00000018	NFALIGAAGYIAPR	0.37
					707.7087	2120.1042	3	2120.1014	0.0028	23.71	0.014	SAVIVPASDNEGHPFVAALAR	0.37
Q9I246	PA2069 : Probable carbamoyl transferase	88	64141	7.5	596.8128	1191.6111	2	1191.6135	-0.0024	66.73	0.00000076	ITGLAAFGDAEK	0.09
					681.6595	2041.9567	3	2041.9606	-0.0038	22.34	0.009	SIVYHPGDASANDWLNQR	0.09
P47204	FtsZ: Cell division protein FtsZ	90	41306	13.2	719.8871	1437.7597	2	1437.7576	0.0021	59.27	0.0000042	NPLLEDVNLQGAR	0.07
G3XD12	HcnC: Hydrogen cyanide synthase subunit HcnC	85	45766	5	546.7877	1091.5609	2	1091.5611	-0.0001	85.2	8.8E-09	LADAYLEAAR	0.06
Q7DC80	PhzD1: Phenazine biosynthesis protein PhzD1	83	23352	12.6	506.2501	1010.4857	2	1010.4855	0.0002	67.97	0.00000022	MALEYAASR	0.27
					955.9904	1909.9662	2	1909.9673	-0.0011	30.52	0.0027	EVVEELAPGPDDWLLTK	0.27
O69754	PhzF1 Trans-2,3-dihydro-3-hydroxyanthranilate isomerase	78	30551	9.4	389.7258	777.4371	2	777.4385	-0.0014	27.93	0.0038	LFLETR	0.32
					770.3634	2308.0684	3	2308.0754	-0.007	39.62	0.00011	EMNLSESTFVLRPQQDGDAR	0.32
Q9I6D8	PfpI : Protease PfpI OS=Pseudomonas aeruginosa	69	19291	11.2	713.6606	2137.96	3	2137.9665	-0.0065	45.26	0.000031	DDINNAGGHWVDQEVAVDGK	0.15
P20582	PqsD : 2-heptyl-4(1H)-quinolone synthase PqsD	37	36698	7.4	661.8332	1321.6519	2	1321.6514	0.0006	37.15	0.00052	INTSDEFIVER	0.08
				7.4	661.8332	1321.6519	2	1321.6514	0.0006	37.15	0.00052	INTSDEFIVER	0.08
O50175	AstB: N-succinylarginine dihydrolase	37	49300	7.1	698.3391	1394.6637	2	1394.6678	-0.004	36.69	0.00049	SLGFSGSDEEVIR	0.06

P54292	RhlR: Regulatory protein RhlR	26	27731	27.8	774.8812	1547.7479	2	1547.7521	-0.0042	25.81	0.0062	NDGGFLLWWDGLR	0.11
Q9I4X1	PqsC: 2-heptyl-4(1H)-quinolone synthase PqsC	35	38613	3.4	436.902	1307.6843	3	1307.6833	0.0009	34.83	0.00095	LPENPTGEAKPR	0.08

Supplementary Table 3. Peptide table for proteins identified using PQS probe **10** in the pqsR mutant strain.

accession	prot_desc	prot_score	prot_mass	prot_cover	pep_exp_mz	pep_exp_mr	pep_exp_z	pep_calc_mr	pep_delta	pep_score	pep_expect	pep_seq	prot_empai
G3XD23	WbpB: UDP-N-acetyl-2-amino-2-deoxy-D-glucuronate oxidase	359	35980	26.9	516.8127	1031.6108	2	1031.6127	-0.0019	49.46	0.000016	LYNILQLR	0.6
					614.8078	1227.601	2	1227.603	-0.002	34.74	0.00077	HCVETVNTIR	0.6
					635.8071	1269.5997	2	1269.5989	0.0008	44.05	0.000061	AAGYLEYEQAR	0.6
					680.3419	1358.6693	2	1358.6718	-0.0025	65.52	0.0000009	YEVDLTYITSR	0.6
					717.3988	1432.783	2	1432.7827	0.0004	52.68	0.000017	NFALIGAAGYIAPR	0.6
					707.7087	2120.1044	3	2120.1014	0.0029	43.19	0.00015	SAVIVPASDNEGHPFVAALAR	0.6
P47204	FtsZ: Cell division protein FtsZ	94	41306	6.3	719.8858	1437.7571	2	1437.7576	-0.0005	63.25	0.0000016	NPLLEDVNLQGAR	0.07
P54292	RhlR Regulatory protein RhlR	100	27731	32	662.3218	1322.629	2	1322.6295	-0.0006	44.8	0.000056	LGFDYYAYGVR	0.35
					677.3969	1352.7792	2	1352.7776	0.0016	64.65	0.00000086	APNNLLSVLSVAR	0.35

Supplementary Table 4. Peptide table for proteins identified using PQS probe **10** in the rhlR mutant strain.

accession	prot_desc	prot_score	prot_mass	prot_cover	pep_exp_mz	pep_exp_mr	pep_exp_z	pep_calc_mr	pep_delta	pep_score	pep_expect	pep_seq	prot_empai
G3XD23	WbpB: UDP-N-acetyl-2-amino-2-deoxy-D-glucuronate oxidase	56	35980	17.7	860.4328	1718.8511	2	1718.8515	-0.0004	55.8	0.0000088	WFLSVDANDLPESVK	0.19
					707.7067	2120.0984	3	2120.1014	-0.003	18.12	0.054	SAVIVPASDNEGHPFVAALAR	0.19
P47204	FtsZ: Cell division protein FtsZ	34	41306	2	549.2892	1096.5638	2	1096.5625	0.0013	21.31	0.017	GLGAGANPEVGR	0.08
					549.2894	1096.5642	2	1096.5625	0.0017	28.3	0.0034	GLGAGANPEVGR	0.08

Supplementary Table 5. Peptide table for proteins identified using HHQ probe **11** in the wild type strain.

accession	prot_desc	prot_score	prot_mass	prot_cover	pep_exp_mz	pep_exp_mr	pep_exp_z	pep_calc_mr	pep_delta	pep_score	pep_expect	pep_seq	prot_empai
P47204	FtsZ: Cell division protein FtsZ	25	41306	7.4	549.2877	1096.561	2	1096.563	-0.0016	24.67	0.008	GLGAGANPEVGR	0.08
O50175	AstB: N-succinylarginine dihydrolase	48	49300	22.3	537.2839	1072.553	2	1072.54	0.0131	12.34	0.25	DQVAVEDAVK	0.07
					622.8582	1243.702	2	1243.702	-0.0005	34.6	0.0012	TALDELTQILK	0.07

Supplementary Table 6. Peptide table for proteins identified using HHQ probe 11 in the pqsR mutant strain.

accession	prot_desc	prot_score	prot_mass	prot_cover	pep_exp_mz	pep_exp_mr	pep_exp_z	pep_calc_mr	pep_delta	pep_score	pep_expect	pep_seq	prot_empai
P47204	FtsZ: Cell division protein FtsZ	25	41306	7.6	549.2889	1096.563	2	1096.563	0.0008	24.84	0.0079	GLGAGANPEVGR	0.08
G3XD23	WbpB: UDP-N-acetyl-2-amino-2-deoxy-D-glucuronate oxidase	22	35980	12.7	635.8054	1269.596	2	1269.599	-0.0027	18.65	0.02	AAGYLEYEQAR	0.09

Supplementary Table 7. Peptide table for proteins identified using HHQ probe **11** in the rhlR mutant strain.

accession	prot_desc	prot_score	prot_mass	prot_cover	pep_exp_mz	pep_exp_mr	pep_exp_z	pep_calc_mr	pep_delta	pep_score	pep_expect	pep_seq	prot_empai
G3XD23	WbpB: UDP-N-acetyl-2-amino-2-deoxy-D-glucuronate oxidase	32	35980	5	860.4332	1718.852	2	1718.852	0.0003	32.42	0.0019	WFLSVDANDLPESVK	0.09
P47204	FtsZ: Cell division protein FtsZ	48	41306	13.5	549.2873	1096.56	2	1096.563	-0.0024	41.79	0.00016	GLGAGANPEVGR	0.08

Supplementary Table 8. Peptide table for proteins identified using PQS probe **10** wild type during late exponential phase as identified from in-gel digestion of band at around 40 kDa (Supplementary Fig. 4).

accession	prot_desc	prot_score	prot_mass	prot_cover	pep_exp_mz	pep_exp_mr	pep_exp_z	pep_calc_mr	pep_delta	pep_score	pep_expect	pep_seq	prot_empai
P47204	FtsZ: Cell division protein FtsZ	60	41306	8.2	549.2893	1096.564	2	1096.563	0.0016	46.06	0.00057	GLGAGANPEVGR	0.07
					719.8854	1437.756	2	1437.758	-0.0014	41.86	0.00022	NPLLEDVNLQGAR	0.07

Q9I4X1	PqsC 2-heptyl-4(1H)-quinolone synthase PqsC	39	38613	3.2	632.3192	1262.624	2	1262.626	-0.0016	38.82	0.00044	NGENEFSLVVR	0.08

Supplementary Table 9. Peptide table for proteins identified using PQS probe **10** wild type during late exponential phase as identified from in-gel digestion of band at around 17 kDa (Supplementary Fig. 4).

accession	prot_desc	prot_score	prot_mass	prot_cover	pep_exp_mz	pep_exp_mr	pep_exp_z	pep_calc_mr	pep_delta	pep_score	pep_expect	npep_seq	prot_empai
Q9I4Z4	Pal: Peptidoglycan-associated lipoprotein	85	17971	26.8	549.7465	1097.479	2	1097.481	-0.0026	39.36	0.00012	EYNMALGER	0.58
					385.5317	1153.573	3	1153.573	0.0004	19.82	0.026	VVLEGHTDER	0.58
					800.7072	2399.1	3	2399.099	0.0007	26.56	0.0022	AITTFYFEYDSSDLKPEAMR	0.58

4 Supplementary methods

4.1 Biological experimental section

General. A Jenway 6705 spectrometer and 1 cm path length cuvettes were used for OD_{600}, A_{405}, A_{420} and A_{550} measurements. cOmplete™, Mini, EDTA-free protease inhibitor cocktail tablets were purchased from Roche and used in accordance with the manufacturers guidelines. TAMRA-alkyne (5/6-Carboxytetramethylrhodamine-PEG_4-Alkyne) was purchased from Jenna Biosciences and was used without further purification. Biotin alkyne without a PEG linker was used (molecular formula $C_{13}H_{19}N_3O_2S$, Supplementary Figure 8). UV irradiation steps were carried out using an inverted 3UV transilluminator at a distance of 2 cm, keeping the samples cooled on ice. SDS gels were hand cast using a Bio-rad Mini-PROTEAN® Tetra Handcast System with a thickness of 0.75 mm. All fluorescence images of SDS-gels were recorded using a Typhoon 9000 using the 532 nm laser, 800 PMT and 50 μm resolution. Unless otherwise stated all solutions were in water. MilliQ water was used in the preparation of all samples for LC/MS analysis.

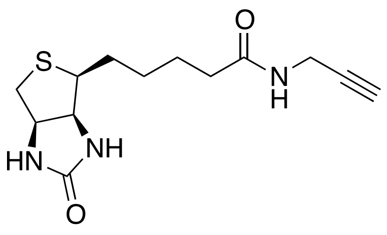

Supplementary Figure 8. Structure of biotin alkyne used throughout this study.

Bacterial strains and growth conditions. The bacterial strains used in this study are listed in Supplementary Table 10. Unless otherwise stated the bacteria were grown as follows: Desired strains were struck on a LB agar plate and grown overnight at 37 °C. Single colonies were then used to inoculate 10 mL of LB media and grown overnight at 37 °C. These were used to inoculate cultures to a starting OD_{600} of 0.05 which were then grown in either an incubator or a water bath at 37 °C with good aeration.

Strain	Description	Source
P. aeruginosa		
PAO1	wild type	Iglewski et al.[8]
ΔpqsR	PqsR mutant in a PAO1 background	Pesci et al.[9]
mPAO1	wild type	Manoil et al.[10]
ΔrhlR/rhlI	RhlR mutant in a PAO1 background. rhlRI::lRIl CNK	Mattick et al.[11]
E. coli		
JTH1	*E. col.i* Contains the plasmid pMAL-c2X with $PqsR_{LBD}$-MBP insert; ampicillin resistant.	Welch et al.[1]
DH618	*E. coli.* Contains the plasmid pEAL08-2 with the PqsR expression vector and *pqsA'-lacZ* transcriptional fusion; PqsR reporter vector; ampicillin resistant.	Hogan et al.[6]

Supplementary Table 10. Bacterial strains used in this study.

***pqsA::lacZ* transcriptional assay.** Quinolone analogues were tested for their PqsR agonist activity by measuring transcription of the *pqsA* promoter region in an *E. coli* DH5α strain DH618 containing the pEAL08-2 plasmid as previously described.[6] *E. coli* strain DH618 was always grown in the presence of carbenicillin (50 µg/mL). Overnight cultures were diluted 1:50 into LB media and grown to an OD_{600} of 0.2 (~2.5 h). Quinolone analogues in DMSO (50 µL) were added to 5 mL cultures and the cultures grown for a further 2.5 h before measuring the OD_{600} and β galactosidase activity quantified.

Pyoverdine assay. Pyoverdine was quantified as previously described.[12] Overnight cultures of PAO1 were diluted 1:100 into LB medium and quinolone analogues in DMSO (25 µL) were added to the subcultures (2.5 mL). The cultures were grown with good aeration for 17 h before measuring the OD_{600}. The cells were pelleted by centrifugation (13000 xg, 4 °C, 10 min), 200 µL of the supernatant was transferred to a clear 96 well microtiter plate and the absorbance at 405 nm was measured and the values corrected for the cell density.

PqsRLBD labelling in *E. coli* cell lysate. *E. coli* cells containing a pMAL-c2X plasmid with a PqsRLBD-MBP insert were grown and protein expression induced as previously reported.[1] Cells were harvested by centrifugation (7400 xg, 10 min, 4 °C), washed twice with ice cold

phosphate-buffered saline (PBS) and the pellets stored overnight at -18 °C. Cells were allowed to thaw on ice, re-suspended in ice cold PBS containing a complete, mini, EDTA free protease inhibitor cocktail tablet (Roche) and lysed by ultrasonication (10 x 30 s) taking care to keep the samples below 4 °C. The samples were then clarified using low speed centrifugation (7400 xg, 10 min, 4 °C) and the protein concentration determined using the Bradford protein assay. Protein concentration was adjusted to 5 mg/mL using PBS containing a protease inhibitor cocktail tablet and samples were snap frozen using liquid nitrogen and stored at -80 °C until use. The probes were added as stock solutions in DMSO (2 µL, 0.1 M) to 0.2 mL of cell-lysate and these were incubated at room temperature for 30 min with gentle shaking. The samples were then transferred to a 12 well flat bottomed plate and irradiated with UV light (302 nm) for 15 min at a distance of approximately 0.5 cm using an inverted 3UV benchtop transilluminator whilst being cooled on ice.

PqsRLBD labelling in live *E. coli*. Cultures (10 mL) of *E. coli* containing a pMAL-c2X plasmid with a PqsRLBD-MBP insert were grown and protein expression induced as previously reported.[1] 30 min prior to harvesting the cells the photoreactive probes in DMSO (10 mM, 100 µL) were added to the cultures to a final concentration of 100 µM. Cells were harvested by centrifugation (7400 xg, 20 min, 4 °C) and washed twice with PBS at 4 °C. The cell pellets were re-suspended in PBS (2 mL), transferred to a 6 well plate and irradiated with UV light of 302 nm using a 3UV transiluminator for 15 min at 4 °C. The cells were then collected by centrifugation (7400 xg, 30 min, 4 °C), the supernatant discarded and the cells stored at -80 °C overnight. Cells were thawed on ice, re-suspended PBS (1 mL) containing a protease inhibitor cocktail tablet (Roche). Cells were lysed by ultrasonication (3 x 30 s) while keeping the samples below 4 °C, clarified by centrifugation (15,000 xg, 30 min, 4 °C) and the supernatant containing the PAL proteins was collected and stored at -80 °C until use. These were then labelled with TAMRA or biotin.

Click chemistry tagging of labelled proteins. Tris[(1-benzyl-1H-1,2,3-triazol-4-yl)methyl]amine (TBTA) (5 µL, 1.6 mM solution in DMSO), $CuSO_4$ (1 µL, 0.1 M in H2O), tris-(2-carboxyethyl)phosphine (TCEP) (1 µL, 0.1 M in H2O) and biotin alkyne (1 µL, 2 mM in DMSO) or 5/6-TAMRA-PEG_4-alkyne (Jena Bioscience) (1 µL, 2 mM in DMSO) were added to 100 µL of probe PAL sample. This was incubated with vigorous shaking for 2 h at 25 °C. The reaction was then quenched by the addition of EDTA (10 µL, 0.5 M).

Fluorescence visualisation of TAMRA tagged protein. Proteins were precipitated with trichloroacetic acid (60 µL, 50% (w/v) H_2O) and collected by centrifugation (7400 xg, 20 min, 4 °C). The proteins pellet was washed with acetone/water (1:1, 2 x 200 µL), water (2 x 200 µL) and air dried for 5 min. Proteins were then denatured by heating to 95 °C in sample loading buffer for 10 min and resolved using 12% SDS PAGE. Labelled bands were visualised using gel fluorescence scanning with a Typhoon 9000 using the 532 nm laser, 800 PMT and 50 µm resolution, before staining with Coomassie to visualise total protein content.

Pull-down of biotin tagged PqsR. Biotin tagged proteins were precipitated using the $CHCl_3$/MeOH procedure.[13] Pull-downs were then performed as outlined previously.[14] Briefly, SDS (10 µL, 10% w/v in H_2O) was added to the biotin labelled protein pellets the sample heated to 100 °C for 5 min. This was diluted with water (4 x 50 µL) with vortexing after each addition. The proteins were precipitated using the $CHCl_3$/MeOH procedure[13] and the resulting protein pellet was solubilised in 1% SDS in 100 mM NH_4HCO_3 (200 µL) with vigorous shaking for 30 min. Any remaining insoluble protein was removed by centrifugation (7400 xg, 10 min, 4 °C) and the samples were then separated by SDS-PAGE using a 12% gel and stained with Coomassie to visualise the pull-down.

Preparation of *P. aeruginosa* cell lysates. *P. aeruginosa* strains were struck on a LB agar plate and grown overnight at 37 °C. Single colonies were then used to inoculate 10 mL of LB media and grown overnight at 37 °C. These were used to inoculate cultures to a starting OD_{600} of 0.05 which were then grown to early stationary phase in a water bath at 37 °C with good aeration. Cells were harvested by centrifugation (7400 xg, 10 min, 4 °C), washed twice with ice cold phosphate-buffered saline (PBS) and the cell pellets stored over night at -18 °C. Cells were allowed to thaw on ice before being resuspended in ice cold PBS containing a cOmplete, mini, EDTA free protease inhibitor cocktail tablet and lysed by ultrasonication (10 x 30 s) taking care to keep the samples below 4 °C. The protein samples were then clarified using low speed centrifugation (7400 xg, 10 min, 4 °C) and the protein concentration determined using the Bradford protein assay before being snap frozen using liquid nitrogen and stored at -80 °C until use.

Cell-lysate photocrosslinking and labelling with biotin. Cell-lysates were adjusted to a protein concentration of 5 mg/mL using PBS containing a protease inhibitor cocktail tablet (Roche). The probes were then added (10 µM final concentration from 1 mM stock solution in DMSO) to 0.2 mL of protein sample, which were then incubated at room temperature for 30 min with gentle shaking. The samples were then irradiated with UV light (λ = 302 nm) for 15 min. Click chemistry with biotin alkyne was used to install biotin (as described earlier) and the proteins were precipitated using a Chloroform/Methanol precipitation.[13]

4.2 Pull-down experiments for protein identification

Pull-down of biotin tagged proteins. Biotin tagged proteins were precipitated using the $CHCl_3$/MeOH procedure.[13] Pull-downs were then performed as outlined previously.[14] Briefly, SDS (10 µL, 10% w/v in H_2O) was added to the biotin labelled protein pellets the sample heated to 100 °C for 5 min. This was diluted with water (4 x 50 µL) with vortexing after each addition. The proteins were precipitated using the $CHCl_3$/MeOH procedure[13] and the resulting protein pellet was solubilised in 1% SDS in 100 mM NH_4HCO_3 (200 µL) with vigorous shaking for 30 min. Any remaining insoluble protein was removed by centrifugation (7400 xg, 10 min, 4 °C) and the samples were reduced by the addition of DTT (1 µL, 1 M in H_2O) and incubating

the samples at 37°C for 30 min. Iodoacetamide (15 µL, 200 mM in 1% (w/v) SDS in 100 mM NH_4HCO_3) was added and the samples incubated at 37 °C for 30 min in the dark. Any insoluble protein was removed by centrifugation (7400 xg, 10 min, 4 °C) and a second $CHCl_3$/MeOH precipitation was performed.[13] The protein pellet was solubilised in 2% (w/v) SDS in 100 mM NH_4HCO_3 (50 µL) using a sonic water bath and heating to 70 °C. This was diluted with 50 mM NH_4HCO_3 (50 µL) and vortexed for 2 min. The dilution was repeated twice more before the addition of 50 mM NH_4HCO_3 (800 µL). Any insoluble protein was removed by centrifugation (7400 xg, 10 min, 4 °C) and the protein sample incubated for 2 h with inversion at room temperature with pre-equilibrated MyOne T1 Streptavidin grafted magnetic beads (Invitrogen) (50 µL) pre-equilibrated according to manufacturer's instructions. The beads were stringently washed with H_2O (2 x 200 µL), PD buffer (50 mM Tris-HCl, 150 mM NaCl, pH 7.5) with 0.1% SDS (2 x 100 µL), PD buffer (2 x 200 µL), wash buffer I (4 M urea, 50 mM NH_4HCO_3) (1 x 200 µL), wash buffer II (50 mM Tris-HCl, 10 mM NaCl, pH 7.5) (1 x 200 µL) and water (1 x 200 µL). In the case of the PqsRLBD live cell labelling experiment, the bound proteins were eluted from the beads by boiling in loading buffer containing 10 µM biotin (50 µL) for 10 min. The sample was then separated by SDS-PAGE using a 12% gel and stained with Coomassie to visualise pull-down.

LC/MS protein identification and analysis. The beads from the pull-down experiment were washed twice with PBS, twice with digestion buffer (100 mM NH_4HCO_3 / 50 mM NaCl), taken up in 100 µL digestion buffer with 200 ng MS-grade trypsin (Promega) and digested overnight at 37°C. Formic acid (2 µL) was added to achieve a pH of 2-3 for inhibiting the trypsin activity and the peptides were desalted with Stagetips prior to LC-MS analysis. Tryptic peptides were analysed on a Surveyor nanoLC system (Thermo) hyphenated to an LTQ-Orbitrap mass spectrometer (Thermo). Gold and carbon coated emitters (OD/ID = 360/25 µm tip ID = 5 µm), trap column (OD/ID = 360/100 µm packed with 25 mm robust Poros10R2/ 15 mm BioSphere C18, 5 µm, 120 Å beads) and analytical columns (OD/ID = 360/75 µm packed with 20 cm BioSphere C18, 5 µm, 120 Å beads) were from Nanoseparations (Nieuwkoop, The Netherlands). The mobile phases (A: 0.1% formic acid/H_2O, B: 0.1% formic acid/MeCN) were made with ULC/MS grade solvents (Biosolve). The emitter tip was coupled end-to-end with the analytical column via a 15 mm long TFE Teflon tubing sleeve (OD/ID 0.3 x 1.58 mm, Supelco, USA) and installed in a stainless steel holder mounted in a nano-source base (Upchurch scientific, Idex, USA). General mass spectrometric conditions were as follows: an electrospray voltage of 1.8 kV was applied to the emitter, no sheath and auxiliary gas flow, ion transfer tube temperature 150 °C, capillary voltage 41 V, tube lens voltage 150 V. Internal mass calibration was performed with air-borne protonated polydimethylcyclosiloxane (m/z = 445.12002) and the plasticiser protonated dioctyl phthalate ions (m/z = 391.28429) as lock mass. For shotgun proteomics analysis, 10 µL of the samples was pressure loaded on the trap column with a 10 µL/min flow for 5 min followed by peptide separation with a gradient of 35 min 5%–30% B, 15 min 30%–60% B, 5 min A at a flow of 300 µL/min split to 250 nL/min

by the LTQ divert valve. For each data-dependent cycle, one full MS scan (300–2000 m/z) acquired at high mass resolution (60,000 at 400 m/z, AGC target 1x106, maximum injection time 1000 ms) in the Orbitrap was followed by three MS/MS fragmentations in the LTQ linear ion trap (AGC target 5x104, maximum injection time 120 ms) from the three most abundant ions. MS2 settings were as follows: collision gas pressure 1.3 mT, normalised collision energy 35%, ion selection threshold of 750 counts, activation q = 0.25 and activation time of 30 ms. Fragmented precursor ions that were measured twice within 10 s were dynamically excluded for 120 s and ions with z < 2 or unassigned were not analysed. For peptide identification, mascot generic files (mgf) were generated from the .raw data files and matched with the Mascot (Matrix science) search engine to the *Pseudomonas aeruginosa* database extracted from Uniprot, using a reversed decoy database to estimate the false discovery rate, 20 ppm peptide tolerance for the selection of the parent ion, 0.8 Da MS/MS tolerance, trypsin cleavage allowing for two miss-cleavages, mono-isotopic peptide selection, carbamidomethyl addition to cysteines (57.0513 Da) as fixed and oxidation of methionine (15.9994 Da) as variable modifications. The protein lists were then compared using Microsoft Excel and proteins present in the control samples were identified and removed.

4.3 Synthetic experimental

General. Unless otherwise stated, reactions were performed in oven-dried glassware under nitrogen with dry, freshly distilled solvents. THF was distilled from $LiAlH_4$ in the presence of triphenyl methane indicator. CH_2Cl_2, hexane, MeOH, pyridine and MeCN were distilled from calcium hydride. Anhydrous DMF (Acros) and DCE (Aldrich) were used without further purification. All other chemicals were used as obtained from commercial sources. Biotin alkyne without a PEG linker was used (molecular formula $C_{13}H_{19}N_3O_2S$, see Supplementary Figure 8). Yields refer to analytically pure compounds. TLC was performed using Merck pre-coated 0.23 mm thick plates of Keiselgel 60 F_{254} and visualised using UV (λ = 254 nm) or by staining with $KMnO_4$. All retention factors (R_f) are given to 0.01. All column chromatography was carried out using Merck 9385 Keiselgel 60 silica gel (230-400 mesh). ^{1}H NMR spectra were recorded on Bruker DPX 400 or 500 spectrometers operating at 400 or 500 MHz respectively using an internal deuterium lock at ambient probe temperatures. Chemical shifts (δ) are quoted to the nearest 0.01 ppm and are reference relative to residual solvent peak.[15] Coupling constants (*J*) are given to the nearest 0.1 Hz. The following abbreviations are used to indicate the multiplicity of signals: s = singlet, d = doublet, t = triplet, q = quartet, m = multiplet and br = broad. Data is reported as follows: chemical shift (multiplicity, coupling constant(s), integration). ^{13}C NMR spectra were recorded on Bruker 400 or 500 spectrometers operating at 100 and 125 MHz respectively using an internal deuterium lock at ambient probe temperatures. Chemical shifts (δ) are quoted to the nearest 0.1 ppm and are reference relative to the deuterated solvent peak.[15] High resolution mass spectra (HRMS) were recorded using either a Micromass Q-TOF or a Micromass LCT Premier spectrometer and reported mass values are within ± 5 ppm mass units unless otherwise stated. Low

resolution mass spectra were recorded on an HP/Agilent LCMS APCI 120-1000 full gradient machine. IR spectra were recorded using neat sample on a Perkin-Elmer 1600 FT IR spectrometer. Selected absorption maxima are reported in wavenumbers (cm^{-1}). Melting points (mp) were measured using a Buchi B-545 melting point apparatus and are uncorrected.

Synthetic procedures

PQS and HHQ were synthesised according to literature procedures.[16,17]

4.3.1 Synthesis of PQS analogue 1

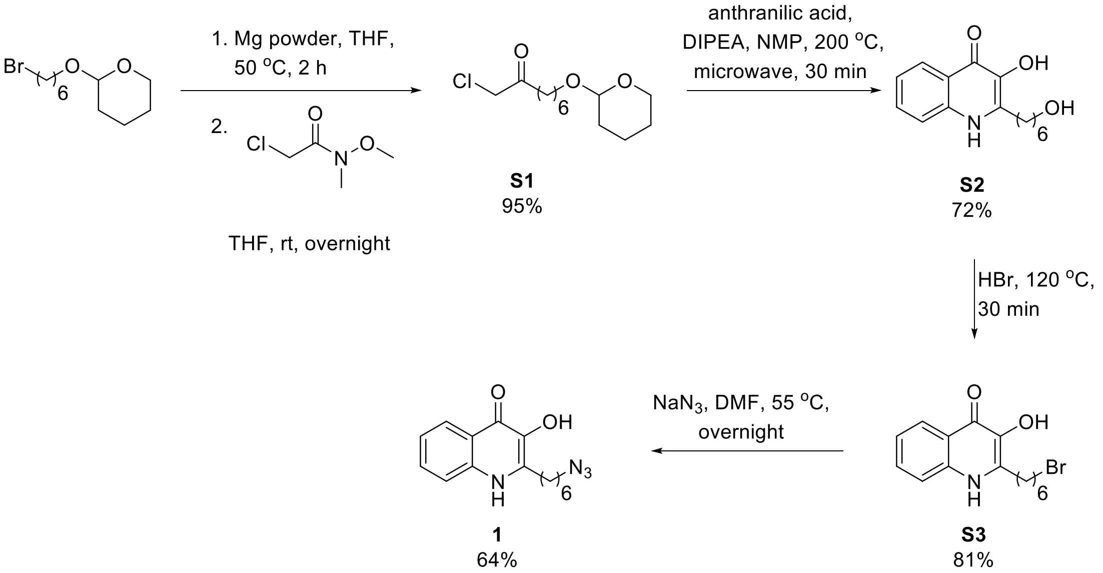

1-Chloro-8-((tetrahydro-2*H*-pyran-2-yl)oxy)octan-2-one S1

The title compound was prepared following the procedure outlined by Hodgkinson et al.[16] 2-((6-Bromohexyl)oxy)tetrahydro-2*H*-pyran (1.6 mL, 7.3 mmol, 1.0 eq) was added to a suspension of magnesium turnings (532 mg, 21.9 mmol, 3.0 eq) in THF (15 mL) under argon. The reaction was stirred at room temperature for 1 h, then heated to 50 °C for a further 2 h and cooled to room temperature to give a ~0.5 M solution of the Grignard reagent in THF. The Grignard solution was then added to a solution of 2-chloro-*N*-methoxy-*N*-methylacetamide (500 mg, 3.6 mmol, 0.5 eq) in THF (25 mL) and the mixture was stirred overnight at room temperature. The reaction was diluted two-fold with toluene, cooled to 0 °C and quenched with an equal volume of 0.1 M HCl. The organic phase was then washed with brine, dried over $MgSO_4$, filtered and the solvent removed under reduced pressure to give the resulting crude α-chloroketone **S1** as a pale yellow oil in 95% yield which was then used without further purification.

TLC (EtOAc:hexane, 4:6 v/v): R_f = 0.27; ^1H NMR (400 MHz, $CDCl_3$): δ 4.55 (dd, *J* = 4.6, 2.7 Hz, 1H), 4.06 (s, 2H), 3.85 (ddd, *J* = 11.1, 7.4, 3.2 Hz, 1H), 3.72 (dt, *J* = 9.6, 6.8 Hz, 1H), 3.54 – 3.42 (m, 1H), 3.37 (dt, *J* = 9.6, 6.5 Hz, 1H), 2.58 (t, *J* = 7.4 Hz, 2H), 1.82 – 1.44 (m, 10H), 1.44 – 1.28 (m, 4H); ^{13}C NMR (101 MHz, $CDCl_3$): δ 202.9, 99.0, 67.6, 62.5, 48.3, 39.8, 30.9, 29.7, 29.0, 26.1, 25.6, 23.7, 19.9; IR (neat)/cm^{-1}: 1727; HRMS (ESI-TOF) m/z: $[M + Na]^+$ calcd. for $C_{13}H_{23}O_3ClNa$, 285.1228; found, 285.1217.

3-Hydroxy-2-(6-hydroxyhexyl)quinolin-4(*1H*)-one S2

The title compound was prepared following the procedure outlined by Hodgkinson et al.[16] DIPEA (0.46 mL) and α-chloroketone **S1** (578 mg, 2.2 mmol, 1.0 eq), were added to a solution of anthranilic acid (300 mg, 2.2 mmol, 1.0 eq), in anhydrous NMP (4.5 mL). The mixture was then heated under microwave irradiation at 200 °C for 30 min. After cooling to room temperature the reaction was quenched by pouring into ice/water. The resulting mixture was stirred for 5 min before being allowed to settle for 30 min and the precipitate was collected and dried under vacuum to give the title compound **S2** (421 mg, 1.58 mmol) as a white precipitate in 72% yield.

Mp: 179.6-180.8 ˚C (EtOAc); TLC (MeOH:CH_2Cl_2, 1:9 v/v, KH_2PO_4 treated silica): R_f = 0.5; ^1H NMR (400 MHz, DMSO-d_6): δ 11.42 (s, 1H), 8.09 (d, *J* = 8.1 Hz, 1H), 7.53 (d, *J* = 3.9 Hz, 2H), 7.21 (dt, *J* = 8.1, 4.0 Hz, 1H), 4.33 (s, 1H), 3.37 (t, *J* = 6.4 Hz, 2H), 2.73 (t, *J* = 7.7 Hz, 2H), 1.66 (p, *J* = 7.3 Hz, 2H), 1.54 – 1.23 (m, 6H); ^{13}C NMR (101 MHz, DMSO-d_6): δ 168.9, 137.8, 137.4, 135.5, 124.5, 122.2, 121.5, 117.8, 60.7, 32.4, 28.8, 28.1, 27.9, 25.3; IR (neat)/cm^{-1}: 3227, 1628 , 1588, 1557 ; HRMS (ESI-TOF) m/z: $[M + H]^+$ calcd. for $C_{15}H_{19}NO_3$: 262.1438; found 262.1429;

2-(6-Bromohexyl)-3-hydroxyquinolin-4(1*H*)-one S3

Quinolone **S2** (15 mg, 0.057 mmol, 1.0 eq) was dissolved in 48% aqueous HBr (0.3 mL) and heated to 120 °C for 30 min with stirring. The reaction was cooled to room temperature and added slowly to a stirring saturated aqueous solution of $NaHCO_3$. The aqueous phase was extracted with EtOAc (3 x 10 mL), dried over $MgSO_4$, filtered and evaporated to dryness to give the title compound **S3** (15 mg, 0.046 mmol) as dark brown needles in 81% yield.

Mp: 157.3-158.5 ˚C (EtOAc); TLC (MeOH:CH_2Cl_2, 1:9 v/v, KH_2PO_4 treated silica): R_f: 0.59; ^1H NMR (500 MHz, DMSO-d_6): δ 11.44 (s, 1H), 8.09 (d, *J* = 8.1 Hz, 1H), 7.53 (d, *J* = 3.2 Hz, 2H), 7.21 (app dt, *J* = 8.1, 4.0 Hz, 1H), 3.52 (t, *J* = 6.7 Hz, 2H), 2.74 (t, *J* = 7.7 Hz, 2H), 1.80 (app p, *J* = 6.8 Hz, 2H), 1.72 – 1.63 (m, 2H), 1.48 – 1.31 (m, 4H); ^{13}C NMR (126 MHz, DMSO-d_6): δ 168.8, 137.8, 137.4, 135.4, 130.0, 124.5, 122.2, 121.5, 117.8, 35.1, 32.1, 28.0, 27.9, 27.7, 27.3; IR (neat)/cm^{-1}: 1638, 1599, 1557 , 757; HRMS (ESI-TOF) m/z: $[M + H]^+$ calcd. for $C_{15}H_{19}NO_2Br$: 324.0599; found 324.0600;

2-(6-Azidohexyl)-3-hydroxyquinolin-4(1H)-one 1

A solution of **S3** (10 mg, 0.03 mmol, 1.0 eq) and sodium azide (4 mg, 0.06 mmol, 2.0 eq) in DMF (1 mL) was heated to 55 °C overnight with stirring. The reaction was quenched by pouring into ice/water (20 mL) and the mixture was stirred for 5 min. The resulting precipitate was collected by filtration, washed with water and dried under vacuum. The crude produce was further purified by crystallisation from EtOAc to give the title compound **1** (5.7 mg, 0.02 mmol) as dark brown needles in 64% yield.

Mp: 167.2-168.1 ˚C (EtOAc); TLC (MeOH:CH_2Cl_2, 1:9 v/v, KH_2PO_4 treated silica): R_f = 0.41; ^{1}H NMR (400 MHz, DMSO-*d*$_6$): δ 11.43 (s, 1H), 8.09 (d, *J* = 8.1 Hz, 1H), 7.53 (app d, *J* = 4.2 Hz, 2H), 7.21 (app dt, *J* = 7.9, 3.8 Hz, 1H), 3.30 (t, *J* = 6.8 Hz, 2H), 2.73 (t, *J* = 7.6 Hz, 2H), 1.67 (t, *J* = 7.2 Hz, 2H), 1.53 (t, *J* = 6.8 Hz, 2H), 1.46 – 1.26 (m, 4H); ^{13}C NMR (101 MHz, DMSO-*d*$_6$) δ 168.9, 137.8, 137.4, 135.4, 130.0, 124.5, 122.2, 121.5, 117.8, 50.6, 28.3, 28.2, 28.0, 27.7, 25.9; IR (neat)/cm^{-1}: 3260, 2800 (br), 2093, 1640, 1597; HRMS (ESI-TOF) m/z: [M + H]$^+$ calcd. for $C_{15}H_{19}N_4O_2$: 287.1494; found 287.1503.

4.3.2 Synthesis of PQS analogue 2

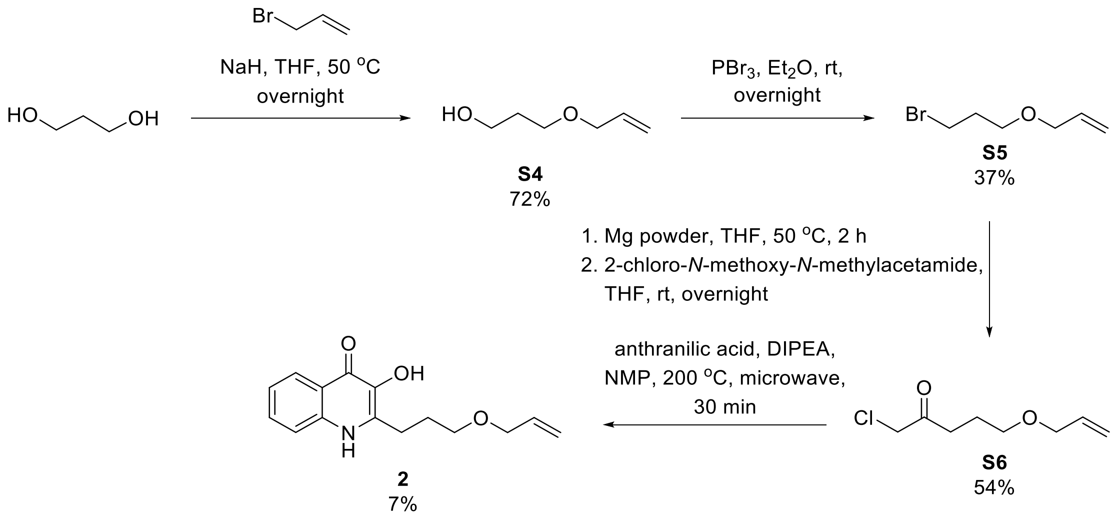

3-(Allyloxy)propan-1-ol S4

NaH (60% dispersion in mineral oil, 1.05 g, 26.3 mmol, 2.1 eq) was added piecewise to a solution of allyl bromide (2.17 mL, 12.8 mmol, 1.0 eq) and 1,3-propanediol (1.9 mL, 24 mmol, 1.9 eq) in anhydrous THF at 0 °C. The reaction was then heated at 50 °C overnight. The solvent was removed under vacuum and the residue was partioned between EtOAc (50 mL) and H_2O (50 mL). The organic layer was dried over $MgSO_4$, filtered and evaporated to dryness under reduced pressure. The crude product was purified by column chromatography (stepwise gradient of 20–40% Et_2O in 30-40 PE) to give the title compound **S4** (2.08g, 18 mmol) as a colourless oil in 72% yield.

TLC (Et_2O:40-60 PE, 4:6 v/v): R_f = 0.10; ^1H NMR (400 MHz, $CDCl_3$): δ 5.89 (ddt, *J* = 17.3, 10.4, 5.6 Hz, 1H), 5.26 (ddd, *J* = 17.2, 3.3, 1.4 Hz, 1H), 5.17 (ddd, *J* = 10.4, 2.9, 1.3 Hz, 1H), 3.98 (dt, *J* = 5.6, 1.4 Hz, 2H), 3.77 (t, *J* = 5.7 Hz, 2H), 3.62 (t, *J* = 5.8 Hz, 2H), 1.84 (tt, *J* = 5.8, 5.8 Hz, 2H); ^{13}C NMR (101 MHz, $CDCl_3$) δ 134.7, 117.2, 72.2, 69.5, 62.0, 32.2; IR (neat)/cm^{-1}: 3372 (br), 1647. Spectroscopic data consistent with literature values.[18]

3-(3-Bromopropoxy)prop-1-ene S5

Phosphorus tribromide (426 µL, 4.6 mmol, 0.5 eq) was added dropwise to a solution of compound **S4** (1.05 g, 9 mmol, 1.0 eq) in Et_2O (10 mL) at -5 °C. The reaction was allowed to warm to room temperature and left stirring overnight where it turned a yellow colour. The reaction was cooled to 0 °C, quenched by the addition of H_2O (15 mL) and diluted with hexane (30 mL). The organic phase was washed with water (2 x 30 mL), dried over Na_2SO_4, filtered and the solvent was removed under reduced pressure. The resulting oil was purified by column chromatography (0-5% Et_2O in hexane) to give the title compound **S5** (0.587 g, 3.3 mmol) as a colourless solution in ~37% yield. (Not all hexane was removed as product is volatile and the yield was calculated from ^1H NMR ratio of product:hexane).

TLC (Et_2O:hexane, 5:95 v/v): R_f = 0.30; ^1H NMR (400 MHz, $CDCl_3$): δ 5.91 (ddt, *J* = 17.3, 10.4, 5.6 Hz, 1H), 5.28 (ddd, *J* = 17.2, 3.3, 1.7 Hz, 1H), 5.19 (ddd, *J* = 10.3, 2.9 1.4 Hz, 1H), 3.98 (dt, *J* = 5.6, 1.4 Hz, 2H), 3.56 (t, *J* = 5.9 Hz, 2H), 3.52 (t, *J* = 6.5 Hz, 2H), 2.12 (tt, *J* = 6.3, 6.2 Hz, 2H); ^{13}C NMR (101 MHz, $CDCl_3$): δ 134.8, 117.1, 72.1, 67.8, 33.1, 31.7, 30.8. No spectroscopic data reported.[19]

5-(Allyloxy)-1-chloropentan-2-one S6

The title compound was prepared following the procedure outlined by Hodgkinson et al.[16] Alkyl bromide **S5** (250 mg, 1.4 mmol, 1.0 eq) was added to a suspension of magnesium turnings (50 mg, 4.2 mmol, 3.0 eq) in THF (3 mL) under argon. The reaction was stirred at room temperature for 1 h, then heated to 50 °C for a further 2 h and cooled to room temperature to give a ~0.5 M solution of the Grignard reagent in THF. The Grignard solution was then added to a solution of 2-chloro-*N*-methoxy-*N*-methylacetamide (96 mg, 0.7 mmol, 0.5 eq) in THF (5 mL) and the mixture was stirred overnight at room temperature. The reaction was diluted two-fold with toluene, cooled to 0 °C and quenched with an equal volume of 0.1 M HCl. The organic phase was then washed with brine, dried over $MgSO_4$, filtered and the solvent removed under reduced pressure to give a pale yellow oil. This was purified by column chromatography (stepwise gradient of 0-40% EtOAc in hexane) to afford **S6** (66 mg, 0.375 mmol) as a colourless oil in 54% yield. Contains the bromoketone as an impurity in the NMR ~25% Br ketone, 75% Cl ketone

TLC (EtOAc:hexane, 30:70 v/v): R_f = 0.30; ^1H NMR (400 MHz, $CDCl_3$): δ 5.88 (ddt, *J* = 17.3, 10.4, 5.6 Hz, 1H), 5.25 (dq, *J* = 17.2, 1.7 Hz, 1H), 5.17 (dq, *J* = 10.4, 1.4 Hz, 1H), 4.10 (s,

1.5H), 3.93 (dt, *J* = 5.6, 1.5 Hz, 2H), 3.91 (s, 0.5H (Br ketone)), 3.45 (td, *J* = 6.0, 1.0 Hz, 1.5H), 2.76 (t, *J* = 7.0 Hz, 0.5H (Br ketone)), 2.69 (t, *J* = 7.1 Hz, 2H), 1.96-1.89 (m, 2H); ^{13}C NMR (101 MHz, $CDCl_3$): δ 202.6, 134.8, 117.1, 71.9, 69.0, 48.4, 36.9 (Br ketone), 36.7, 34.5, (Br ketone) 24.4 (Br ketone), 24.1; $CDCl_3$ IR (neat)/cm^{-1}: 2934, 2858, 1716 , 1646.

2-(3-(Allyloxy)propyl)-3-hydroxyquinolin-4(1H)-one 2

The title compound was prepared following the procedure outlined by Hodgkinson et al.[16] DIPEA (194 µL, 1.1 mmol, 1.2 eq) and the required α-chloroketone **S6** (100 mg, 0.93 mmol, 1.0 eq) were added to a 0 solution of anthranilic acid (128 mg, 0.93 mmol, 1.0 eq) in anhydrous NMP (2 mL). The mixture was then heated under microwave irradiation at 200 °C for 30 min. After cooling to room temperature the reaction was quenched by pouring into ice/water. The resulting mixture was stirred for 5 min before being allowed to settle for 30 min and the precipitate was collected and dried under vacuum. The crude product was purified by column chromatography (stepwise gradient of 0-20% MeOH in CH_2Cl_2) to give **2** as an amorphous brown residue (17 mg, 0.066 mmol) in 7% yield.

TLC (MeOH:CH_2Cl_2, 7:93 v/v): R_f = 0.30 streaks; ^{1}H NMR (500 MHz, MeOD): δ 8.22 (dd, *J* = 8.2, 0.9 Hz,, 1H), 7.63 – 7.53 (m, 2H), 7.32 (ddd, *J* = 8.1, 6.4, 1.5 Hz, 1H), 5.89 (ddt, *J* = 17.3, 10.4, 5.6 Hz, 1H), 5.24 (ddd, *J* = 17.2, 3.4, 1.7 Hz, 1H), 5.12 (ddd, *J* = 10.4, 1.8, 1.3 Hz, 1H), 3.98 (dt, *J* = 5.6, 1.5 Hz, 2H), 3.55 (t, *J* = 6.2 Hz, 2H), 2.97 (t, *J* = 7.7 Hz, 2H), 2.08 – 2.02 (m, 2H); ^{13}C NMR (126 MHz, MeOD:) δ 170.6, 139.0, 138.8, 136.1, 131.7, 130.8. 125.5, 123.9, 123.7, 118.9, 117.2, 72.8, 70.5, 29.2, 27.0; IR (neat)/cm^{-1}: 3067, 1672, 1640, 1592, 1555; HRMS (ESI-TOF) m/z: $[M + H]^+$ calcd. for $C_{15}H_{18}NO_3$: 260.1281; found 260.1274.

4.3.3 Synthesis of HHQ analogue 3

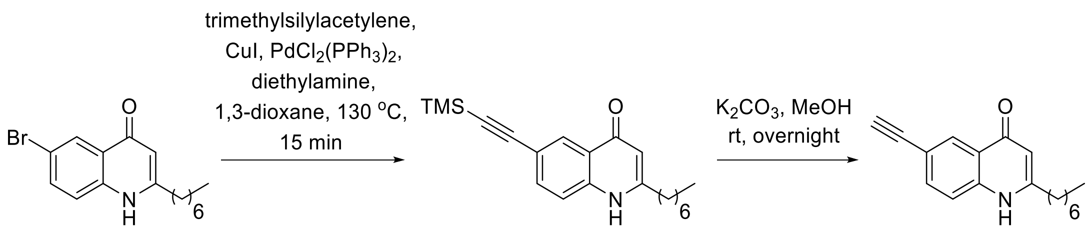

S7 **S8** 79% **3** 92%

2-Heptyl-6-((trimethylsilyl)ethynyl)quinolin-4(1H)-one S8

Based on a procedure by Mugnaini et al.[20] solution of **S7** prepared as previously reported[21] (128 mg, 0.4 mmol, 1.0 eq) in diisopropylamine (14 mL) and 1,3-dioxane (10 mL) was degassed by bubbling argon through the solution for 10 min before the addition of CuI (16 mg, 20 mol%), $PdCl_2(PPh_3)_2$ (28 mg, 10 mol%) and trimethylsilylacetylene (80 µL, 0.56 mmol, 1.8 eq). The mixture was heated in a sealed tube at 130 °C for 15 min after which the reaction

was diluted with CH_2Cl_2 (10 mL), filtered through celite and washed with 1 M HCl (10 mL) and brine (10 mL). The organic phase was dried over $MgSO_4$, filtered and evaporated to dryness under reduced pressure and the resulting residue purified by column chromatography (stepwise gradient of 0-4% MeOH in CH_2Cl_2) to give the title compound **S8** (108 mg, 0.32 mmol) as an off white amorphous powder in 79% yield (~95% pure by ^{1}H NMR).

TLC (MeOH:CH_2Cl_2, 5:95 v/v): R_f = 0.37; ^{1}H NMR (400 MHz, MeOD): δ 8.29 (d, *J* = 1.9 Hz, 1H), 7.72 (dd, *J* = 8.6, 1.9 Hz, 1H), 7.57 (d, *J* = 8.7 Hz, 1H), 6.25 (s, 1H), 2.74 (t, *J* = 7.7 Hz, 2H), 1.80 (app p, *J* = 7.6 Hz, 2H), 1.45 – 1.30 (m, 8H), 0.94 (t, *J* = 6.9 Hz, 3H), 0.29 (s, 9H); ^{13}C NMR (126 MHz, $CDCl_3$): δ 178.4, 154.8, 140.1, 134.8, 129.9, 124.77 118.6, 118.3, 108.9, 94.6, 51.02, 34.5, 31.8, 29.3, 29.1, 29.0, 22.7, 14.2, 0.1; IR (neat)/cm^{-1}: 3060, 2159, 1636, 1586, 1548, 836; HRMS (ESI-TOF) m/z: [M + H]$^+$ calcd. for $C_{21}H_{30}NOSi$, 340.2091; found, 340.2087.

6-Ethynyl-2-heptylquinolin-4(1H)-one 3

A solution of compound **S8** (40 mg, 0.12 mmol, 1.0 eq) and K_2CO_3 (120 mg, 0.89 mmol, 7.2 eq) in MeOH (4 mL) was stirred at room temperature overnight. The solvent was then removed under reduced pressure and the resulting mixture was partitioned between CH_2Cl_2 (10 mL) and H_2O (10 mL). The aqueous phase was extracted twice more with CH_2Cl_2 (10 mL) and the combined organic phases were dried over $MgSO_4$, filtered and the solvent removed under reduced pressure. The resulting residue was purified by column chromatography (stepwise gradient of 0-5% MeOH in CH_2Cl_2) to give the title compound **3** (29.6 mg, 0.1 mmol) in 92% yield. This was further purified by recrystallisation from CH_2Cl_2.

mp: 142.1-143.9 ˚C (CH_2Cl_2); TLC (MeOH:CH_2Cl_2, 5:95 v/v): R_f: 0.37; ^{1}H NMR (400 MHz, MeOD): δ 8.34 (d, *J* = 1.9 Hz, 1H), 7.76 (dd, *J* = 8.6, 1.9 Hz, 1H), 7.58 (dd, *J* = 8.6, 0.6 Hz, 1H), 6.26 (s, 1H), 3.60 (s, 1H), 2.75 (t, *J* = 7.7 Hz, 2H), 1.80 (app p, *J* = 7.6 Hz, 2H), 1.46 – 1.32 (m, 8H), 0.96 – 0.90 (m, 3H); ^{13}C NMR (126 MHz, $CDCl_3$): δ 178.3, 153.7, 139.8, 135.1, 130.4, 124.9, 117.8, 117.6, 109.5, 82.6, 77.2, 34.6, 31.8, 29.2, 29.1, 28.7, 22.7, 14.2; IR (neat)/cm^{-1}: 3056, 2924, 2576, 1636, 1586, 1548; HRMS (ESI-TOF) m/z: [M + H]$^+$ calcd. for $C_{18}H_{22}NO$, 268.1696; found, 268.1962.

4.3.4 Synthesis of HHQ analogue 4

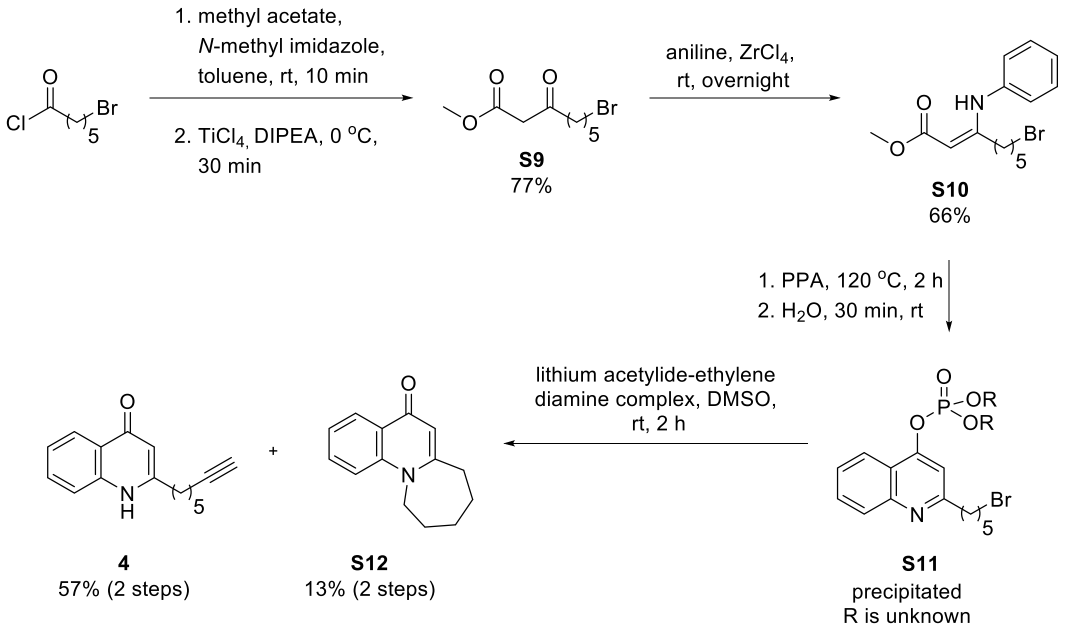

Methyl 8-bromo-3-oxooctanoate S9

Based on the reported procedure by Hashimoto et al.[22] 6-Bromohexanoyl chloride (15 g, 70 mmol, 1.0 eq) was added dropwise to a stirring solution of methyl acetate (7.62 mL, 96 mmol, 1.4 eq) and 1-methyl imidazole (5.74 mg, 72 mmol, 1.0 eq) in toluene (180 mL) forming a white precipitate. After 10 min the suspension was cooled to 0 °C before $TiCl_4$ (21.72 mL, 198 mmol, 2.8 eq) was added dropwise over 20 min with vigorous stirring and the mixture turned bright orange. DIPEA (37.6 mL, 216 mmol, 3.0 eq) was added dropwise over 20 min resulting in a dark red solution and the reaction was allowed to warm to room temperature over 30 min. The reaction was quenched by the addition of water (250 mL) and extracted with Et_2O (2 x 250 mL). The combined organic phases were dried over Na_2SO_4, filtered and evaporated to dryness yielding **S9** as a pale yellow oil (13.3 g, 53.6 mmol) in 77% yield without the need for purification.

TLC (Et_2O:40-60 PE, 3:7 v/v): R_f = 0.35; ^{1}H NMR (400 MHz, $CDCl_3$): δ 3.74 (s, 3H), 3.45 (s, 2H), 3.40 (t, *J* = 6.7 Hz, 3H), 2.57 (t, *J* = 7.2 Hz, 2H), 1.91 – 1.83 (m, 2H), 1.65-1.57 (m, 2H), 1.50 – 1.38 (m, 2H); ^{13}C NMR (101 MHz, $CDCl_3$): δ 202.7, 167.5, 124.8, 52.1, 48.8, 42.9, 28.8, 28.8, 23.3, 22.4; IR (neat)/cm^{-1}: 1735, 1623; HRMS (ESI-TOF) m/z: $[M + Na]^+$ calcd. for $C_9H_{15}OBrNa$, 273.0094; found, 273.0097.

Methyl (Z)-8-bromo-3-(phenylamino)oct-2-enoate S10

A mixture of *β*-keto ester **S9** (1.7 g, 6.9 mmol, 1.0 eq), aniline (632 µL, 6.9 mol, 1.0 eq) and $ZrCl_4$ (50 mg, 0.21 mmol, 3 mol%) was stirred at room temperature overnight. The reaction was diluted with Et_2O (20 mL), filtered and the organic phase washed with 0.1 M HCl (2 x 20

mL). The solvent was removed under reduced pressure and the resulting mixture was purified by column chromatography (stepwise gradient of 0-30% Et_2O in 40-60 PE) to give the title compound **S10** (1.13 g, 3.48 mmol) as an amorphous solid in 66% yield (96% based on recovered starting material).

TLC (Et_2O:40-60 PE, 3:7 v/v): R_f = 0.24; ^{1}H NMR (400 MHz, $CDCl_3$): δ 10.26 (s, 1H), 7.38 – 7.30 (m, 2H), 7.10 (t, *J* = 7.4 Hz, 1H), 7.09 (d, *J* = 7.5 H, 2H), 4.72 (s, 1H), 3.69 (s, 3H), 3.30 (t, *J* = 6.7 Hz, 2H), 2.31 (t, *J* = 7.5 Hz, 2H), 1.74 (app p, *J* = 6.9 Hz, 2H), 1.48 – 1.32 (m, 4H); ^{13}C NMR (101 MHz, $CDCl_3$): δ 171.1, 163.2, 139.3, 129.3, 125.6, 125.3, 84.9, 50.5, 33.5, 32.3, 32.2, 27.7, 27.2; IR (neat)/cm^{-1}: 1588, 1552, 760.

2-(Hept-6-yn-1-yl)quinolin-4(1H)-one 4 and 8,9,10,11-Tetrahydroazepino[1,2-a]quinolin-5(7H)-one S12

Imine **S11** (325 mg, 1.0 mmol, 1.0 eq) was added to a stirring solution of polyphosphoric acid (115% H_3PO_4 basis, 1.0 g) and heated to 90 °C with for 1 h. The reaction was cooled to room temperature and H_2O (20 mL) was then slowly added to the stirring mixture which was stirred for a further 30 min, and formed a white precipitate. The supernatant was then decanted and the precipitate was washed twice with H_2O and dried under vacuum to give the crude protected product as a white sticky solid. The precipitate was washed twice with water and dried under reduced pressure to give a gummy white solid which was then dissolved in DMSO (10 mL) and lithium acetylide-ethylenediamine complex (643 mg, 7.0 mmol, 7.0 eq) was added piecewise and stirred at room temperature overnight. The reaction was poured into an ice/brine mixture (1:1, 100 mL), acidified with 1 M HCl, extracted with CH_2Cl_2 (3 x 50 mL), dried over Na_2SO_4, filtered and evaporated to dryness. The resulting residue was purified by column chromatography (stepwise gradient of 0-5% MeOH in CH_2Cl_2) to give the title compound **4** (166 mg 0.57 mmol) as colourless needles in 57% yield over two steps. Side product **S12** (27.6 mg, 0.13 mmol) was isolated in 13% yield.

Data for 2-(Hept-6-yn-1-yl)quinolin-4(1*H*)-one 4

TLC (MeOH:CH_2Cl_2, 5:95 v/v): R_f = 0.43; ^{1}H NMR (400 MHz, $CDCl_3$): δ 11.23 (s, 1H), 8.35 (dd, *J* = 8.1, 1.4 Hz, 1H), 7.64 (dd, *J* = 8.4, 1.3 Hz, 1H), 7.59 (ddd, *J* = 8.4, 6.8, 1. Hz, 1H), 7.33 (ddd, *J* = 8.1, 6.8, 1.3 Hz, 1H), 6.22 (d, *J* = 1.5 Hz, 1H), 2.69 (t, *J* = 7.8 Hz, 2H), 2.12 (td, *J* = 6.7, 2.6 Hz, 2H), 1.90 (t, *J* = 2.6 Hz, 1H), 1.76 (app q, *J* = 7.7 Hz, 2H), 1.54 – 1.36 (m, 4H); ^{13}C NMR (101 MHz, $CDCl_3$): δ 179.1, 154.3, 140.5, 132.0, 125.6, 125.2, 123.8, 118.2, 108.6, 84.3, 68.7, 34.4, 28.5, 28.3, 28.1, 18.4; IR (neat)/cm^{-1}: 3261, 1666, 1629, 1595, 1519; HRMS (ESI-TOF) m/z: $[M + H]^+$ calcd. for $C_{16}H_{17}NO$, 240.1388; found, 240.1383.

Data for 8,9,10,11-Tetrahydroazepino[1,2-a]quinolin-5(7*H*)-one S12

mp: 99.9-101.3 ˚C ($CDCl_3$); TLC (MeOH:CH_2Cl_2, 5:95 v/v): R_f = 0.40; ^{1}H NMR (500 MHz,

$CDCl_3$): δ 8.46 (dd, *J* = 8.0, 1.7 Hz, 1H), 7.64 (ddd, *J* = 8.7, 7.0, 1.7 Hz, 1H), 7.52 (d, *J* = 8.6 Hz, 1H), 7.35 (ddd, *J* = 8.0, 6.9, 0.9 Hz, 1H), 6.24 (s, 1H), 4.35 – 4.23 (m, 2H), 2.91 (app dd, *J* = 7.3, 3.2 Hz, 2H), 1.91 – 1.81 (m, 6H); ^{13}C NMR (126 MHz, $CDCl_3$): δ 178.3, 157.0, 141.5, 132.2, 127.1, 126.8, 123.2, 115.0, 110.6, 47.3, 35.7, 28.5, 27.4, 26.7; IR (neat)/cm^{-1}: 1616, 2594, 1553; HRMS (ESI-TOF) m/z: $[M + H]^+$ calcd. for $C_{14}H_{16}NO$, 214.1226; found, 214.1223.

4.3.5 Synthesis of HHQ analogue 5

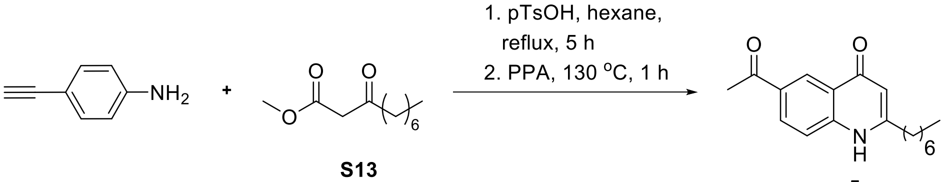

S13

5
27%

6-Acetyl-2-heptylquinolin-4(1H)-one 5

4-Ethynylaniline (146 mg, 1.24 mmol, 1.0 eq), methyl-3-oxydecanoate **S13** prepared following previously reported procedure[23] (250 mg, 1.25 mmol, 1.0 eq) and *p*-TsOH (5.5 mg, 2.5 mol%) were dissolved in hexane (2.5 mL) and heated to reflux with stirring using Dean-Stark apparatus for 5 h. The solvent was then removed under reduced pressure and the resulting imine was added to polyphosphoric acid (115% H_3PO_4 basis, 1.8 g) at 130 °C and stirred vigorously for 1 h. The resulting mixture was added slowly to a saturated solution of $NaHCO_3$ and stirred at 0 °C until a precipitate formed. The precipitate was collected by filtration and purified by column chromatography (stepwise gradient of 0-5% MeOH in CH_2Cl_2) to yield **5** (96 mg, 0.34 mmol) as a white amorphous solid in 27% yield.

mp: 206.5-208.5 ˚C (MeOH); TLC (MeOH:CH_2Cl_2, 5:95 v/v): R_f = 0.36; ^1H NMR (500 MHz, MeOD): δ 8.88 (d, *J* = 2.8 Hz, 1H), 8.33 (d, *J* = 8.7 Hz, 1H), 7.63 (dd, *J* = 9.2, 2.8 Hz, 1H), 6.27 (s, 1H), 2.73 (t, *J* = 7.9 Hz, 2H), 2.70 (s, 3H), 1.78 (app p, *J* = 7.5 Hz, 2H), 1.45 – 1.30 (m, 8H), 0.91 (t, *J* = 7.0 Hz, 3H); ^{13}C NMR (126 MHz, MeOD): δ 199.0, 180.7, 157.9, 144.4, 133.7, 131.8, 128.4, 124.7, 119.6, 110.0, 35.0, 32.9, 30.2, 30.1, 30.0, 26.5, 23.7, 14.4. IR (neat)/cm^{-1}: 3148, 3080, 1682, 1634, 1552; HRMS (ESI-TOF) m/z: $[M + H]^+$ calcd. for $C_{18}H_{24}NO_2$, 286.1802; found, 286.1793.

4.3.6 Synthesis of HHQ analogue 6

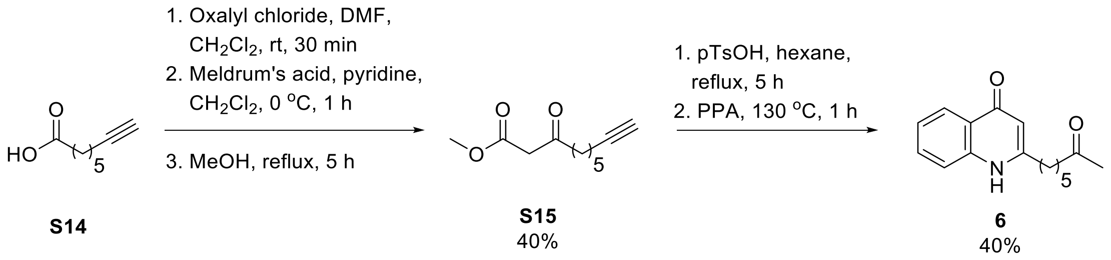

Methyl 3-oxodec-9-ynoate S15

The title compound was prepared based on a published procedure by Oikawa et al.[24] Oct-7-ynoic acid **S14** has was prepared as reported by Garner and Janda.[25] Oxalyl chloride (1 mL, 2 M in CH_2Cl_2, 2 mmol, 2.0 eq) was added dropwise to a solution of acid **S14** (140 mg, 1.0 mmol, 1.0 eq) and DMF (1 drop) in CH_2Cl_2 at 0 °C and the reaction was stirred for 1 h. Then the reaction was allowed to warm to room temperature and stirred for a further 2 h. After this the solvent was removed under reduced pressure and the acid chloride was dissolved in CH-$_2Cl_2$ (100 µL). A solution of Meldrum's acid (131 mg, 0.91 mmol, 1.0 eq) in dry CH_2Cl_2 (2 mL) was cooled to 0 °C and pyridine (150 µL) was added drop-wise over 20 min. The acid chloride in CH_2Cl_2 was then added and the mixture was stirred at 0 °C for a further 2 h. The reaction mixture was allowed to warm to room temperature, diluted with CH_2Cl_2 (x 2) and poured into an equal volume of cold HCl (2 N). The organic phase was washed with saturated NaCl, dried over $MgSO_4$, filtered and evaporated to dryness. The resulting orange/brown oil was heated to reflux in anhydrous MeOH (5 mL) for 5 h, evaporated to dryness and purified by (stepwise gradient of 5%-10% Et_2O in 40/60 PE) to give **S15** (78 mg, 0.4 mmol) as a colourless oil in 40% yield.

TLC (Et_2O:hexane, 2:8 v/v): R_f: 0.31; ^{1}H NMR (400 MHz, $CDCl_3$): δ 3.74 (s, 3H), 3.45 (s, 2H), 2.55 (t, *J* = 7.3 Hz, 2H), 2.19 (dt, *J* =7.3, 2.7 Hz, 2H), 1.94 (t, *J* = 2.7 Hz, 1H), 1.65 – 1.58 (m, 2H), 1.56 – 1.49 (m, 2H), 1.47 – 1.36 (m, 2H); ^{13}C NMR (101 MHz, $CDCl_3$) δ 202.6, 167.8, 84.4, 68.5, 52.5, 49.2, 43.0, 28.3, 28.2, 23.0, 18.4; IR (neat)/cm^{-1}: 3283, 1743. 1714; HRMS (ESI-TOF) m/z: $[M + H]^+$ calcd. for $C_{11}H_{17}O_3$, 197.1178, found: 197.1178.

Spectroscopic data consistent with literature values.[25]

2-(6-Oxoheptyl)quinolin-4(1H)-one 6

A solution of aniline (47.2 µL, 0.5 mmol, 1.0 eq), *p*-TsOH (2.42 mg, 2.5 mol%) and *β*-keto ester **S15** (100 mg, 0.5 mmol, 1.0 eq) in hexane (0.5 mL) was stirred at room temperature overnight. The solvent was removed under reduced pressure and the residue was added to polyphosphoric acid (115% H_3PO_4 basis, 1 g) heated to 120 °C with stirring for 1 h. The reaction was then allowed to cool to room temperature and added slowly to a saturated

solution of $NaHCO_3$ (15 mL) at 0 °C. The resulting precipitate was collected by filtration and purified by column chromatography (stepwise gradient of 0-5% MeOH in CH_2Cl_2) to yield **6** (90 mg, 0.23 mmol) as a white amorphous solid in 70% yield.

Mp: 123.4-124.5 ˚C ($CDCl_3$); TLC (MeOH:CH_2Cl_2, 5:95 v/v): R_f = 0.56; ^1H NMR (500 MHz, $CDCl_3$): δ 8.34 (d, *J* = 7.8 Hz, 1H), 7.60 – 7.51 (m, 2H), 7.32 (ddd, *J* = 8.1, 4.7, 3.4 Hz, 1H), 6.18 (d, *J* = 1.6 Hz, 1H), 2.67 (t, *J* = 7.4 Hz, 2H), 2.44 (t, *J* = 6.8 Hz, 2H), 2.14 (s, 3H), 1.72 (app p, *J* = 7.3 Hz, 2H), 1.65 – 1.52 (m, 2H), 1.33 (tt, *J* = 10.5, 6.4 Hz, 2H); ^{13}C NMR (126 MHz, $CDCl_3$): δ 209.8, 179.1, 153.6, 140.2, 131.9, 125.9, 125.2, 123.7, 117.9, 109.0, 43.3, 33.7, 30.3, 28.7, 28.1, 22.5; IR (neat)/cm^{-1}: 1705, 1640, 1594; HRMS (ESI-TOF) m/z: $[M + H]^+$ calcd. $C_{16}H_{20}NO_2$: 258.1489; found: 258.1483.

4.3.7 Synthesis of PQS analogue 7

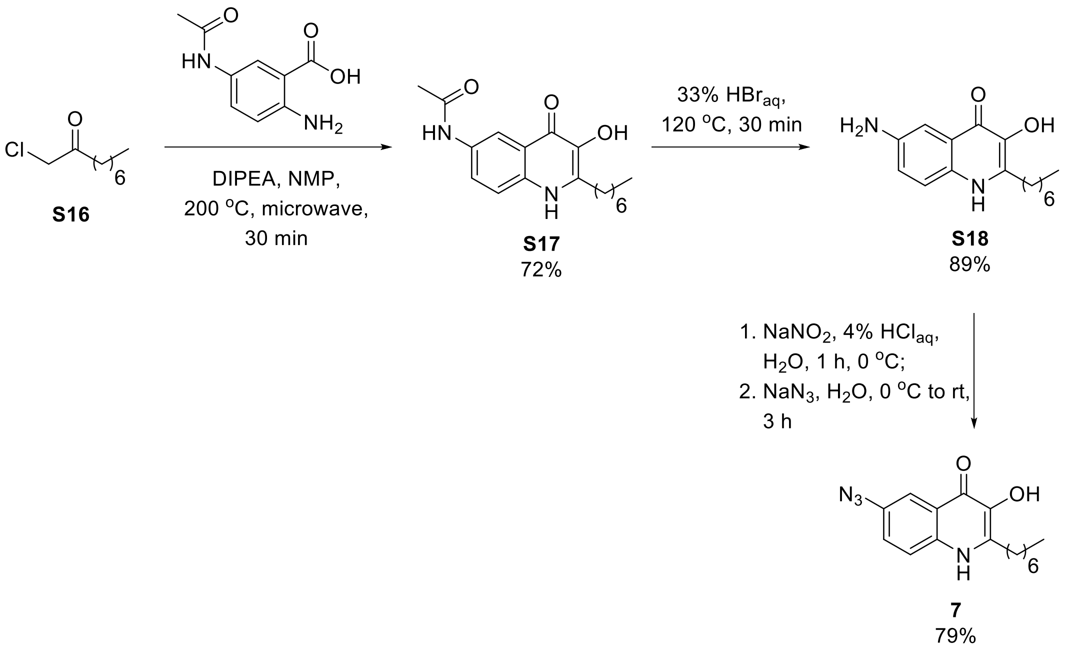

N-(2-heptyl-3-hydroxy-4-oxo-1,4-dihydroquinolin-6-yl)acetamide S17

The title compound was prepared following the procedure outlined by Hodgkinson et al[16] DIPEA (0.46 mL, 2.6 mmol, 1.2 eq) and the required α-chloroketone **S16** prepared according as previously reported[16] (578 mg, 2.2 mmol, 1.0 eq), were added to a solution of 5-(acetylamino)-2-aminobenzoic acid (427 mg, 2.2 mmol, 1.0 eq), in anhydrous NMP (4.5 mL). The mixture was then heated under microwave irradiation at 200 °C for 30 min. After cooling to room temperature the reaction was quenched by pouring into ice/water. The resulting mixture was stirred for 5 min before being allowed to settle for 30 min and the precipitate was collected and dried under vacuum to give **S17** (421 mg, 1.58 mmol) as a beige amorphous solid in 72% yield.

mp: degrades ~300 ˚C (EtOAc); TLC (MeOH:CH_2Cl_2, 1:9 v/v, KH_2PO_4 treated silica): R_f = 0.5; ^{1}H NMR (500 MHz, DMSO-d_6): δ 11.39 (s, 1H), 10.04 (s, 1H), 8.32 (d, J = 2.4 Hz, 1H), 7.74 (dd, J = 9.0, 2.5 Hz, 1H), 7.48 (d, J = 9.0 Hz, 1H), 2.71 (t, J = 7.7 Hz, 1H), 2.06 (s, 3H), 1.75 – 1.56 (m, 2H), 1.37 – 1.16 (m, 8H), 0.84 (t, J = 6.8 Hz, 3H); ^{13}C NMR (126 MHz, DMSO-d_6): δ 168.4, 168.1, 137.4, 135.2, 133.7, 133.6, 123.1, 122.4, 118.2, 112.7, 31.2, 28.8, 28.5, 28.1, 27.8, 24.0, 22.1, 13.9; IR (neat)/cm^{-1}: 3397, 3284, 3144, 1658, 1645, 1536; HRMS (ESI-TOF) m/z: [M + H]$^+$ calcd. for $C_{18}H_{25}N_2O$: 317.1860; found 317.1856.

6-Amino-2-heptyl-3-hydroxyquinolin-4(1H)-one S18

Intermediate **S16** (400 mg, 1.3 mmol, 1.0 eq) was dissolved in HBr (33% aqueous solution, 4 mL) and heated to 120 °C for 30 min with stirring. The reaction was cooled to room temperature and a crystalline solid formed. The solid was collected by filtration and partitioned between EtOAc/$NaHCO_3$. The organic layer was dried over Na_2SO_4, filtered and the solvent removed under reduced pressure to give **S18** (308 mg, 1.12 mmol) as an amorphous solid in 89% yield.

mp: decomposes ~360 ˚C (EtOAc); TLC (MeOH:CH_2Cl_2, 1:9 v/v, KH_2PO_4 treated silica): R_f = 0.45; ^{1}H NMR (400 MHz, MeOD): δ 8.15 (d, J = 2.3 Hz, 1H), 8.04 (d, J = 9.1 Hz, 1H), 7.76 (dd, J = 9.1, 2.4 Hz, 1H), 3.14 (t, J = 8.0 Hz, 2H), 1.83 (app p, J = 7.6 Hz, 1H), 1.53 – 1.26 (m, 9H), 0.95 – 0.86 (t, J = 6.9 Hz, 3H); ^{13}C NMR (126 MHz, MeOD) δ 159.9, 152.6, 137.6, 135.4, 134.4, 127.5, 122.4, 121.9, 115.1, 32.8, 31.0, 30.5, 30.0, 29.4, 23.7, 14.4; IR (neat)/cm^{-1}: 3000, 1607, 1556; HRMS (ESI-TOF) m/z: [M + H]$^+$ calcd. for $C_{16}H_{23}N_2O_2$: 275.1767; found 275.1760.

6-Azido-2-heptyl-3-hydroxyquinolin-4(1H)-one 7

Compound **S18** (274 mg, 1 mmol, 1.0 eq) was dissolved in 4% HCl (5 mL) and the solution was cooled to 0 °C. A solution of $NaNO_2$ (140 mg, 3 mmol, 2.0 eq) in H_2O (1 mL) was added dropwise and the reaction stirred at 0 °C for 1 h. A solution of NaN_3 (163 mg, 2.5 mmol, 2.5 eq) in H_2O, (1 mL) was then added dropwise and the reaction was allowed to warm to room temperature and stirred for a further 3 h. The reaction was diluted with H_2O (10 mL) and extracted with EtOAc (3 x 20 mL). The combined organic extracts dried over $MgSO_4$, filtered and evaporated to dryness to give **7** (211 mg, 0.70 mmol) as brown crystals in 70% yield.

mp: decomposes ~136.7 ˚C (CH_2Cl_2); TLC (MeOH:CH_2Cl_2, 1:9 v/v, KH_2PO_4 treated silica): R_f = 0.67; ^{1}H NMR (400 MHz, DMSO-d_6): δ 12.06 (s, 1H), 7.74 (d, J = 2.6 Hz, 1H), 7.67 (d, J = 8.9 Hz, 1H), 7.33 (dd, J = 8.9, 2.7 Hz, 1H), 2.77 (t, J = 7.7 Hz, 2H), 1.67 (app p, J = 7.3 Hz, 2H), 1.35 – 1.21 (m, 8H), 0.85 (t, J = 6.7 Hz, 3H); ^{13}C NMR (101 MHz, DMSO-d_6): δ 166.2, 137.9, 137.8, 134.5, 133.9, 122.7, 122.6, 120.4, 112.4, 31.2, 28.8, 28.4, 28.3, 27.8, 22.1, 14.0; IR (neat)/cm^{-1}: 3089, 2108, 1632, 1591, 1551; HRMS (ESI-TOF) m/z: [M + H]$^+$ calcd. for $C_{16}H_{21}N_4O_2$: 301.1659; found: 301.1649.

4.3.8 Synthesis of HHQ analogue 8

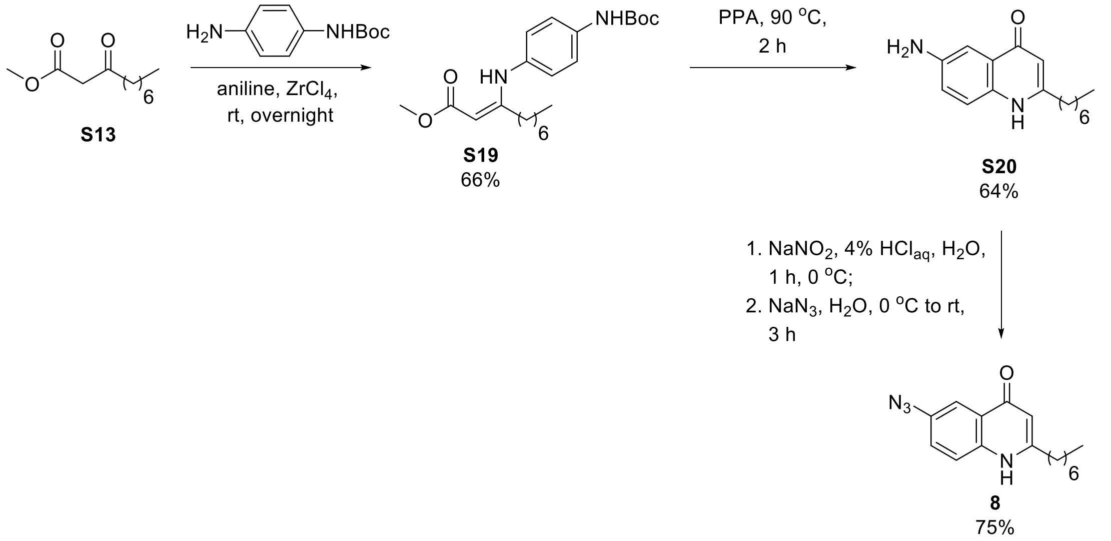

Methyl (Z)-8-bromo-3-((4-((tert-butoxycarbonyl)amino)phenyl)amino)oct-2-enoate S19

A mixture of *β*-keto ester **S13**[23] (2.0 g, 8 mmol, 1.0 eq), *N*-Boc-*p*-phenylenediamine (1.6 g, 8 mmol, 1.0 eq) and $ZrCl_4$ (60 mg, 0.26 mmol, 3 mol%) was stirred at room temperature overnight. The reaction was diluted with Et_2O (20 mL), filtered and the solvent was removed under reduced pressure. The crude product was purified by column chromatography (stepwise gradient of 0-40% Et_2O in 40-60 PE) to give the title compound **S19** (1.7 g, 4.4 mmol) as a pale yellow-white amorphous solid in 66% yield (98% based on recovered starting material).

TLC (Et_2O:hexane, 3:7 v/v): R_f = 0.45; ^{1}H NMR (500 MHz, $CDCl_3$): δ 10.15 (s, 1H), 7.33 (d, *J* = 8.5 Hz, 2H), 7.02 (d, *J* = 8.7 Hz, 2H), 6.48 (s, 1H), 4.69 (s, 1H), 3.68 (s, 3H), 2.21 (t, *J* = 7.8 Hz, 2H), 1.52 (s, 9H), 1.41 – 1.14 (m, 10H), 0.84 (t, *J* = 7.1 Hz, 3H); ^{13}C NMR (126 MHz, CDCl: δ 171.2, 164.4, 152.8, 136.1, 134.3, 126.4, 119.2, 84.0, 80.9, 50.4, 32.3, 31.7, 29.2, 29.0, 28.5, 28.1, 22.7, 14.2; IR (neat)/cm^{-1}: 3340, 3259, 1724, 1634, 1610, 1583, 1519; HRMS (ESI-TOF) m/z: $[M + H]^+$ calcd. for $C_{22}H_{35}N_2O_4$: 391.2587; found: 391.2587.

6-Amino-2-heptylquinolin-4(1H)-one S20

Enamine **S19** (195 mg, 0.50 mmol, 1.0 eq) was added slowly to polyphosphoric acid (115% H_3PO_4 basis, 2.0 g) at 90 °C and stirred for 2 h. The mixture was then added slowly to a stirring solution of saturated $NaHCO_3$ and, once the bubbling had stopped, extracted with CH_2Cl_2 to yield the target compound **S20** (81.5 mg, 0.32 mmol) in 64% yield as an amorphous brown solid without need for further purification.

TLC (MeOH:CH_2Cl_2, 5:95 v/v): R_f = 0.39; ^{1}H NMR (400 MHz, DMSO-d_6): δ 7.26 (d, *J* = 8.7 Hz, 1H), 7.14 (d, *J* = 2.5 Hz, 1H), 6.94 (dd, *J* = 8.7, 2.5 Hz, 1H), 5.73 (s, 1H), 5.14 (s, 2H),

1.63 (app p, *J* = 7.4 Hz, 2H), 1.31-1.23 (m, 8H), 0.85(t, *J* = 6.9 Hz, 3H); ^{13}C NMR (101 MHz, DMSO-d_6): δ 176.3, 151.3, 144.7, 132.0, 126.2, 120.7, 118.6, 105.7, 105.4, 33.2, 31.2, 28.6, 28.5, 28.4, 22.1, 14.0; IR (neat)/cm^{-1}: 3340, 3224, 1659, 1623, 1506; LRMS (ESI-TOF) m/z: $[M + H]^+$ calcd. for $C_{16}H_{23}N_2O_2$: 258.2; found: 258.4.

6-Azido-2-heptylquinolin-4(1H)-one 8

Compound **S20** (258 mg, 1 mmol, 1.0 eq) was dissolved in 4% HCl (5 mL) and the solution was cooled to 0 °C. A solution of $NaNO_2$ (140 mg, 3 mmol, 2.0 eq) in H_2O (1 mL) was added dropwise and the reaction stirred at 0 °C for 1 h. A solution of NaN_3 (163 mg, 2.5 mmol, 2.5 eq) in H_2O, (1 mL) was then added dropwise and the reaction was allowed to warm to room temperature and stirred for a further 3 h. The reaction was diluted with H_2O (10 mL) and extracted with EtOAc (3 x 20 mL) The combined organic extracts dried over $MgSO_4$, filtered and evaporated to dryness to give **8** (213 mg, 0.75 mmol) as orange brown crystals in 75% yield.

Mp: decomposes 172.6-177.4 ˚C (EtOAc); TLC (MeOH:CH_2Cl_2, 5:95 v/v): R_f = 0.42; ^{1}H NMR (500 MHz, MeOD): δ 7.89 (d, *J* = 2.6 Hz, 1H), 7.62 (d, *J* = 8.9 Hz, 1H), 7.38 (dd, *J* = 8.9, 2.6 Hz, 1H), 6.23 (s, 1H), 2.72 (t, *J* = 7.7 Hz, 2H), 1.76 (app p, *J* = 7.7 Hz, 2H), 1.46 – 1.26 (m, 8H), 0.90 (t, *J* = 7.7 Hz, 3H); ^{13}C NMR (126 MHz, MeOD): δ 179.5, 157.1, 138.9, 138.0, 126.5, 125.6, 121.3, 114.4, 108.8, 35.0, 32.9, 30.2, 30.2, 30.1, 23.7, 14.4; IR (neat)/cm^{-1}: 2108, 1592, 1551, 1505; HRMS (ESI-TOF) m/z: $[M + H]^+$ calcd. for $C_{16}H_{20}N_3O$: 285.1715; found: 285.1721.

4.3.9 Synthesis of HHQ analogue 9

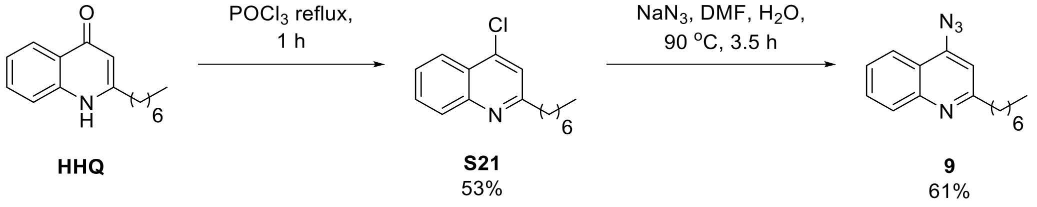

4-Chloro-2-heptylquinoline S21

HHQ prepared following a literature procedure[17] (14 mg, 0.058 mmol, 1.0 eq) was dissolved in $POCl_3$ (1 mL) and the mixture was heated to reflux for 1 h. The excess $POCl_3$ was removed under reduced pressure and the resulting residue was dissolved in CH_2Cl_2 (5 mL) and washed with saturated $NaHCO_3$ (3 x 5 mL). The organic layer was then dried over $MgSO_4$, filtered and evaporated to dryness to give **S21** (10 mg, 0.3 mmol) as a colourless oil in 53% yield without further purification.

TLC (Et_2O:hexane, 2:8 v/v): R_f = 0.58; ^{1}H NMR (500 MHz, $CDCl_3$): δ 8.18 (ddd, *J* = 8.4, 1.6, 0.6 Hz, 1H), 8.05 (ddd, *J* = 8.5, 1.6, 0.9 Hz, 1H), 7.73 (ddd, *J* = 8.4, 6.9, 1.4 Hz, 1H), 7.58

(ddd, *J* = 8.2, 6.9, 1.2 Hz, 1H), 7.40 (s, 1H), 2.94 (t, *J* = 7.9 Hz, 2H), 1.84 – 1.77 (m, 2H), 1.44 – 1.25 (m, 8H), 0.88 (t, *J* = 7.0 Hz, 3H); ^{13}C NMR (126 MHz, $CDCl_3$): δ 163.2, 148.9, 142.6, 130.4, 129.3, 126.8, 125.1, 124.1, 121.5, 39.3, 31.9, 30.0, 29.6, 29.3, 22.8, 14.2; IR (neat)/cm^{-1}: 1616, 1589, 1552, 757;HRMS (ESI-TOF) m/z: $[M + H]^+$ calcd. for $C_{16}H_{21}NCl$: 262.1363; found: 262.1367.

4-Azido-2-heptylquinoline 9

Compound **S21** (40 mg, 0.15 mmol, 1.0 eq) and NaN_3 (52.5 mg, 0.8 mmol, 5.3 eq) were dissolved in a mixture of DMF and H_2O (8:3, 1 mL) and heated to 90 ˚C for 4 h. The reaction was cooled to rt, diluted with saturated NH_4Cl (30 mL) and extracted with CH_2Cl_2 (3 x 30 mL). The combined organic phases were dried over $MgSO_4$, filtered and the solvent was removed under reduced pressure. The crude product was triturated with Et_2O, filtered and the solvent removed under vacuum to give **9** (24.5 mg, 0.09 mmol) as a waxy amorphous solid in 61% yield.

TLC (Et_2O:hexane, 2:8 v/v): R_f = 0.51; ^{1}H NMR (500 MHz, MeOD): δ 8.07 (dd, *J* = 8.4, 1.4 Hz, 1H), 7.96 (dt, *J* = 8.6, 0.8 Hz, 1H), 7.97 – 7.95 (m, 1H), 7.54 (ddd, *J* = 8.2, 6.8, 1.2 Hz, 1H), 7.29 (s, 1H), 2.97 (t, *J* = 7.9 Hz, 2H), 1.85 – 1.80 (m, 2H), 1.47 – 1.28 (m, 8H), 0.91 (t, *J* = 6.3 Hz, 3H); ^{13}C NMR (126 MHz, MeOD) δ 165.1, 149.4, 148.6, 131.9, 128.3, 127.1, 123.3, 121.5, 110.2, 39.7, 32.9, 31.3, 30.3, 23.7, 14.4. IR (neat)/cm^{-1}: 2109, 1619, 1590, 1559, 1502; HRMS (ESI-TOF) m/z: $[M + H]^+$ calcd. for $C_{16}H_{21}N_4$: 269.1766; found: 269.1763.

4.3.10 Synthesis of PQS probe 10

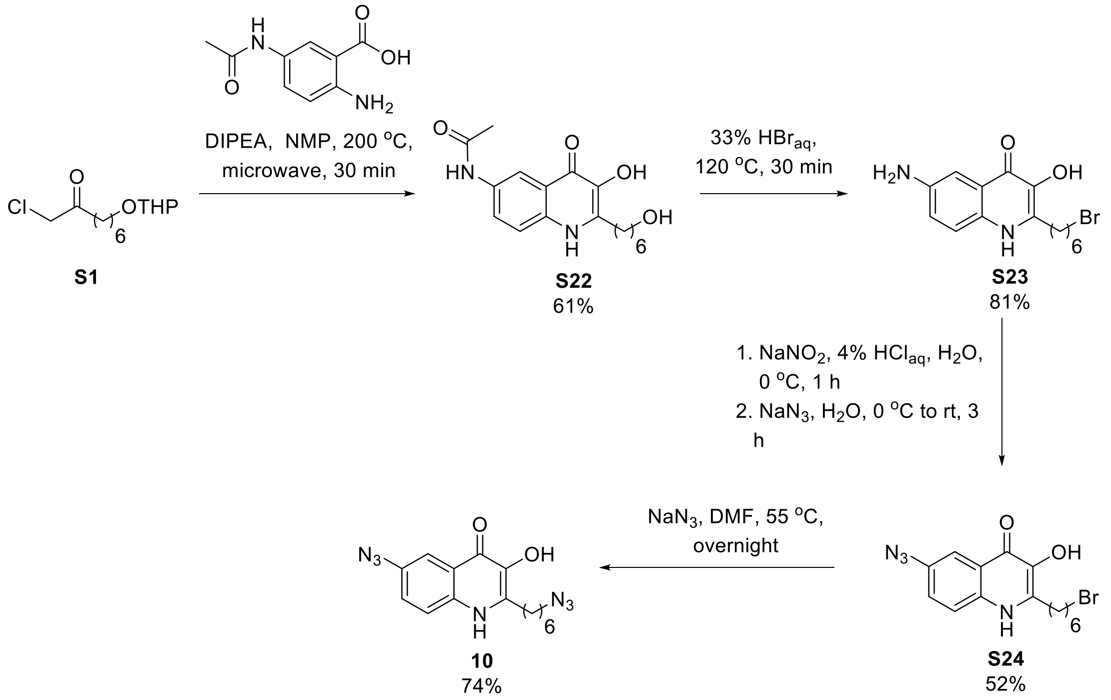

N-(3-hydroxy-2-(6-hydroxyhexyl)-4-oxo-1,4-dihydroquinolin-6-yl)acetamide S22

The title compound was prepared following the procedure outlined by Hodgkinson et al.[16] DIPEA (0.23 mL, 1.3 mmol, 1.2 eq) and α-chloroketone **S1** (282 mg, 1.1 mmol, 1.0 eq) were added to a solution of acetamidoanthranilic acid (212 mg, 1.1 mmol, 1.0 eq) in anhydrous NMP (2.25 mL). The mixture was then heated under microwave irradiation at 200 °C for 30 min. After cooling to room temperature the reaction was quenched by pouring into acetonitrile (20 mL) to give **S22** (214 mg, 0.67 mmol) in 61% yield as a pale yellow powder which was used without further purification.

mp: 276.3-277.8 ˚C (EtOAc); TLC (MeOH:CH_2Cl_2, 1:9 v/v, KH_2PO_4 treated silica): R_f = 0.27; ^{1}H NMR (500 MHz, DMSO-d_6): δ 11.40 (s, 1H), 10.04 (s, 1H), 8.32 (d, J = 2.4 Hz, 1H), 7.74 (dd, J = 9.0, 2.4 Hz, 1H), 7.48 (d, J = 9.0 Hz, 1H), 4.33 (s, 1H), 3.36 (t, J = 6.5 Hz, 2H), 2.71 (t, J = 7.7 Hz, 2H), 2.06 (s, 3H), 1.65 (app q, J = 7.7 Hz, 2H), 1.44 – 1.38 (m, 2H), 1.37 – 1.30 (m, 4H); ^{13}C NMR (126 MHz, DMSO-d_6): δ 168.4, 168.1, 137.4, 135.2, 133.7, 133.6, 123.1, 122.4, 118.2, 112.7, 60.6, 32.4, 28.8, 28.1, 27.9, 27.8, 25.3, 24.0; IR (neat)/cm^{-1}: 3269, 3127, 3100, 1661, 1643, 1613, 1539; HRMS (ESI-TOF) m/z: [M + H]$^+$ calcd. for $C_{17}H_{23}N_2O_4$: 319.1658; found 319.1661.

6-Amino-2-(6-bromohexyl)-3-hydroxyquinolin-4(1H)-one S23

Compound **S22** (738 mg, 2.46 mmol, 1.0 eq) was dissolved in HBr (33% aqueous solution, 2.4 mL) and heated to 120 °C for 30 min with stirring. The reaction was cooled to room temperature and a crystalline solid was formed. The solid was collected by filtration and partitioned between EtOAc/$NaHCO_3$. The organic layer was dried over Na_2SO_4, filtered and the solvent removed under reduced pressure to give **S23** (840 mg, 2.0 mmol) as an amorphous solid in 81% yield.

TLC (MeOH:CH_2Cl_2, 1:9 v/v, KH_2PO_4 treated silica): R_f = 0.43; ^{1}H NMR (400 MHz, DMSO-d_6): δ 7.38 (d, J = 8.8 Hz, 1H), 7.24 (d, J = 2.5 Hz, 1H), 7.02 (dd, J = 8.8, 2.5 Hz, 1H), 3.51 (t, J = 6.7 Hz, 2H), 2.72 (t, J = 7.8 Hz, 2H), 1.79 (app p, J = 6.8 Hz, 2H), 1.69 – 1.61 (m, 2H), 1.46-1.30 (m, 4H); ^{13}C NMR (101 MHz, DMSO-d_6): δ 165.7, 142.8, 136.5, 135.7, 130.4, 123.3, 121.4, 118.9, 104.5, 35.2, 32.1, 28.1, 27.9, 27.8, 27.3; IR (neat)/cm^{-1}: 2800 (Br), 1605, 1557; HRMS (ESI-TOF) m/z: [M + H]$^+$ calcd. for $C_{15}H_{20}N_2O_2Br$: 339.0708; found: 339.0708.

6-Azido-2-(5-bromopentyl)-3-hydroxyquinolin-4(1H)-one S24

A solution of $NaNO_2$ (31 mg, 0.45 mmol, 1.3 eq) in water (1 mL) was added dropwise to a stirring solution of compound **S23** (100 mg, 0.31 mmol, 1.0 eq) in a 4% aqueous solution of HCl (3 mL) at 0 °C. A solution of NaN_3 (42 mg, 0.649 mmol, 2.1 eq) in water (1 mL) was then added dropwise, the mixture allowed to warm to room temperature and left to stir for 3 h. The

pH of the reaction was adjusted to pH 10-12 with 10% NaOH and the aqueous layer was extracted with EtOAc (3 x 10 mL). The combined organic phases washed with brine (20 mL), dried over Na_2SO_4, filtered and evaporated to dryness to give the title compound S24 (84 mg, 0.24 mmol) as pale brown crystals in 52% yield.

mp: decomposes ~72.5 ˚C (EtOAc); TLC (MeOH:CH_2Cl_2, 1:9 v/v, KH_2PO_4 treated silica): R_f =0.47; ^{1}H NMR (500 MHz, DMSO-d_6): δ 11.59 (s, 1H), 7.72 (d, *J* = 2.7 Hz, 1H), 7.60 (d, *J* = 9.0 Hz, 1H), 7.29 (dd, *J* = 8.9, 2.7 Hz, 1H), 3.52 (t, *J* = 6.7 Hz, 2H), 2.73 (t, *J* = 7.7 Hz, 2H), 1.80 (app p, *J* = 6.8 Hz, 2H), 1.70-1.64 (m, 2H), 1.47 – 1.34 (m, 4H); ^{13}C NMR (126 MHz, DMSO-d_6) δ 167.8, 138.0, 135.9, 134.8, 133.2, 122.9, 122.5, 120.2, 112.6, 35.1, 32.1, 28.0, 27.9, 27.6, 27.3; IR (neat)/cm^{-1}: 2116, 1630, 1590, 1552, 815; HRMS (ESI-TOF) m/z: $[M + H]^+$ calcd. for $C_{15}H_{18}BrN_4O_2$: 365.0608; found: 365.0594.

6-Azido-2-(6-azidohexyl)-3-hydroxyquinolin-4(1H)-one 10

A solution of compound **S24** (50 mg, 0.14 mmol, 1.0 eq) and sodium azide (16.8 mg, 0.28 mmol, 2.0 eq) in DMF (1 mL) was stirred overnight at room temperature. The reaction was quenched by pouring into ice/water (20 mL) and the mixture was stirred for 5 min. The resulting precipitate was collected by filtration, washed with water and dried under vacuum. The crude product was further purified by crystallisation from EtOAc to give the title compound **10** (34.5 mg, 0.11 mmol) as an amorphous dark brown solid in 74% yield.

TLC (MeOH:CH_2Cl_2, 1:9 v/v, KH_2PO_4 treated silica): R_f =0.67; ^{1}H NMR (400 MHz, DMSO-d_6): δ 11.58 (s, 1H), 8.20 (s, 1H), 7.72 (d, *J* = 2.6 Hz, 1H), 7.60 (d, *J* = 8.9 Hz, 1H), 7.29 (dd, *J* = 8.9, 2.6 Hz, 1H), 3.31 (t, *J* = 6.5 Hz, 3H, partially obscured by H_2O peak), 2.73 (t, *J* = 7.7 Hz, 2H), 1.79 – 1.63 (m, 2H), 1.60 – 1.49 (m, 4H), 1.37 (t, *J* = 3.8 Hz, 2H); ^{13}C NMR (126 MHz, DMSO-d_6): δ 167.8, 138.0, 135.9, 134.8, 133.2, 129.6, 122.9, 122.4, 120.2, 112.6, 50.6, 28.3, 28.1, 28.0, 25.9; IR (neat)/cm^{-1}: 2119, 2097, 1717, 1519; HRMS (ESI-TOF) m/z: $[M + H]^+$ calcd. for $C_{15}H_{18}N_7O_2$: 328.1516; found: 328.1503.

4.3.11 Synthesis of HHQ probe 11

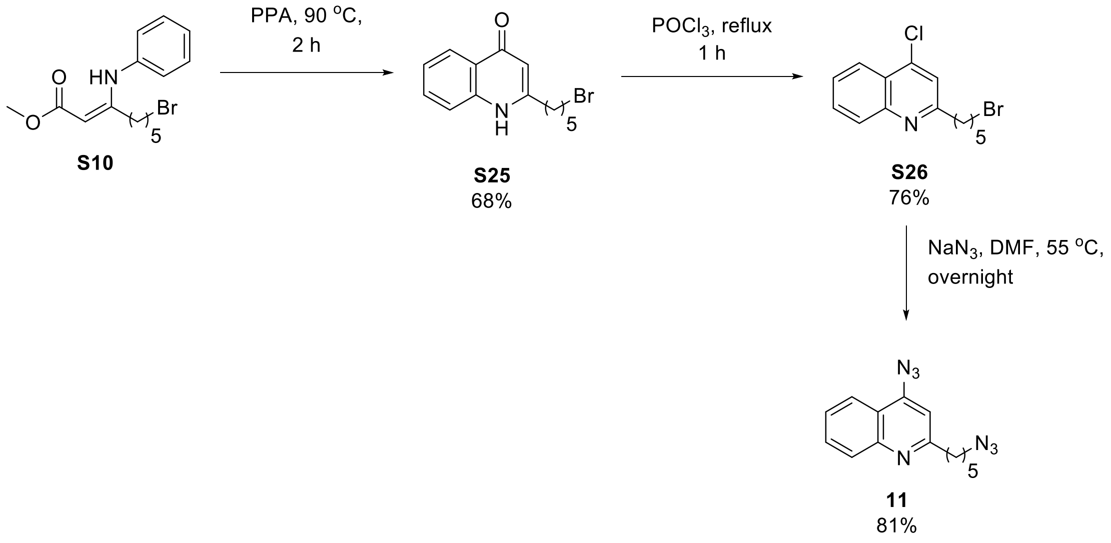

2-(5-Bromopentyl)quinolin-4(1H)-one S25

Enamine **S10** (325 mg, 1.0 mmol, 1.0 eq) was added to a stirring solution of polyphosphoric acid (115% H_3PO_4 basis, 1.0 g) and heated to 90 °C for 1 h. The reaction was then added slowly to a saturated solution of $NaHCO_3$ (15 mL) at 0 °C. The resulting precipitate was collected by filtration and purified by column chromatography (stepwise gradient of 0-5% MeOH in CH_2Cl_2) to yield **S11** (199 mg, 0.68 mmol) as a white amorphous solid in 68% yield.

mp: 128.4-129.9 ˚C (CH_2Cl_2/MeOH); TLC (MeOH:CH_2Cl_2, 5:95 v/v): R_f = 0.46; ^{1}H NMR (400 MHz, $CDCl_3$): δ 12.71 (s, 1H), 8.35 (d, *J* = 8.1 Hz, 1H), 7.81 (d, *J* = 8.4 Hz, 1H), 7.61 (t, *J* = 7.3, 1H), 7.34 (t, *J* = 7.6 Hz, 1H), 6.25 (s, 1H), 3.26 (t, *J* = 6.7 Hz, 2H), 2.71 (t, *J* = 7.6 Hz, 2H), 1.76 – 1.71 (m, 4H), 1.44 – 1.37 (m, 2H); ^{13}C NMR (101 MHz, $CDCl_3$): δ 179.0, 155.2, 140.9, 132.0, 125.2, 125.0, 123.8, 118.8, 108.2, 34.2, 33.5, 32.4, 28.4, 27.8; IR (neat)/cm^{-1}: 1628, 1591, 1547, 760; HRMS (ESI-TOF) m/z: $[M + H]^+$ calcd. for $C_{14}H_{17}NOBr$, 294.0488; found, 294.0483.

2-(5-Bromopentyl)-4-chloroquinoline S26

A solution of compound **S25** (50 mg, 0.17 mmol, 1.0 eq) in phosphorus oxychloride (1 mL) was heated to reflux for 1 h. The excess phosphorus oxychloride was removed under reduced pressure and the resulting residue was dissolved in CH_2Cl_2 (5 mL) and washed with saturated $NaHCO_3$ (3 x 5 mL). The organic layer was the dried over $MgSO_4$, filtered and evaporated to dryness to give the title compound **S26** (37 mg, 0.13 mmol) as a colourless oil. The mixture was used in the next step without further purification.

TLC (EtOAc:hexane, 4:6 v/v): R_f = 0.19; ^{1}H NMR (400 MHz, $CDCl_3$): δ 8.19 (d, *J* = 8.5 Hz, 1H), 8.05 (d, *J* = 8.4 Hz, 1H), 7.74 (dd, *J* = 8.4, 6.9 Hz, 1H), 7.59 (dd, *J* = 8.3, 7.0 Hz, 1H), 7.40 (s, 1H), 3.42 (t, *J* = 6.8 Hz, 2H), 2.96 (t, *J* = 7.8 Hz, 2H), 1.97 – 1.82 (m, 4H),1.61-1.55 (m, 2H); ^{13}C NMR (126 MHz, $CDCl_3$) δ 162.5, 148.9, 142.8, 130.5, 129.3, 126.9, 125.1, 124.1, 121.5, 77.2, 38.9, 33.8, 32.7, 29.9, 28.9, 28.1; IR (neat)/cm^{-1}: 1589, 1553, 839, 760); HRMS (ESI-TOF) m/z: $[M + H]^+$ calcd. for $C_{14}H_{16}BrClN$: 312.0155; found: 312.0416.

4-Azido-2-(5-azidopentyl)quinolone 11

A solution of compound **S26** (31 mg, 0.1 mmol, 1.0 eq) and sodium azide (30 mg, 0.5 mmol, 2.5 eq) in DMF (0.5 mL) was heated to 55 °C overnight with stirring. The reaction was quenched by the addition of water and the mixture was stirred for 5 min. The resulting mixture was extracted with EtOAc (3 x 20 mL), the organic phase was washed with water (2 x 20 mL), dried over $MgSO_4$, filtered and evaporated to dryness. The crude product was purified by column chromatography (stepwise gradient of 0-20% EtOAc in hexane) to give **11** (21 mg, 0.074 mmol) a colourless oil in 74% yield.

TLC (EtOAc:hexane, 2:8 v/v): R_f = 0.17; ^{1}H NMR (500 MHz,$CDCl_3$): δ 8.02 – 7.99 (m, 2H), 7.71 (ddd, *J* = 8.4, 6.9, 1.5 Hz, 1H), 7.48 (ddd, *J* = 8.3, 6.9, 1.2 Hz, 1H), 7.02 (s, 1H), 3.29 (t, *J* = 6.9 Hz, 2H), 2.97 (t, *J* = 7.9 Hz, 2H), 1.93 – 1.82 (m, 2H), 1.73 – 1.64 (m, 2H), 1.55 – 1.47 (m, 2H); ^{13}C NMR (126 MHz, $CDCl_3$): δ 162.8, 149.1, 146.4, 130.6, 128.8, 125.9, 122.2, 120.4, 108.7, 51.5, 39.3, 29.5, 28.9, 26.8; IR (neat)/cm^{-1}: 2107, 2091, 1681, 1591, 1560, 1502.

4.3.12 Synthesis of negative control probe 12

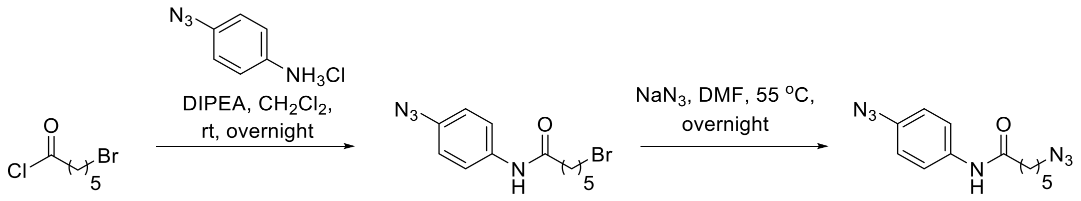

S27

12
54% (2 steps)

N-(4-Azidophenyl)-6-bromohexanamide S27

A solution of 4-azidoaniline hydrochloride (200 mg, 1.17 mmol, 1.0 eq), 6-bromohexanoyl chloride (180 µL, 1.16 mmol, 1.0 eq) and DIPEA (205 µL, 2.33 mmol, 2.0 eq) in CH_2Cl_2 (5 mL) was stirred overnight at room temperature. The reaction was quenched by pouring into saturated aqueous solution of $NaHCO_3$ and the mixture was stirred for 5 min. The resulting mixture was extracted with CH_2Cl_2 (3 x 20 mL) and the organic phase was washed with 0.1 M HCl (1 x 20 mL), saturated $NaHCO_3$ (1 x 20 mL), water (1 x 20 mL) and then dried over $MgSO_4$, filtered and evaporated to dryness to give **S27** (288 mg) as an impure brown

amorphous solid (100% yield assumed, contaminated with free acid chlorgide) which was used without further purification.

TLC (EtOAc:hexane, 2:8 v/v): R_f = 0.37; ^{1}H NMR (500 MHz, DMSO-d_6): δ 9.95 (s, 1H), 7.66 – 7.60 (m, 2H), 7.08 – 7.02 (m, 2H), 3.54 (t, *J* = 6.7 Hz, 2H), 2.30 (t, *J* = 7.4 Hz, 2H), 1.84 – 1.80 (m, 2H), 1.61 (app p, *J* = 7.6 Hz, 2H), 1.44 - 1.40 (m, 2H); ^{13}C NMR (126 MHz, DMSO-d_6): δ 174.3 (acid chloride), 171.0, 136.6, 133.5 (acid chloride), 120.5, 119.4, 39.5, 35.1, 32.0, 27.2, 24.2; IR (neat)/cm^{-1}: 3323, 2108, 1655, 1598, 1507; HRMS (ESI-TOF) m/z: $[M + H]^+$ calcd. for $C_{12}H_{16}N_4OBr$: 311.0502; found: 311.0492.

6-Azido-N-(4-azidophenyl)hexanamide 12

A solution of crude **S27** (200 mg, ~ 0.4 mmol, 1.0 eq) and NaN_3 (72 mg, 1.2 mmol, 3.0 eq) in DMF (1 mL) was heated to 55 °C overnight with stirring. The reaction was quenched by pouring into water and the resulting mixture was extracted with EtOAc (3 x 20 mL). The combined organic phases were washed with water (2 x 20 mL), dried over $MgSO_4$, filtered and evaporated to dryness to give a yellow oil. The crude product was purified by column chromatography (stepwise gradient of 0-20% EtOAc in hexane) to give a **12** (64 mg, 0.22 mmol) as a colourless oil which crystallised on standing in 54% yield.

mp: 51.5-52.3 ˚C (EtOAc); TLC (EtOAc:hexane, 2:8 v/v): R_f = 0.37; ^{1}H NMR (500 MHz, $CDCl_3$): δ 7.51 (d, *J* = 8.9 Hz, 2H), 7.11 (s, 1H), 6.98 (d, *J* = 8.8 Hz, 2H), 3.29 (t, *J* = 6.8 Hz, 2H), 2.37 (t, *J* = 7.4 Hz, 2H), 1.77 (app p, *J* = 7.5 Hz, 2H), 1.65 (dt, *J* = 14.6, 6.9 Hz, 2H), 1.51 – 1.43 (m, 2H); ^{13}C NMR (126 MHz, $CDCl_3$): δ 170.9, 135.9, 135.0, 121.4, 119.7, 51.4, 37.5, 28.8, 26.5, 25.1; IR(neat)/cm^{-1}: 3286, 2083, 1654, 1596, 1533, 1505; HRMS (ESI-TOF) m/z: $[M + H]^+$ calcd. for $C_{12}H_{15}N_7ONa$: 296.1230; found: 296.1219.

4.4 NMR spectra

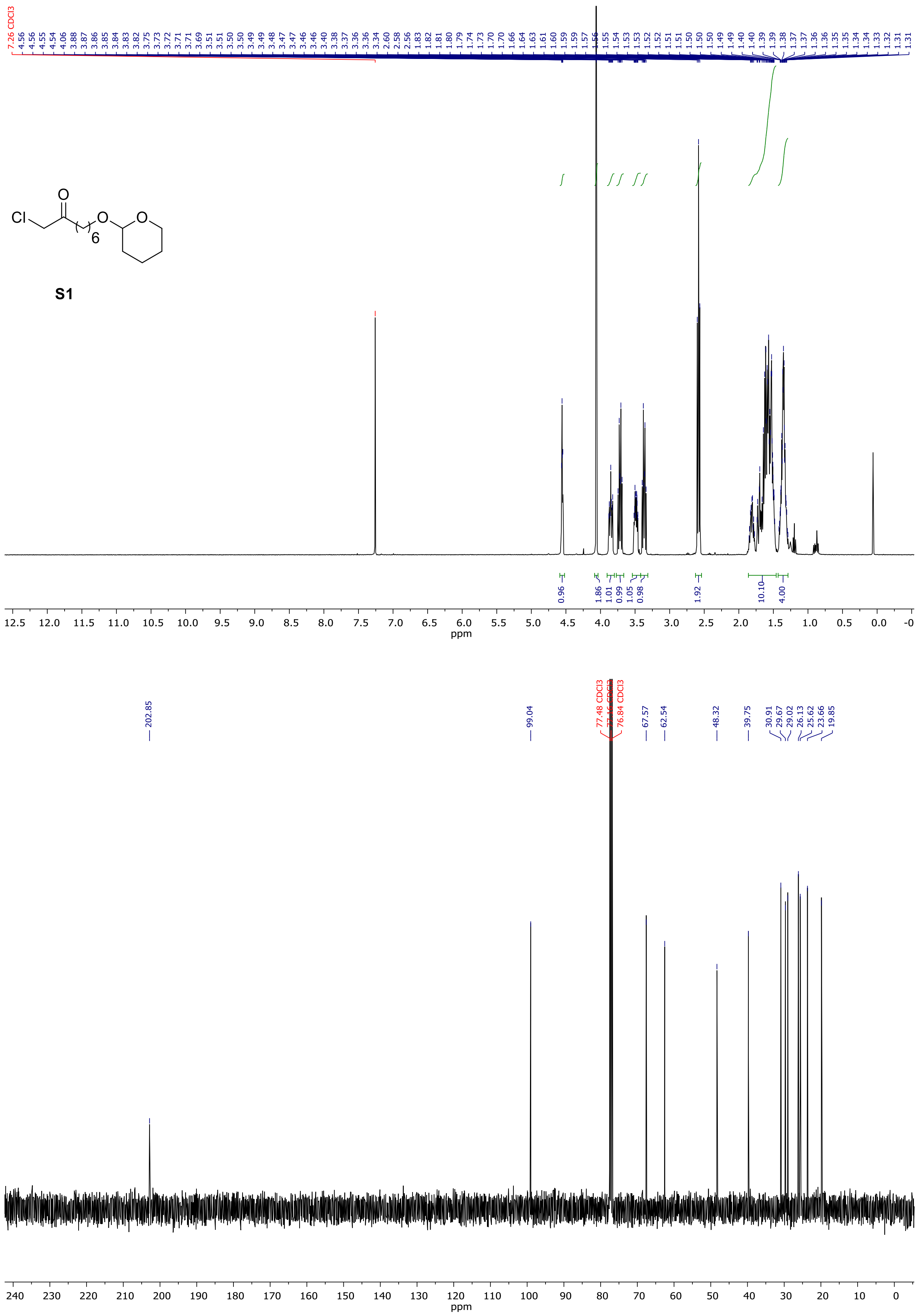

Supplementary Figure 9. ^{1}H and ^{13}C NMR of **S1**.

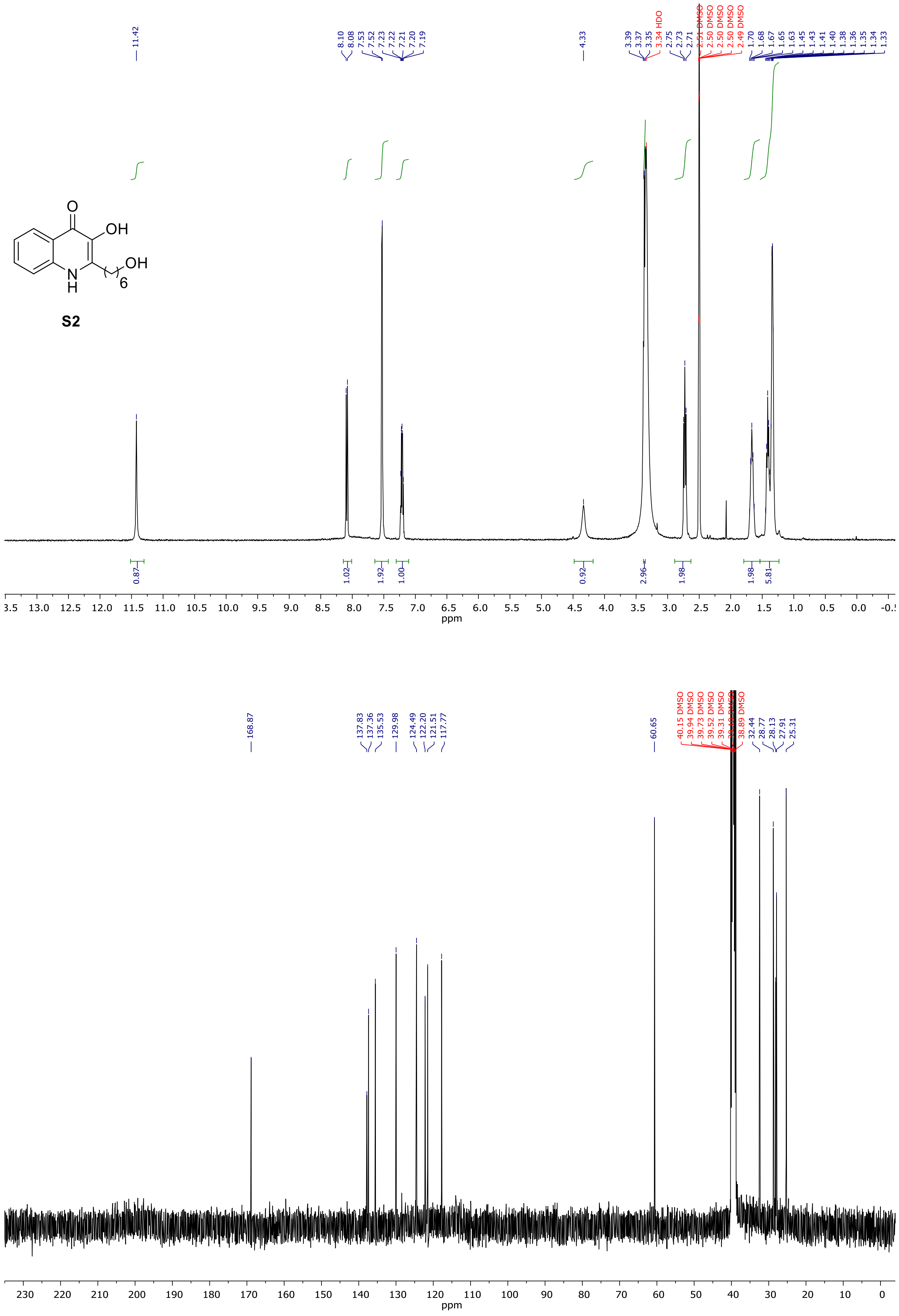

Supplementary Figure 10. ^{1}H and ^{13}C NMR of **S2**.

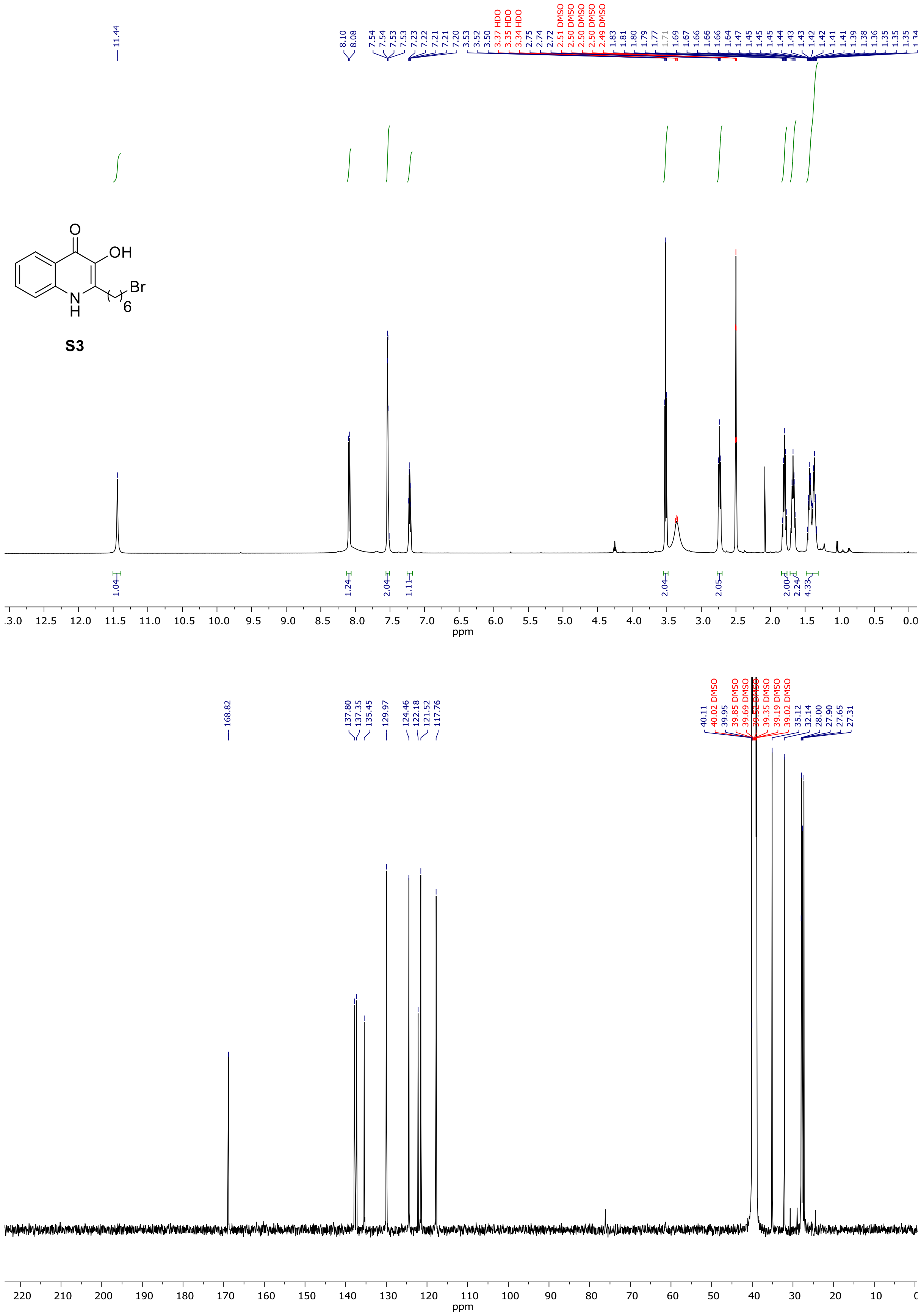

Supplementary Figure 11. ^{1}H and ^{13}C NMR of **S3**.

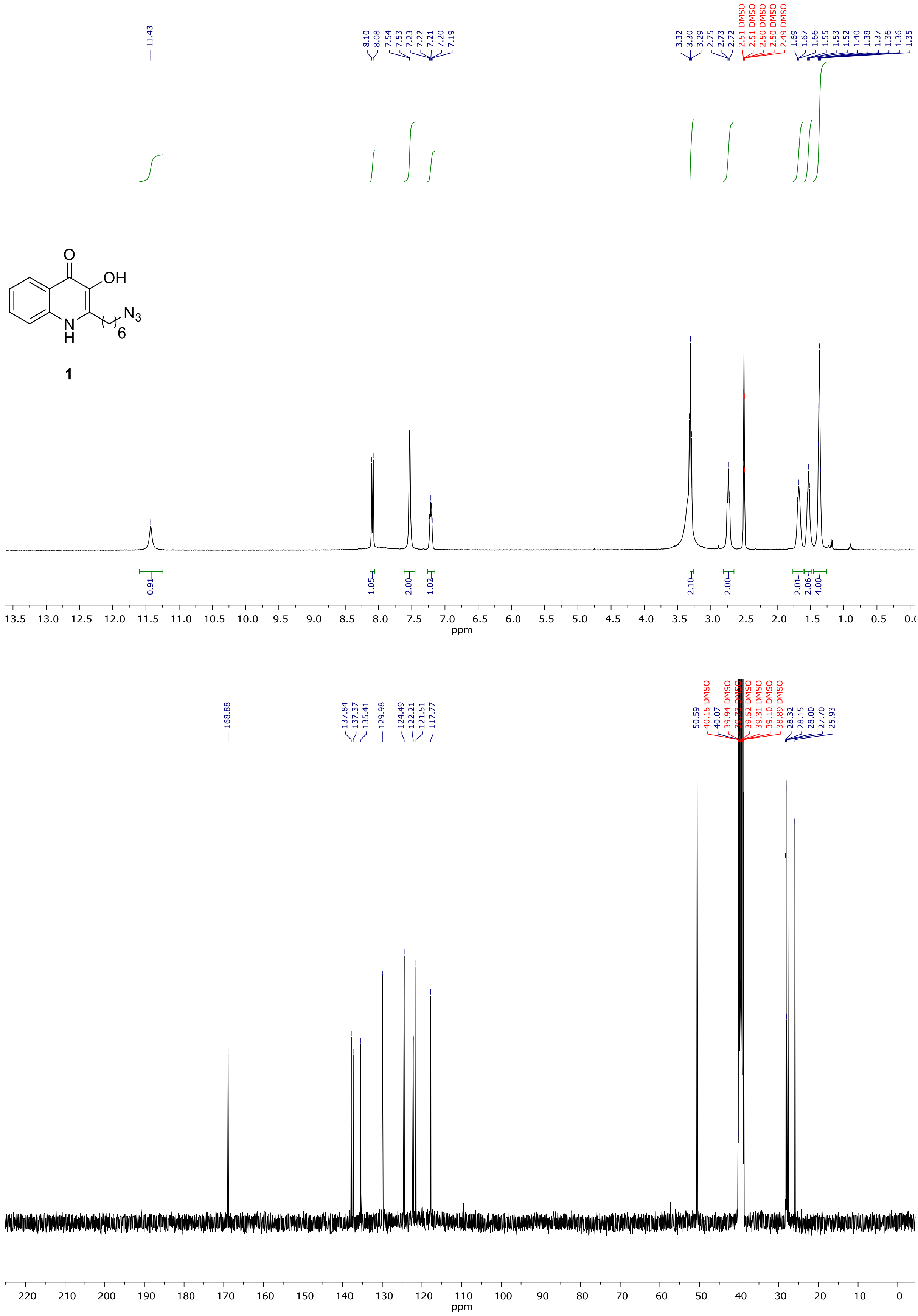

Supplementary Figure 12. ^{1}H and ^{13}C NMR of **1**.

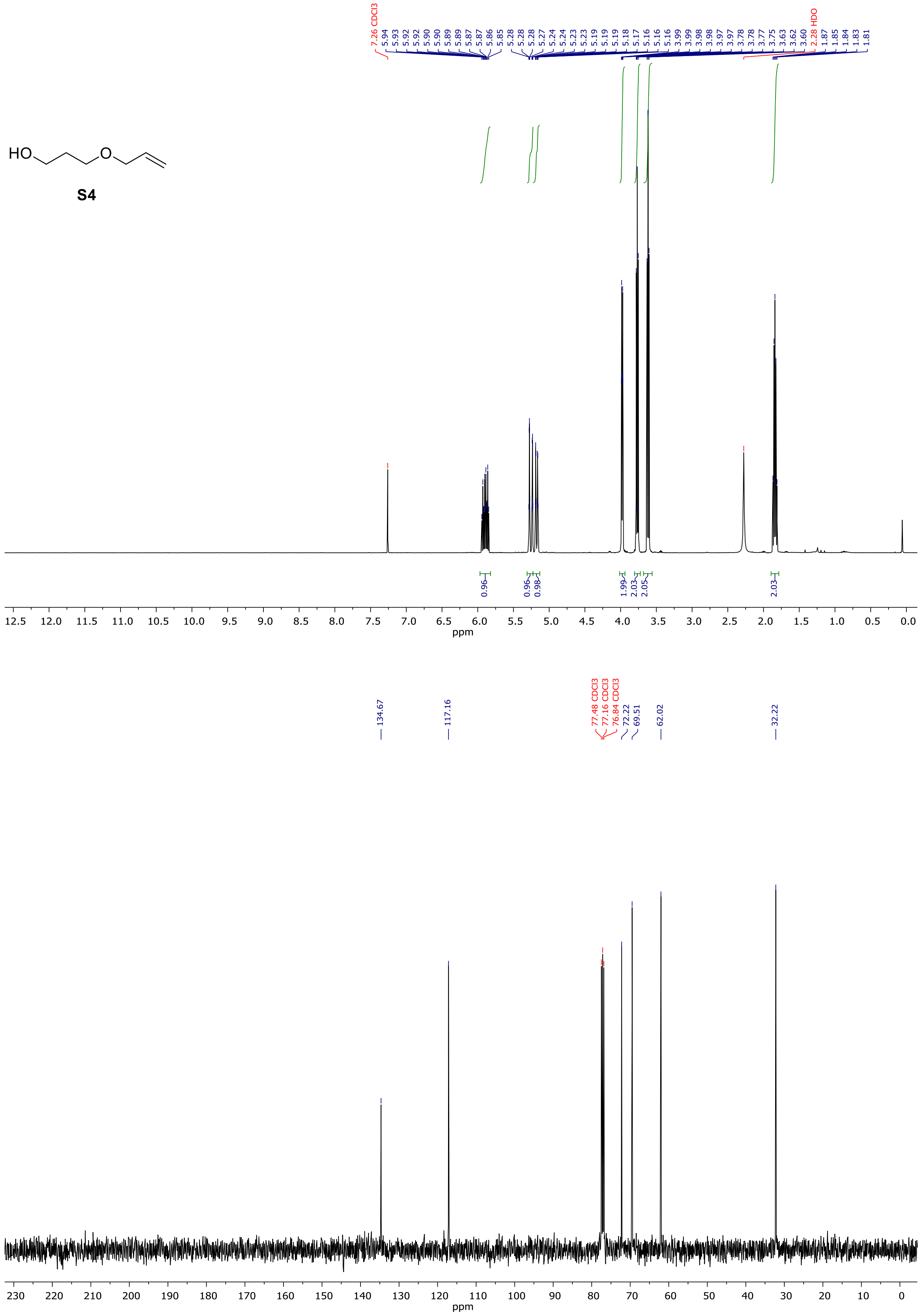

Supplementary Figure 13. ^{1}H and ^{13}C NMR of **S4**.

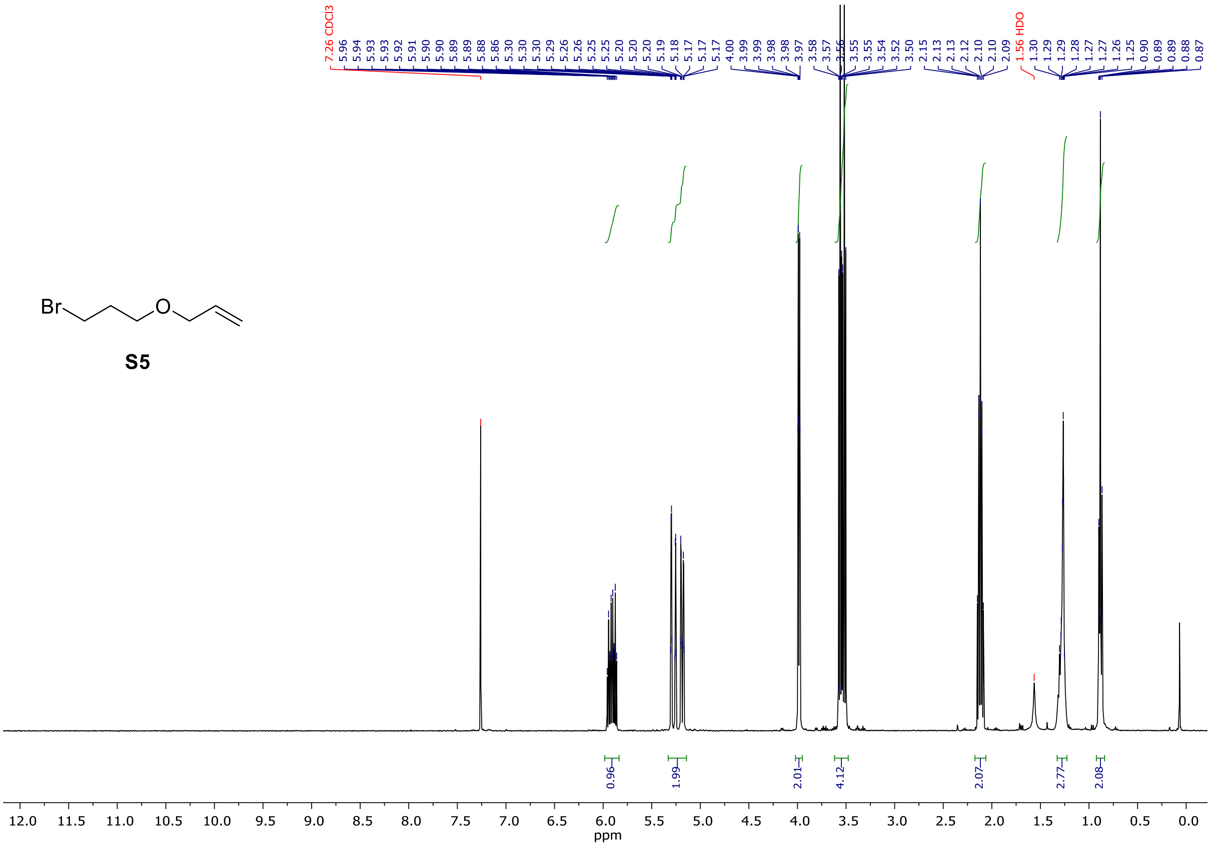

S5

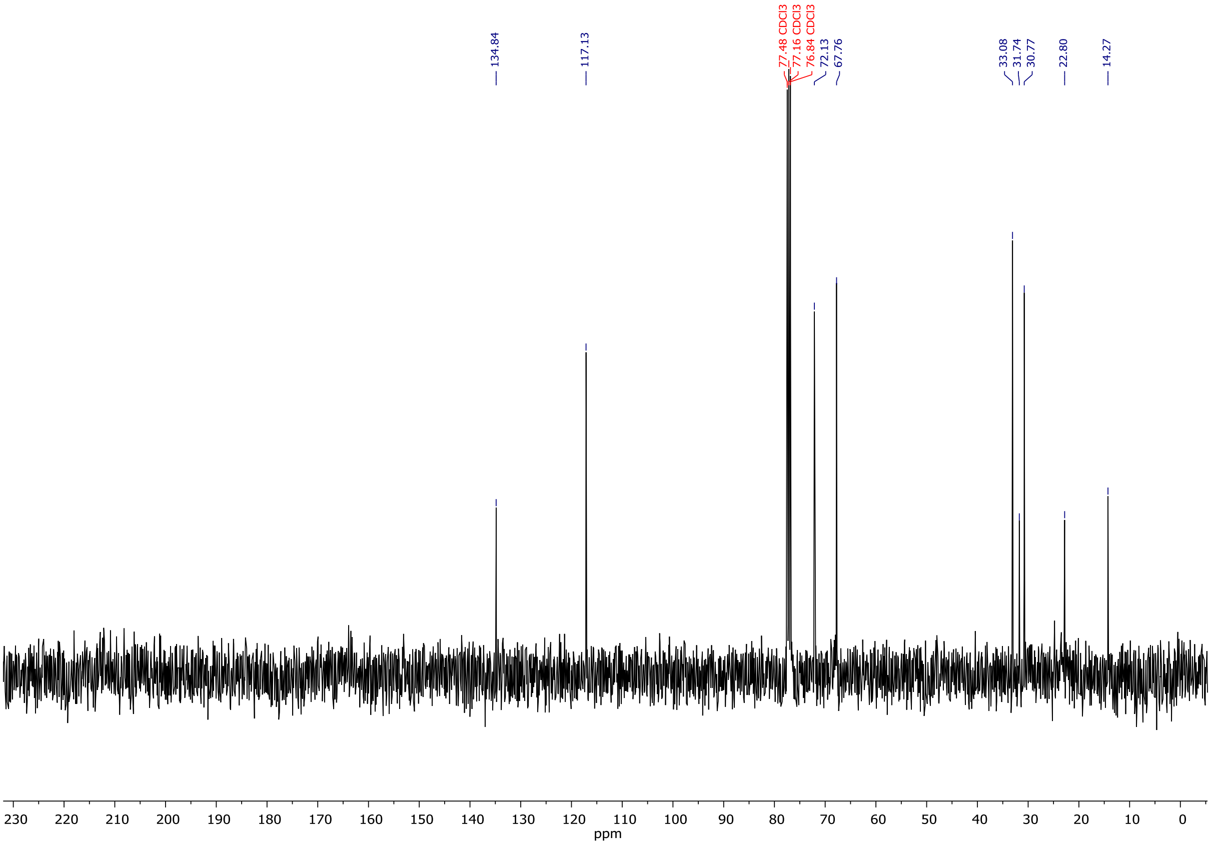

Supplementary Figure 14. ^{1}H and ^{13}C NMR of **S5**.

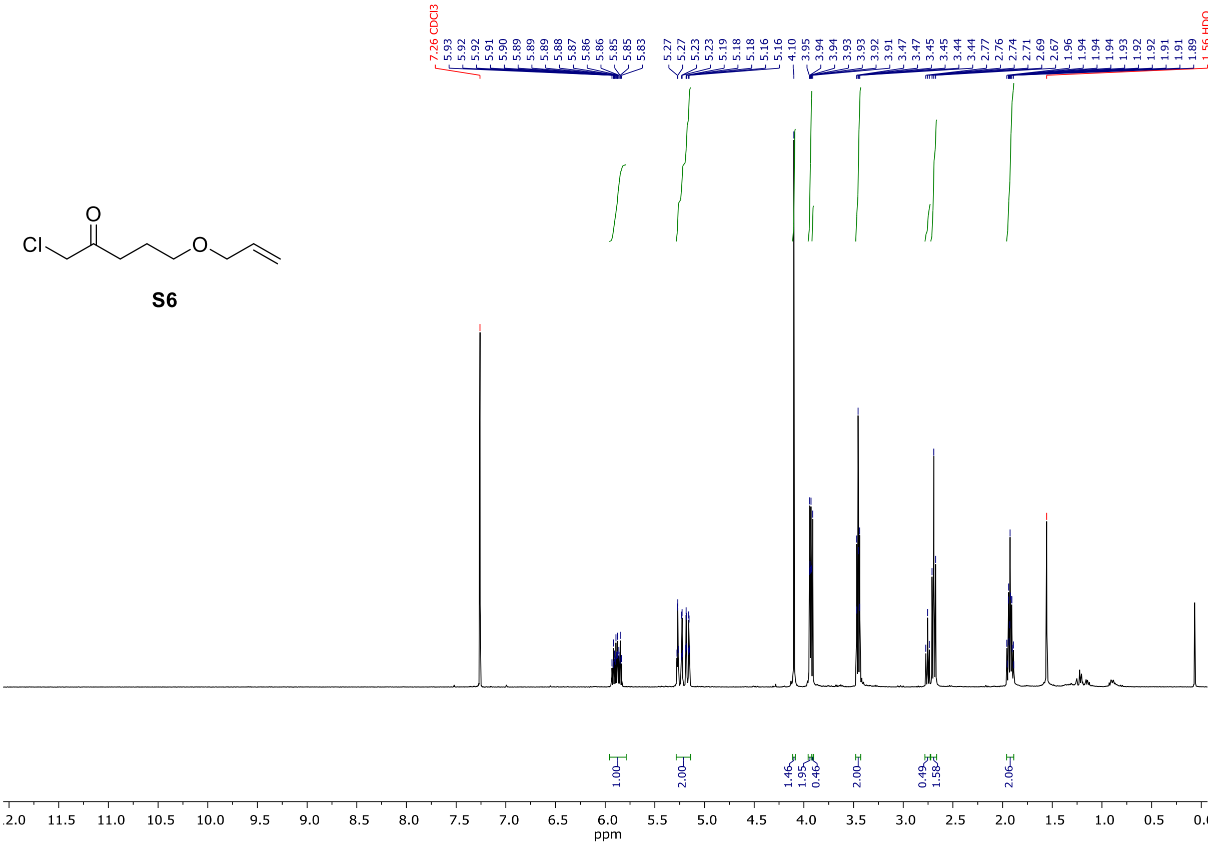

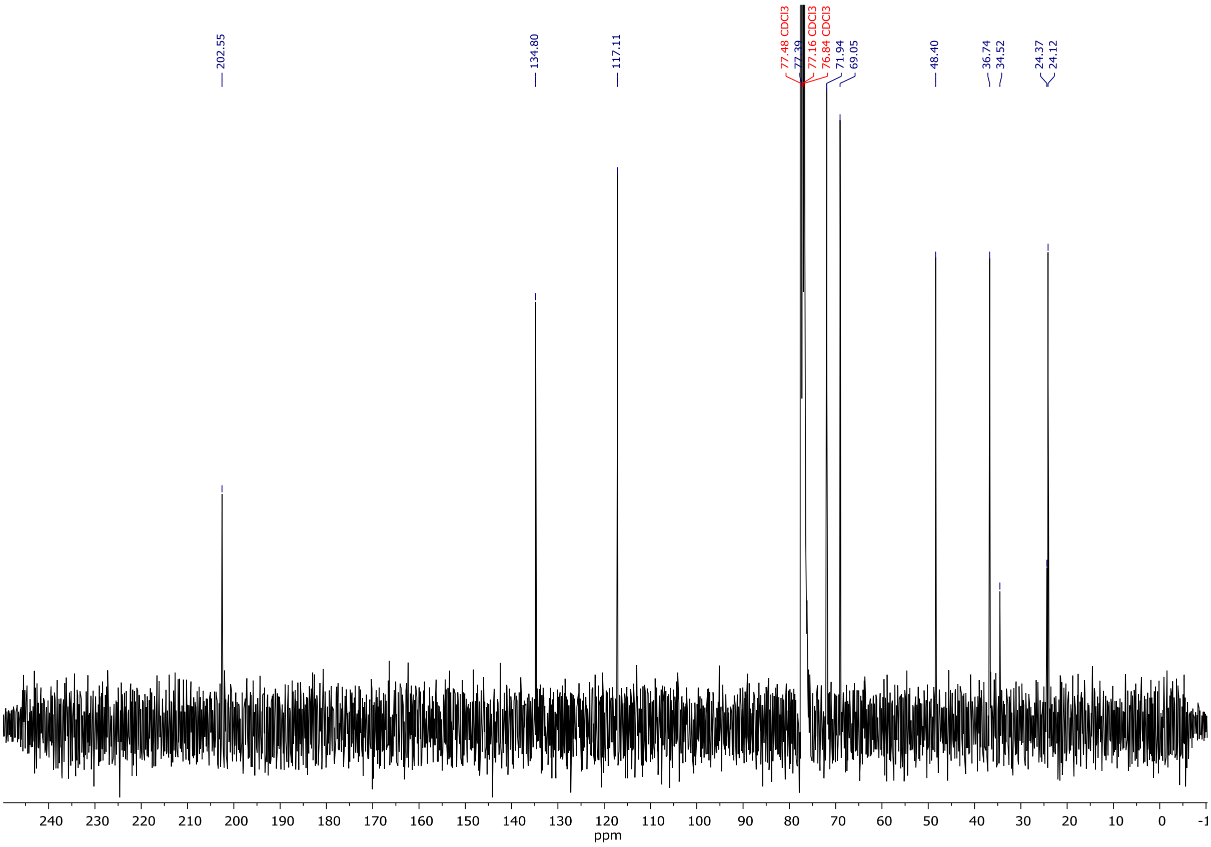

Supplementary Figure 15. ^{1}H and ^{13}C NMR of **S6**.

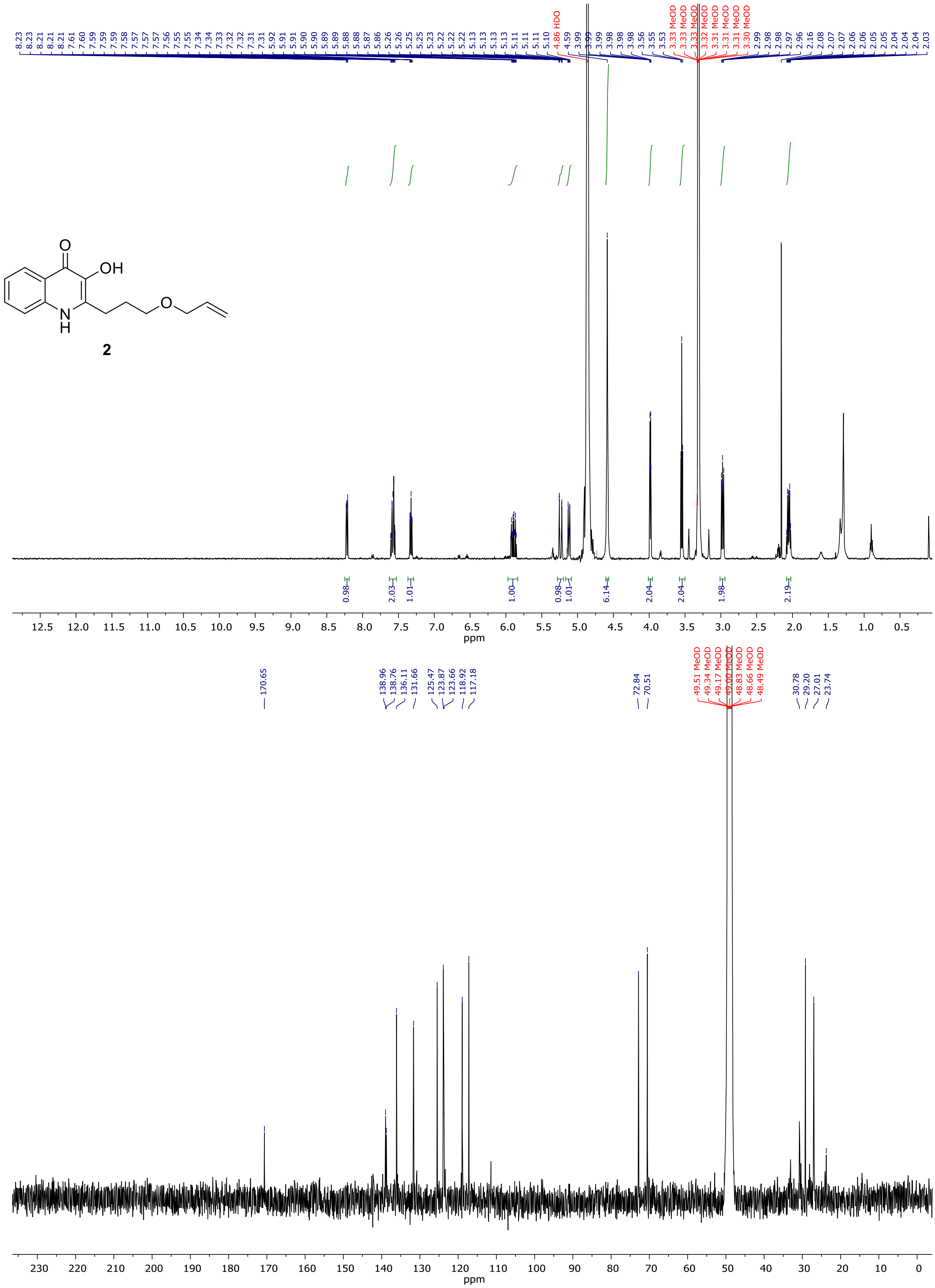

Supplementary Figure 16. ^{1}H and ^{13}C NMR of **2**.

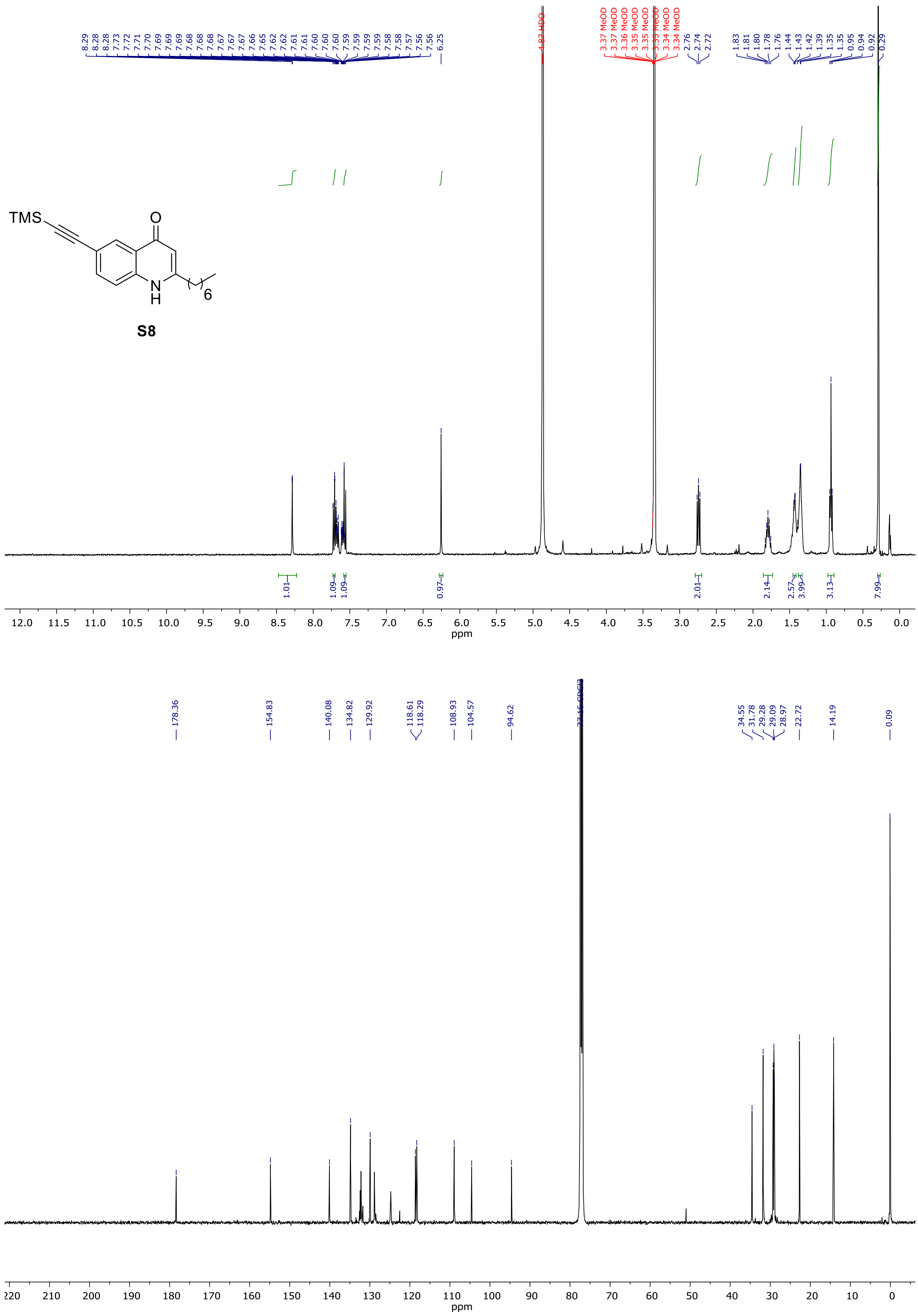

Supplementary Figure 17. ^{1}H and ^{13}C NMR of **S8**.

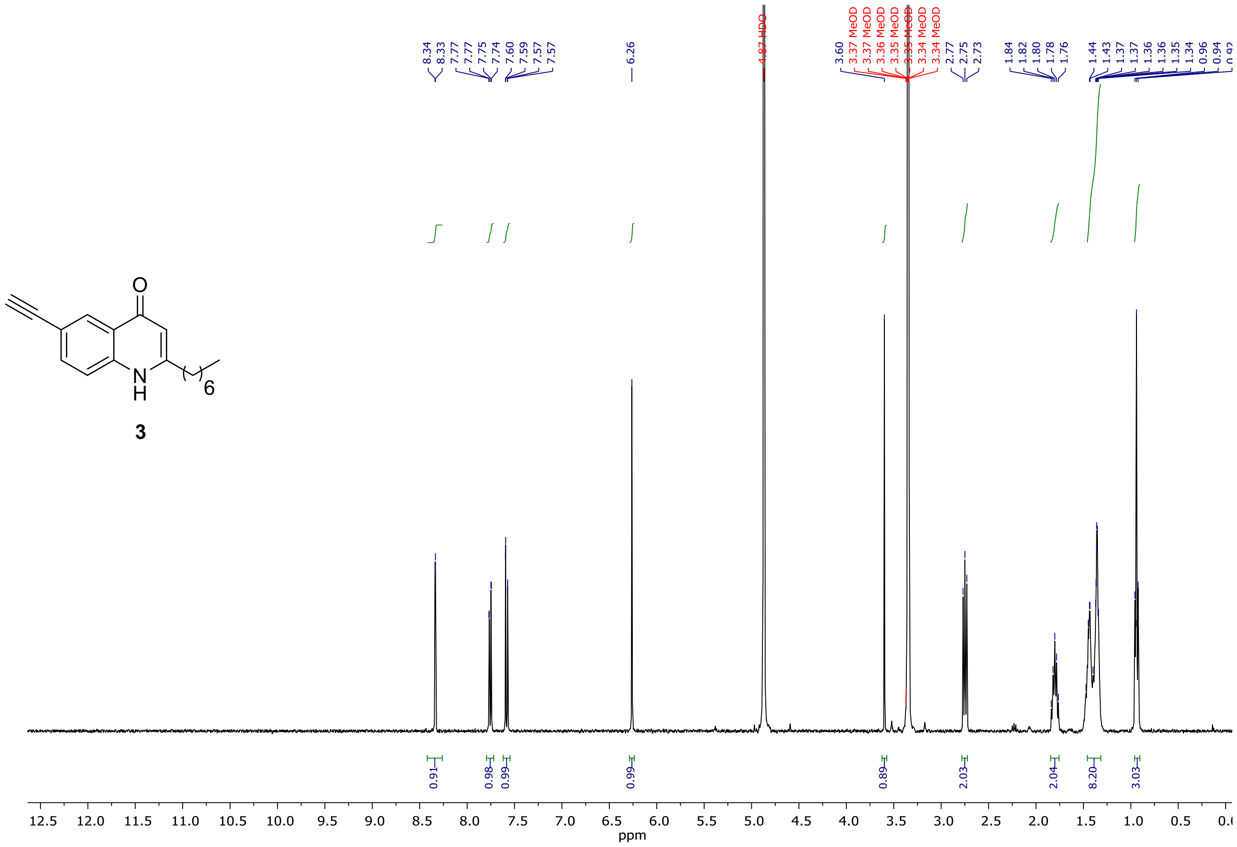

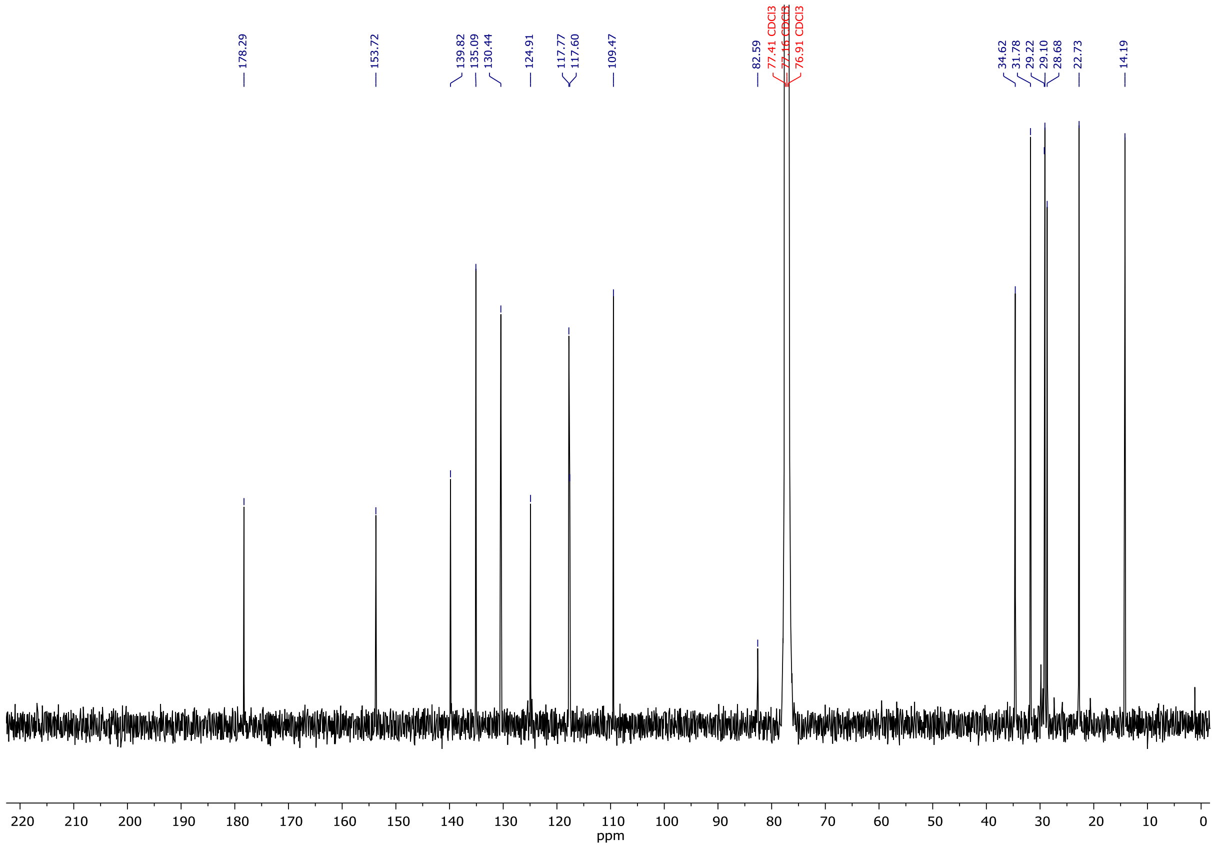

Supplementary Figure 18. ^{1}H and ^{13}C NMR of **3**.

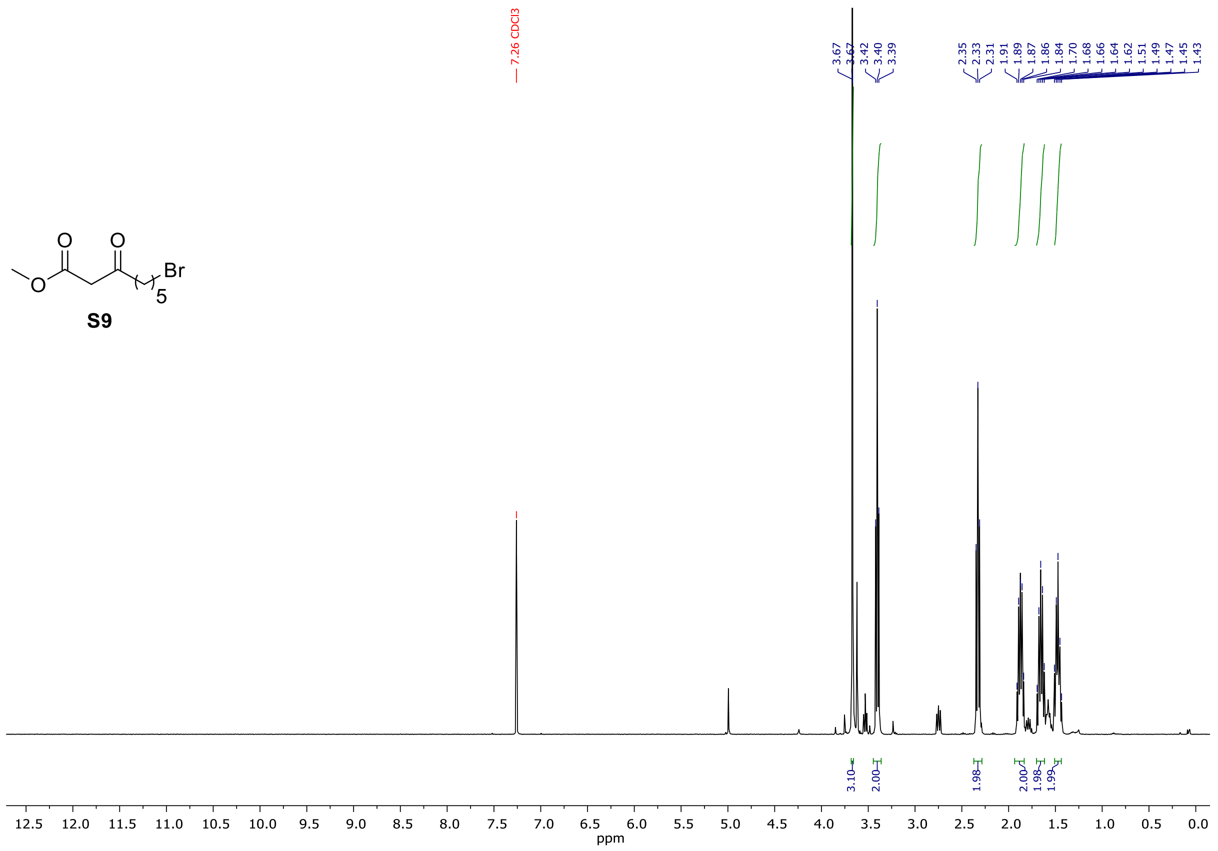

S9

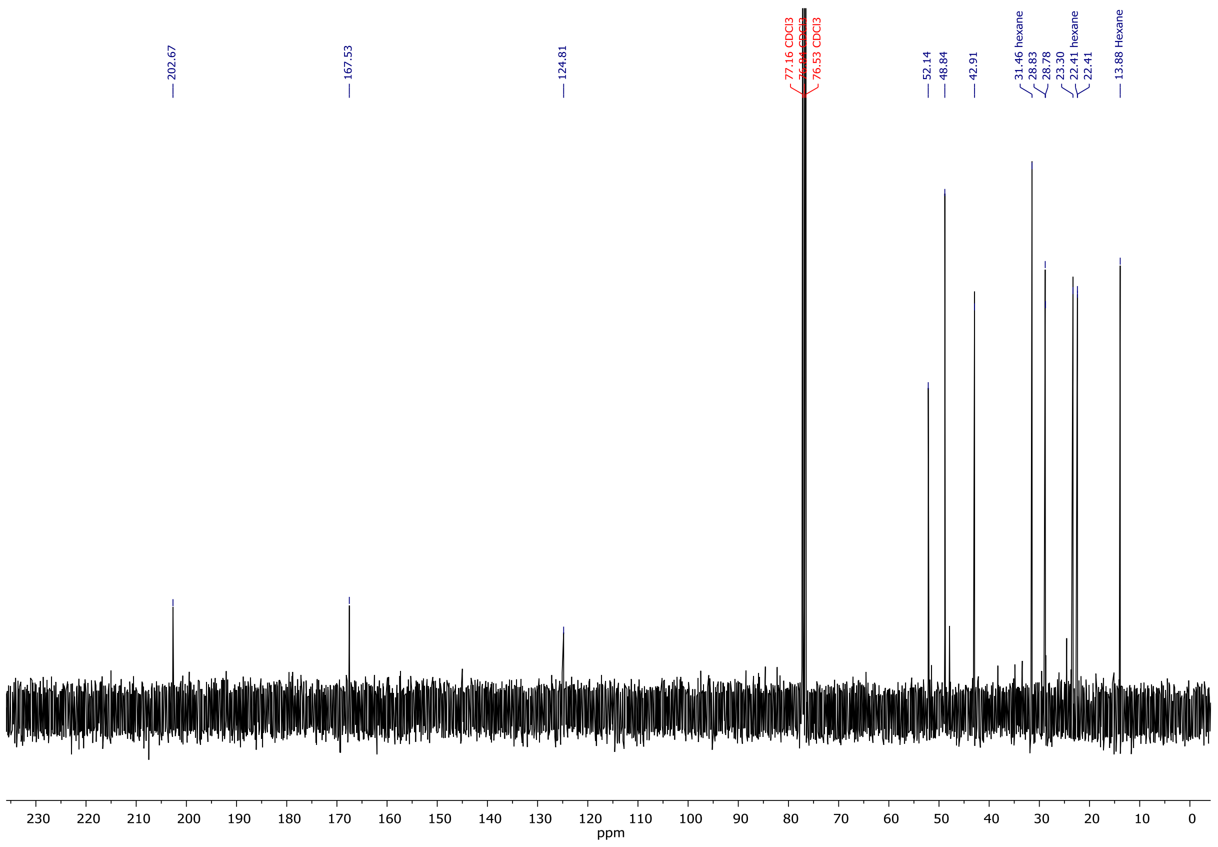

Supplementary Figure 19. ^{1}H and ^{13}C NMR of **S9**.

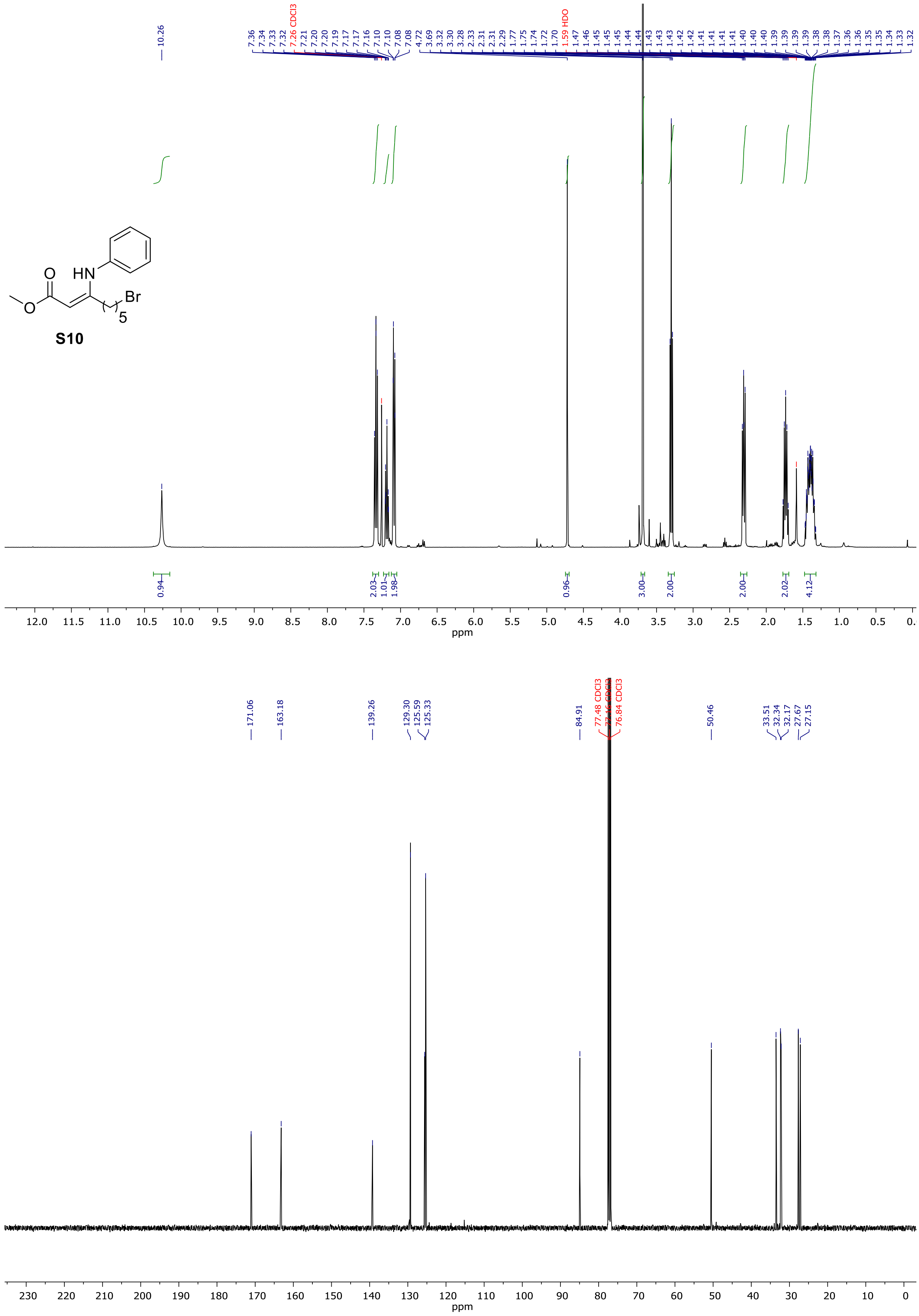

Supplementary Figure 20. ^{1}H and ^{13}C NMR of **S10**.

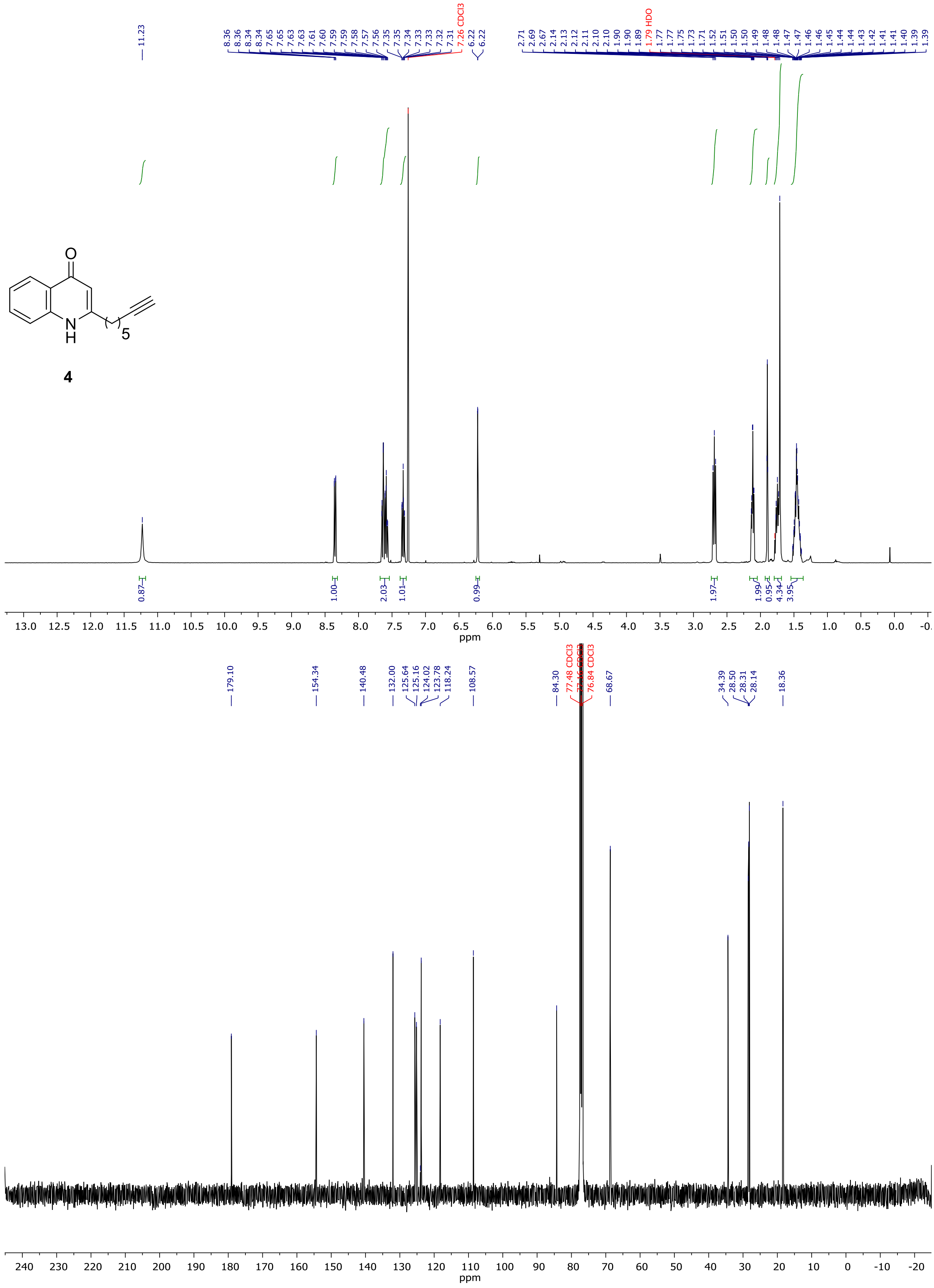

Supplementary Figure 21. ^{1}H and ^{13}C NMR of **4**.

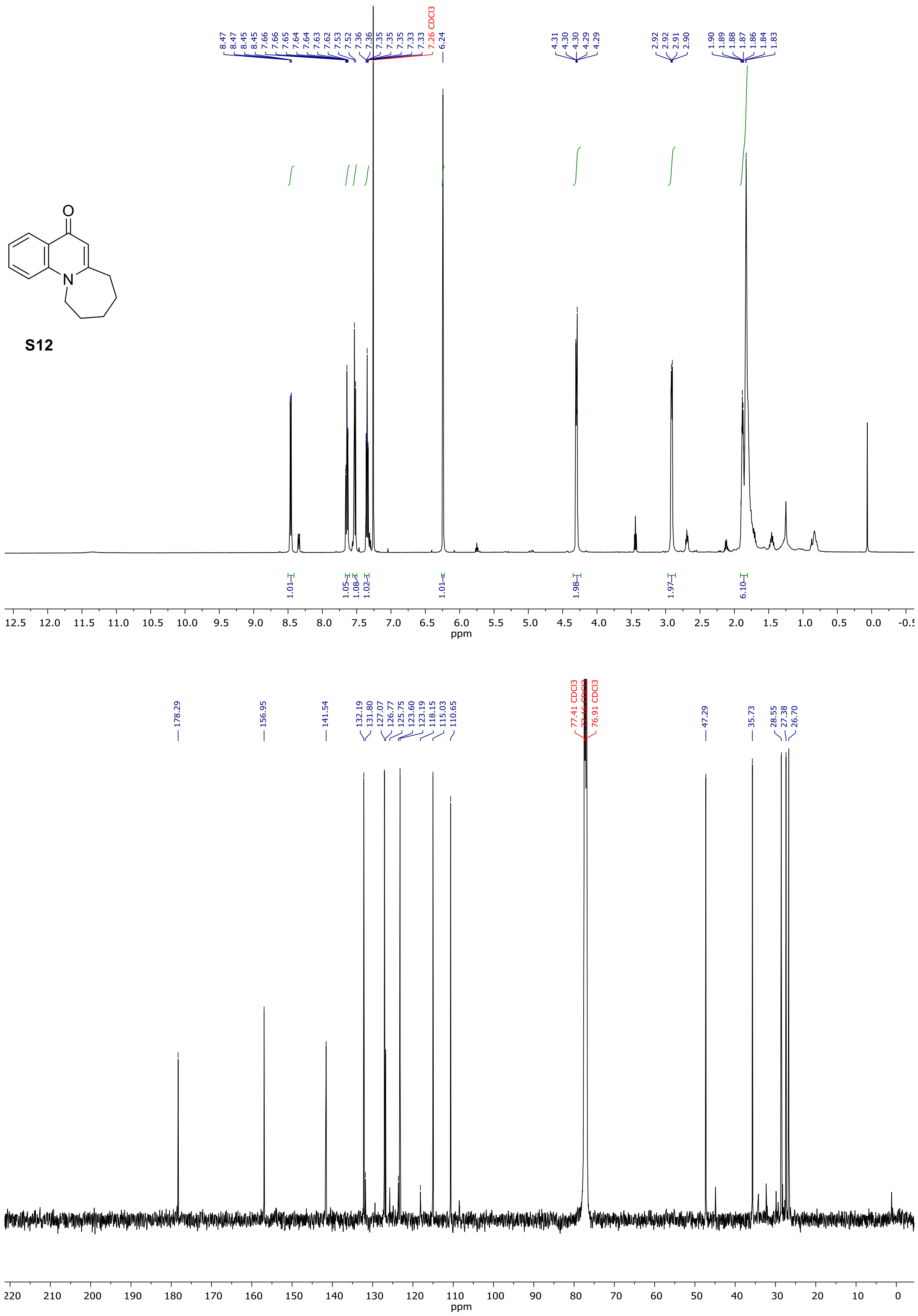

Supplementary Figure 22. ^{1}H and ^{13}C NMR of **S12**.

5

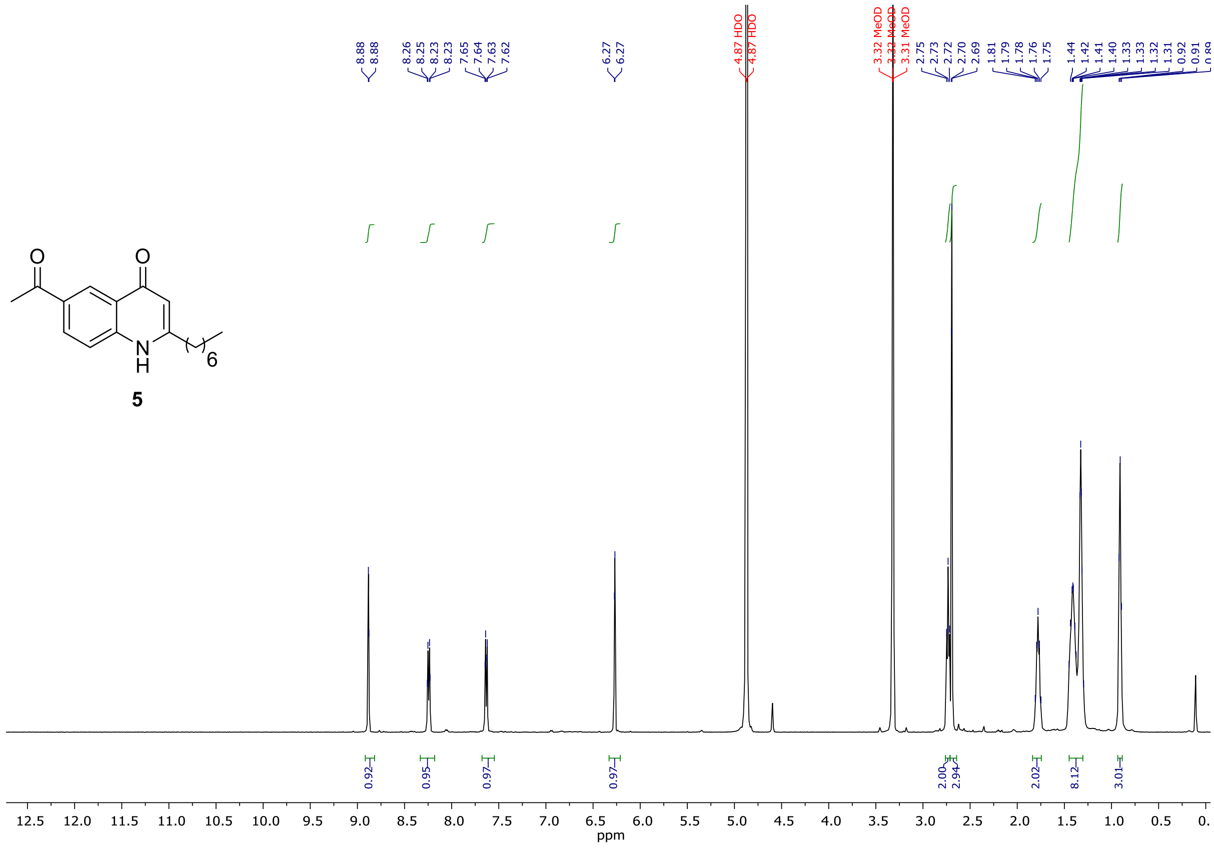

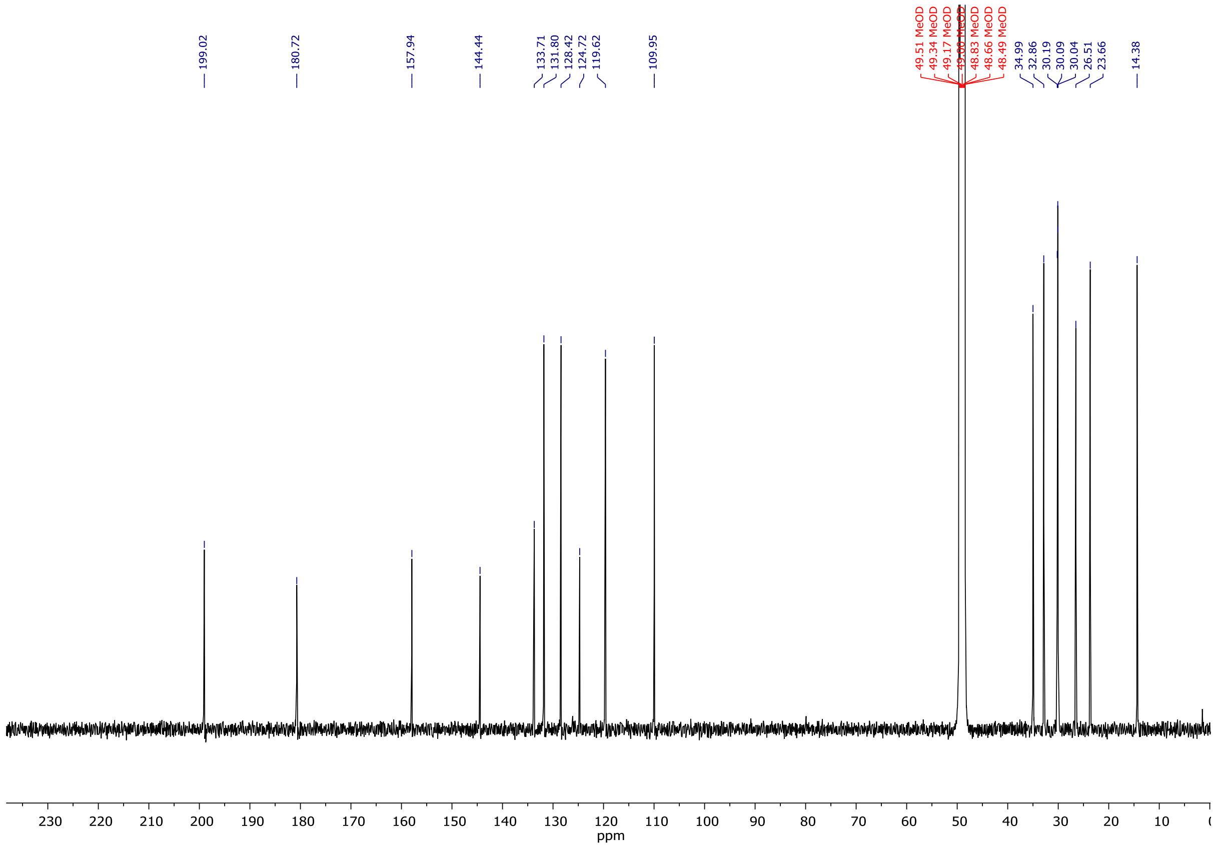

Supplementary Figure 23. ^{1}H and ^{13}C NMR of **5**.

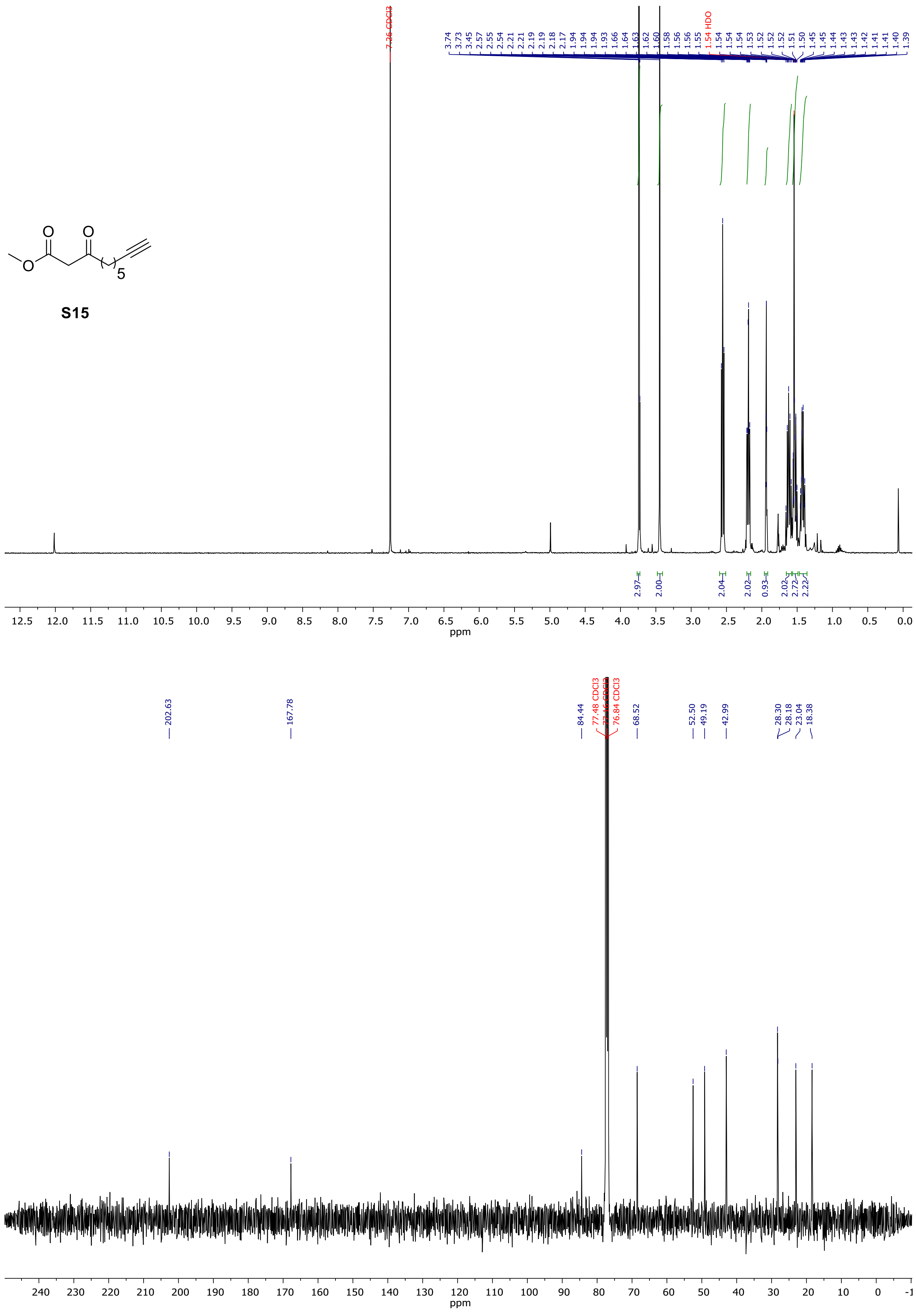

Supplementary Figure 24. ^{1}H and ^{13}C NMR of **S15**.

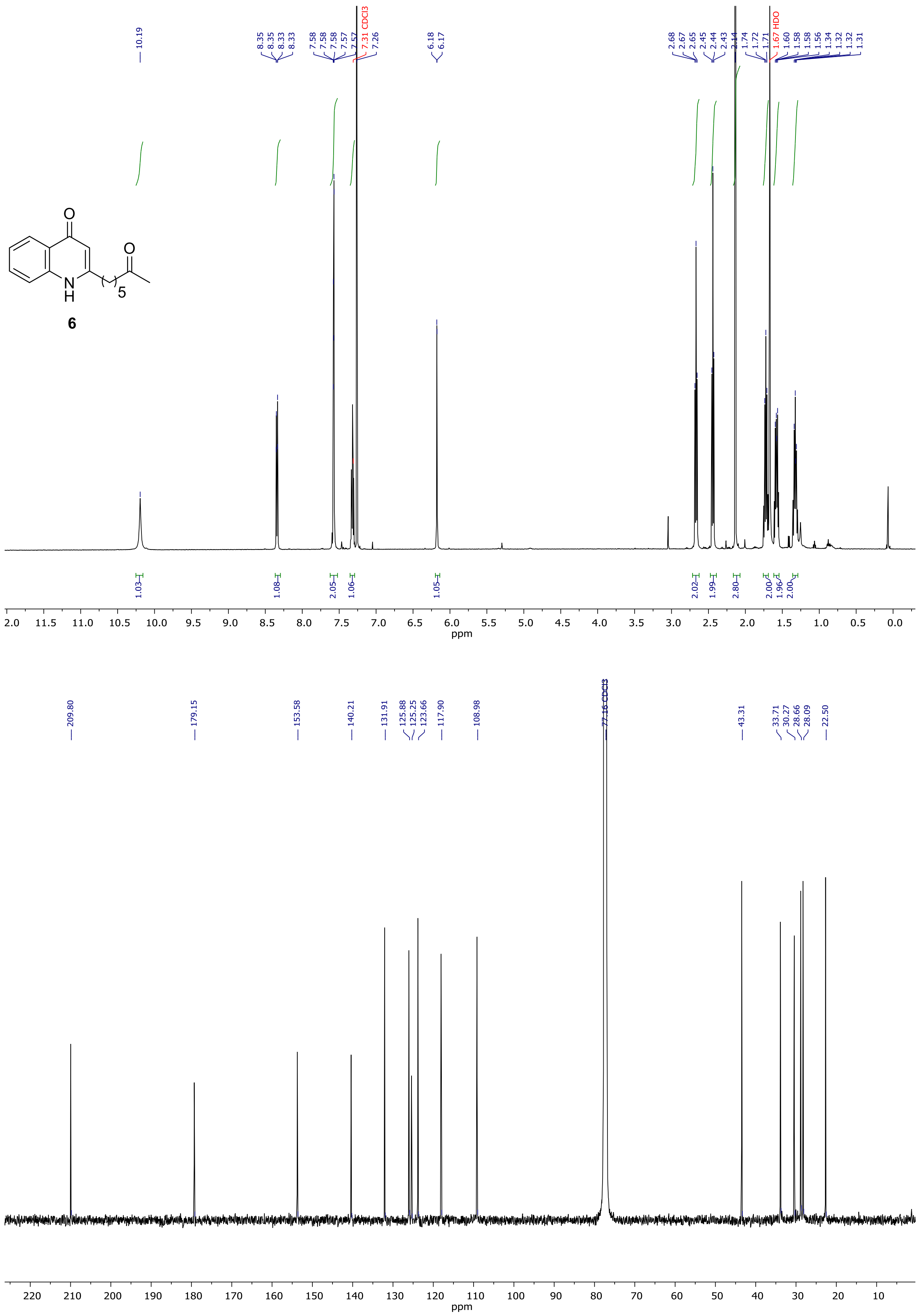

Supplementary Figure 25. ^{1}H and ^{13}C NMR of **6**.

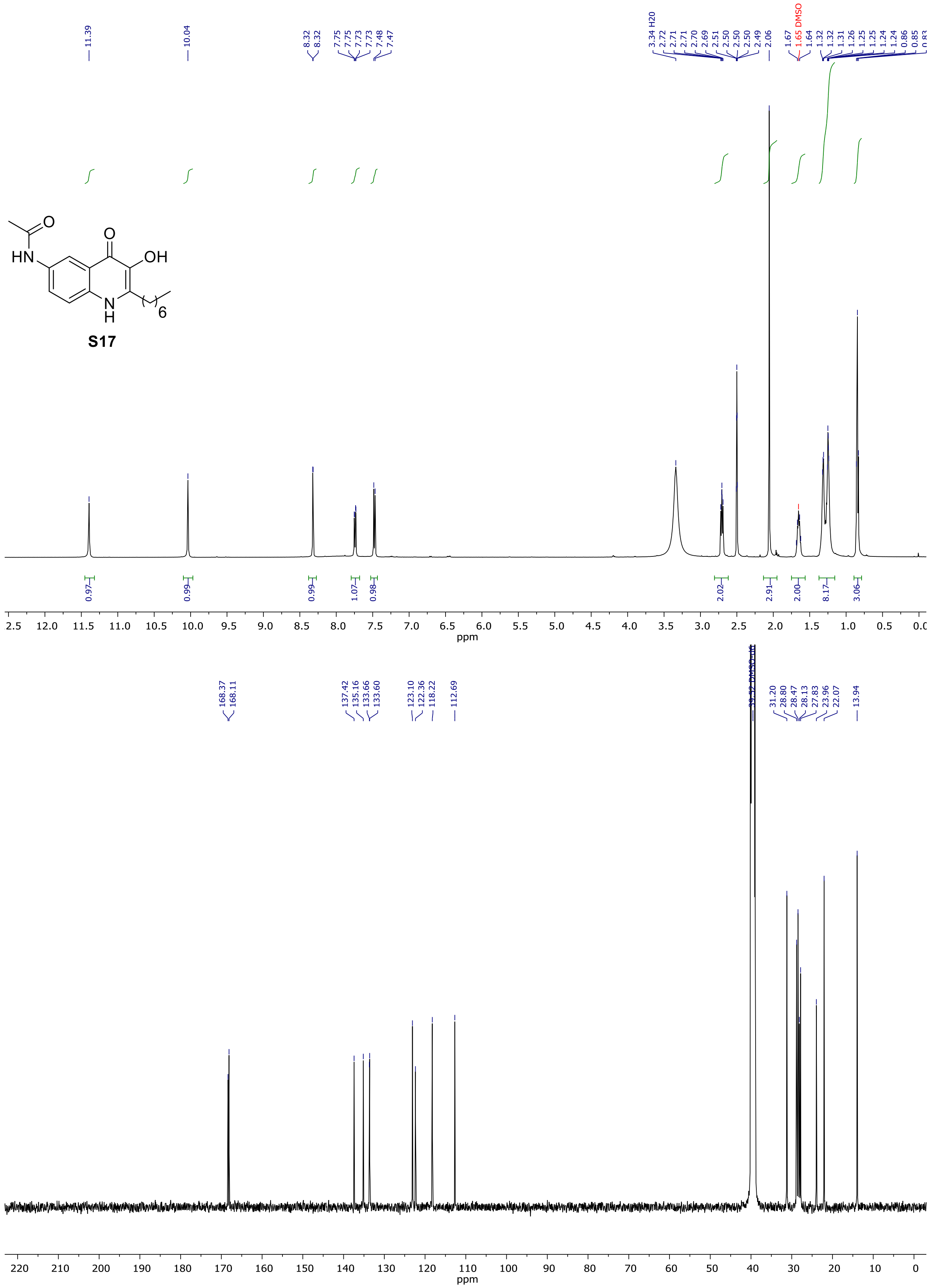

Supplementary Figure 26. ^{1}H and ^{13}C NMR of **S17**.

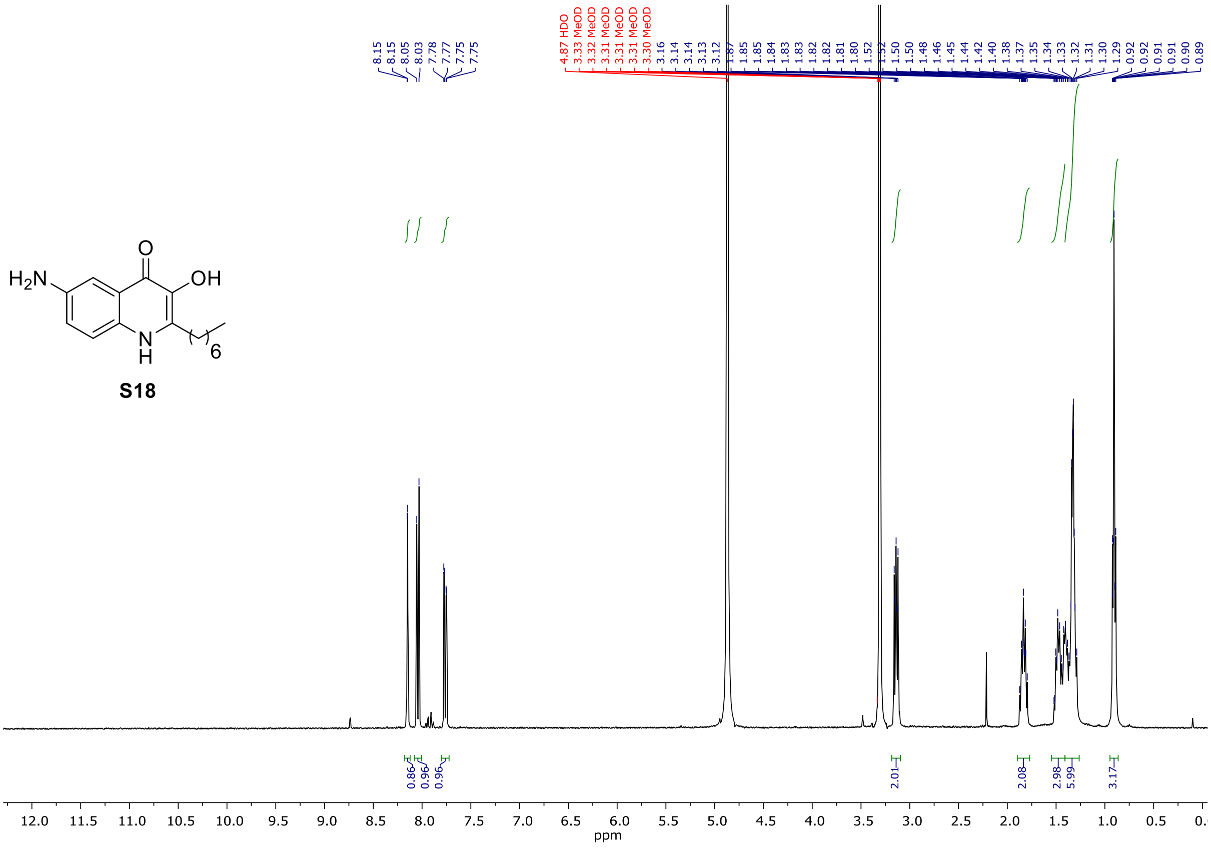

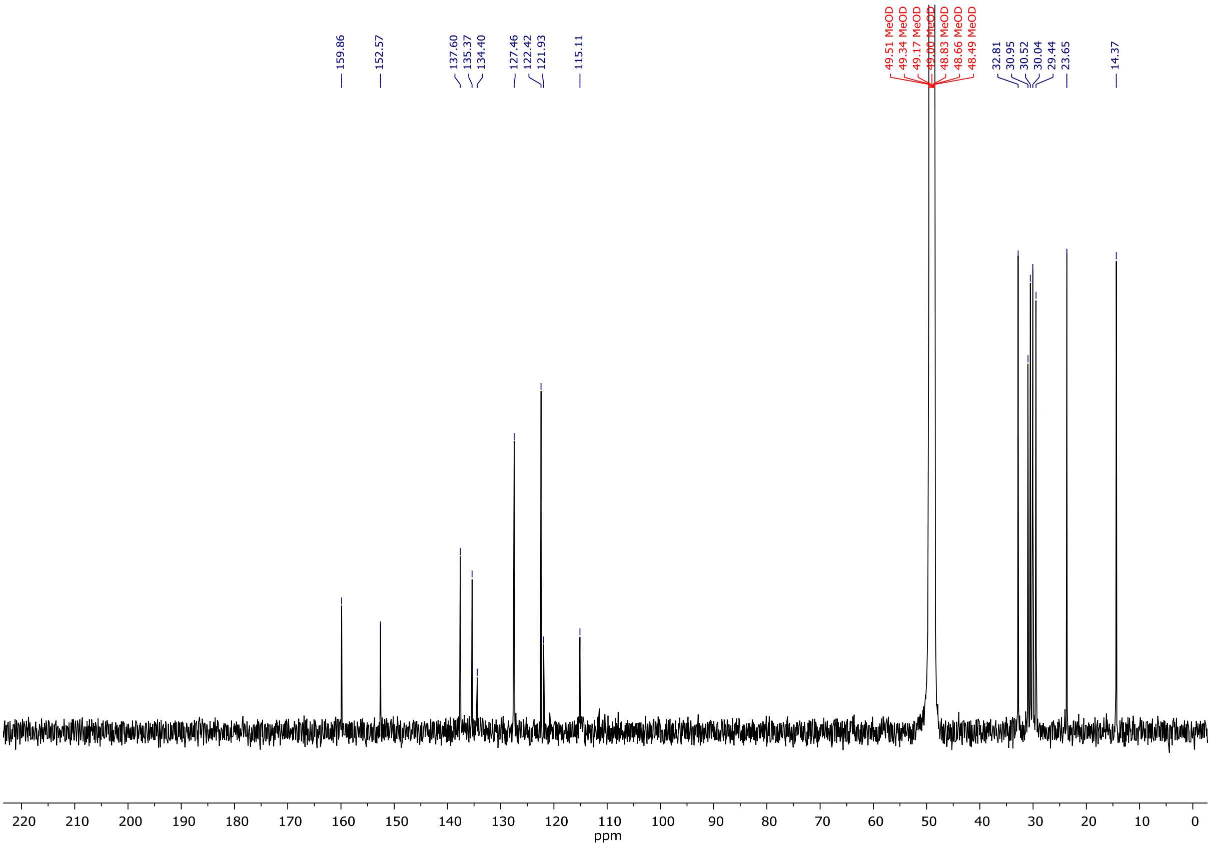

Supplementary Figure 27. ^{1}H and ^{13}C NMR of **S18**

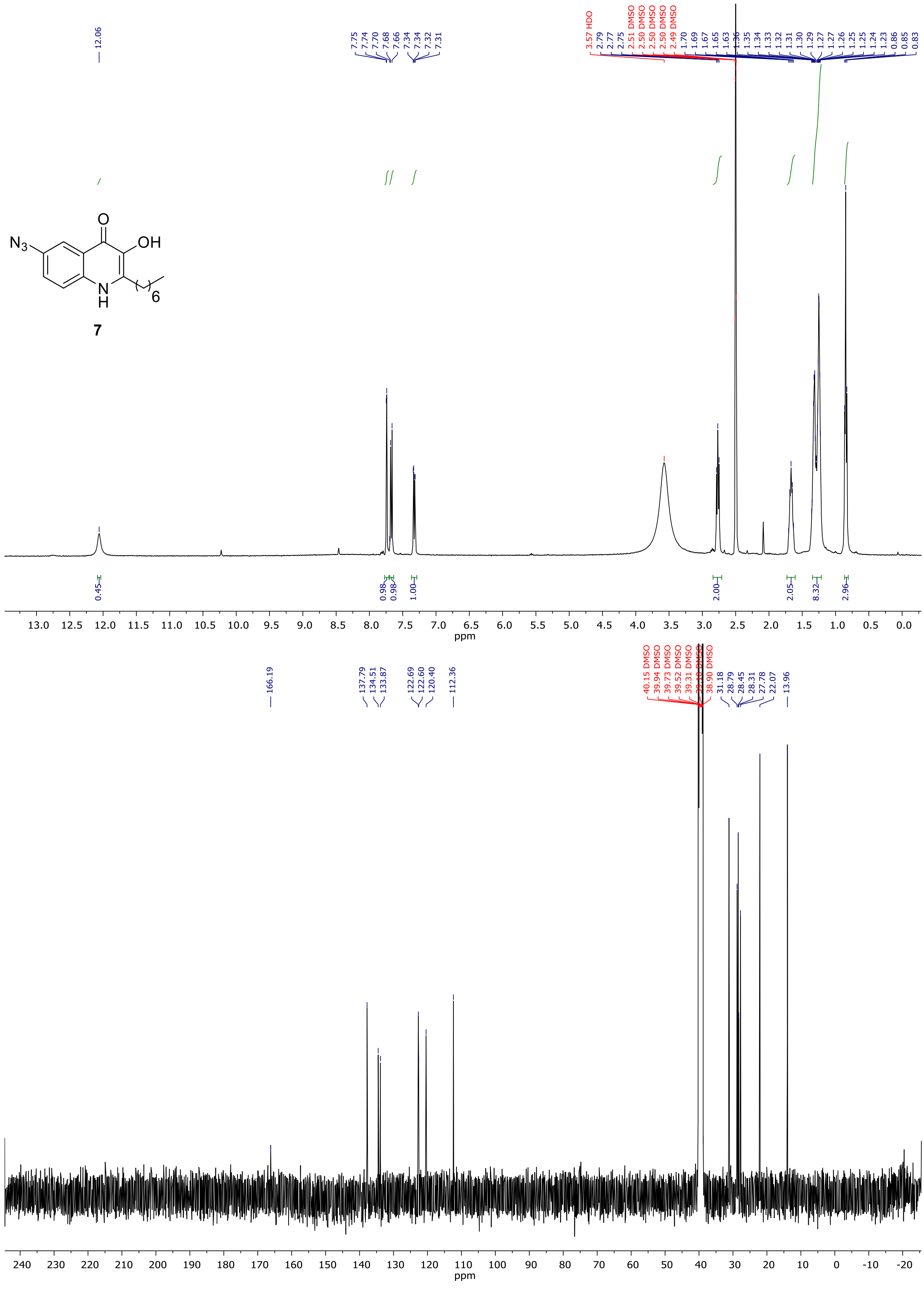

Supplementary Figure 28. ^{1}H and ^{13}C NMR of **7**.

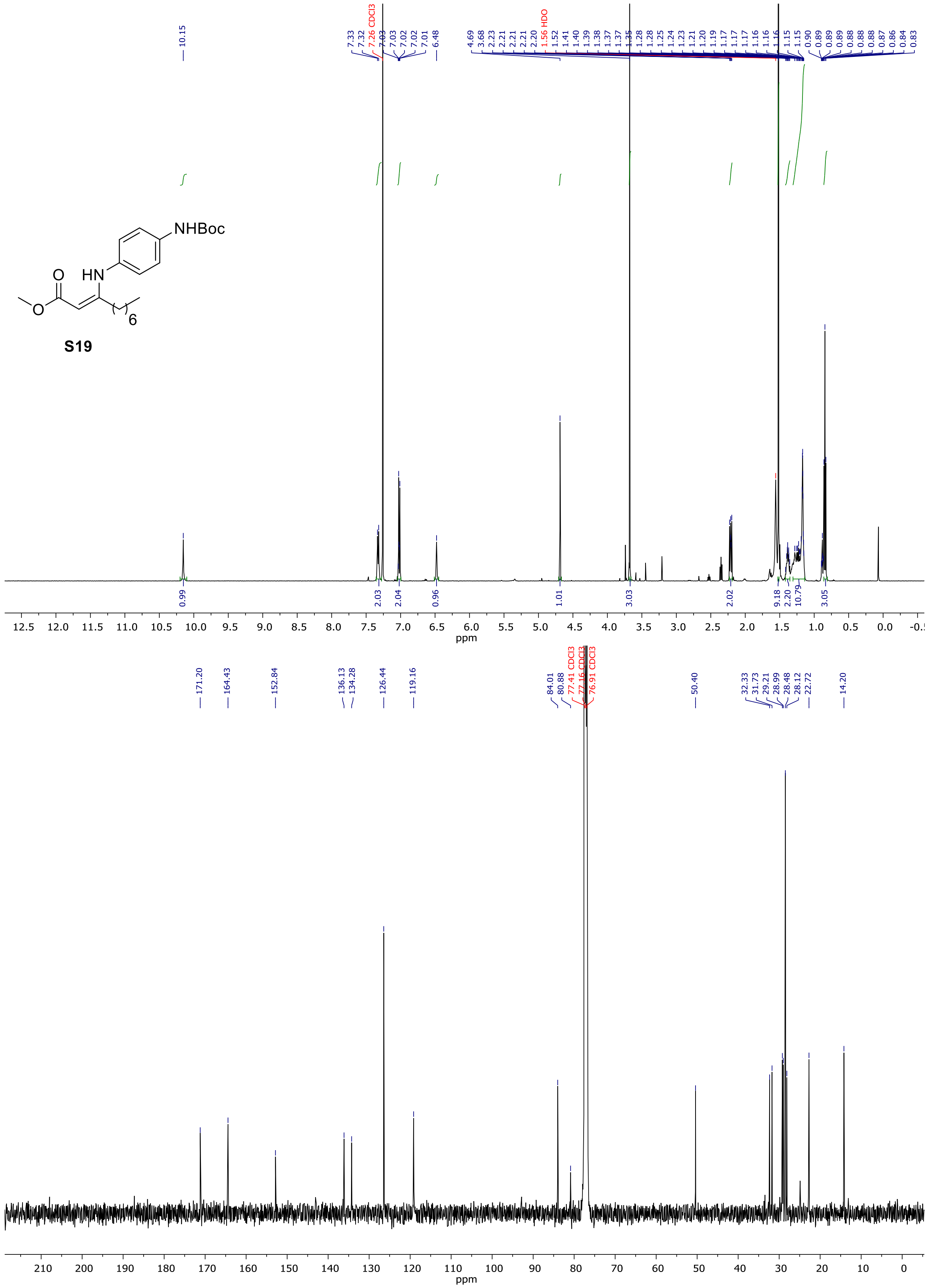

Supplementary Figure 29. ^{1}H and ^{13}C NMR of **S19**.

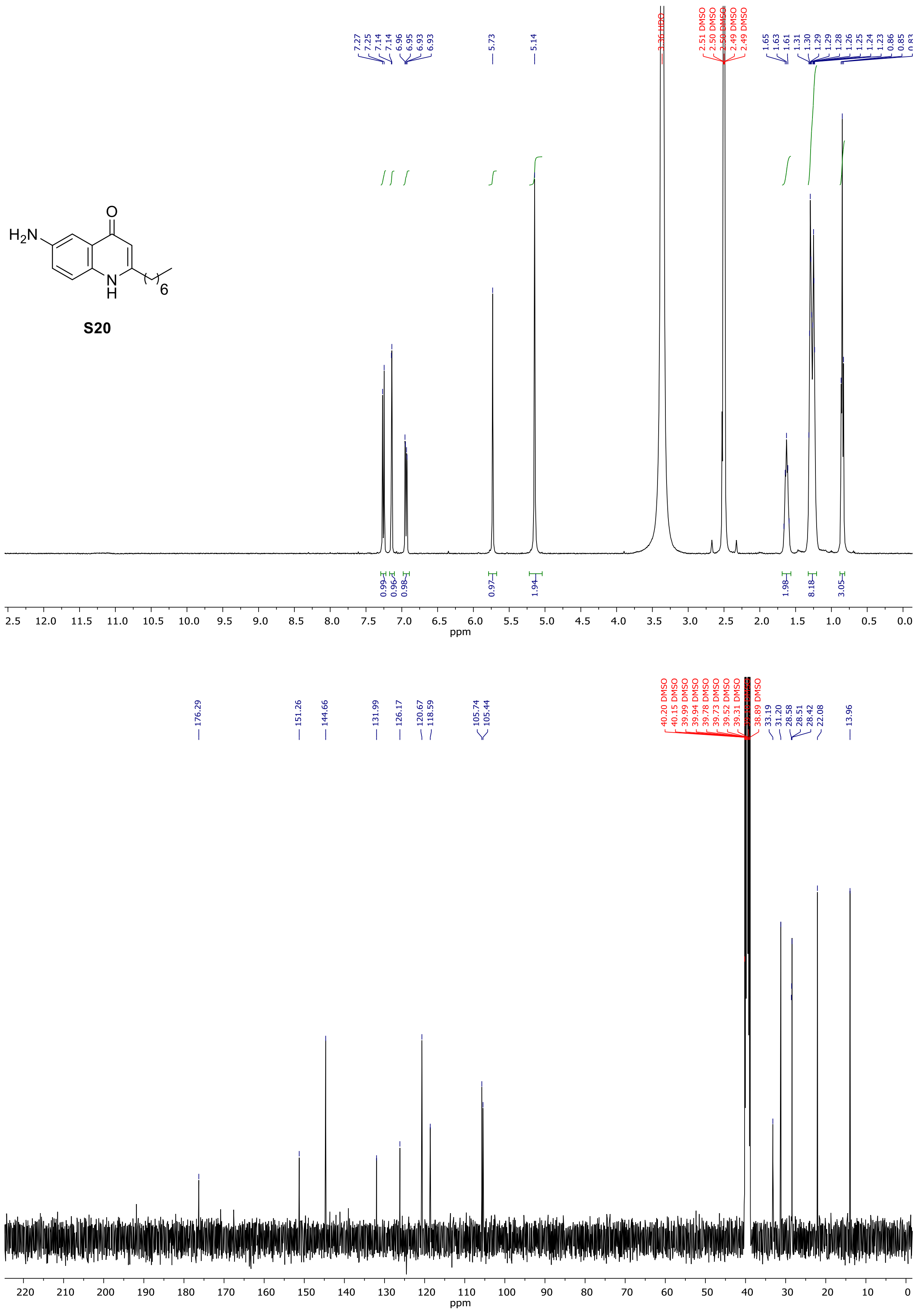

S20

Supplementary Figure 30. ^{1}H and ^{13}C NMR of **S20**.

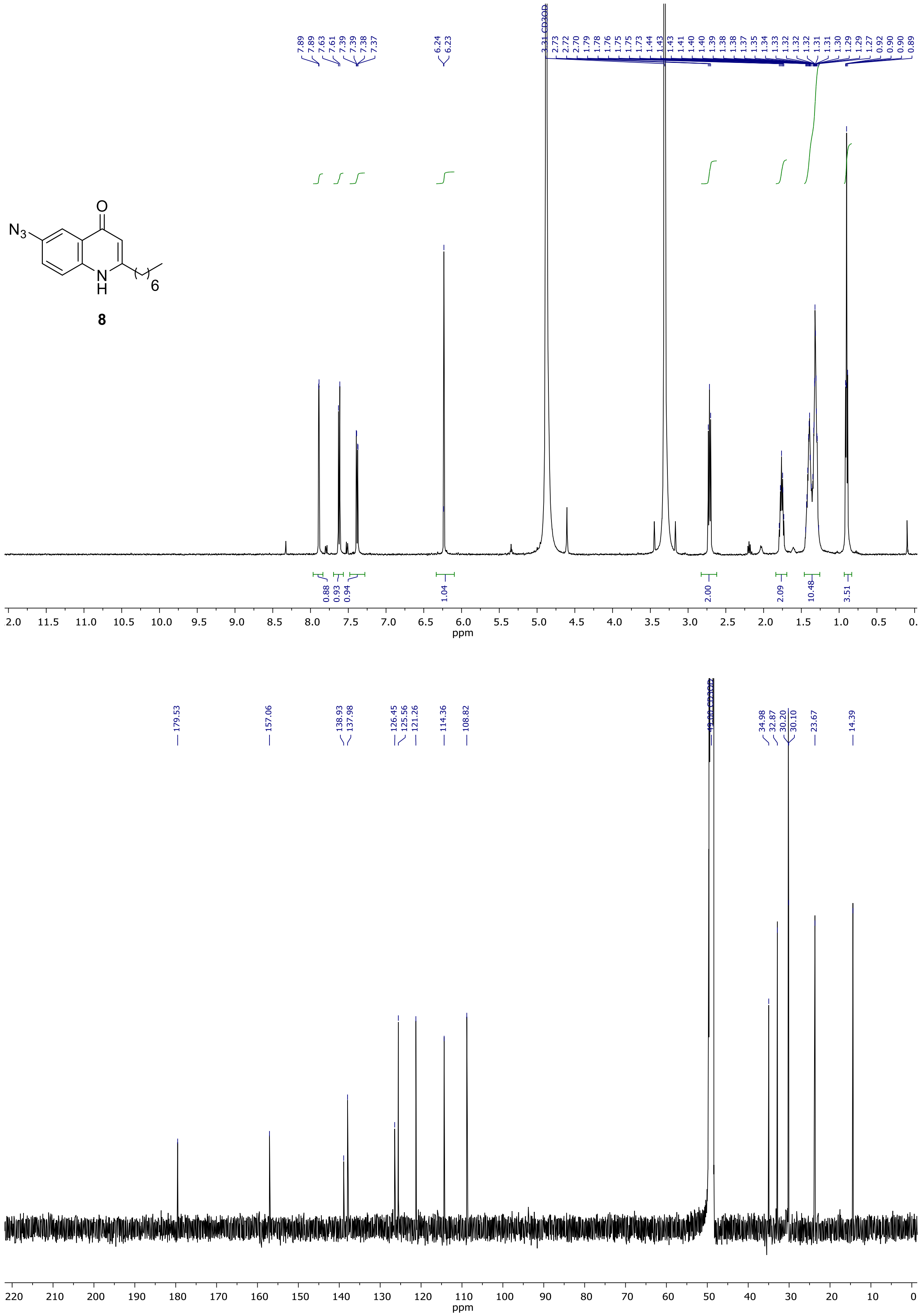

Supplementary Figure 31. ^{1}H and ^{13}C NMR of **8**.

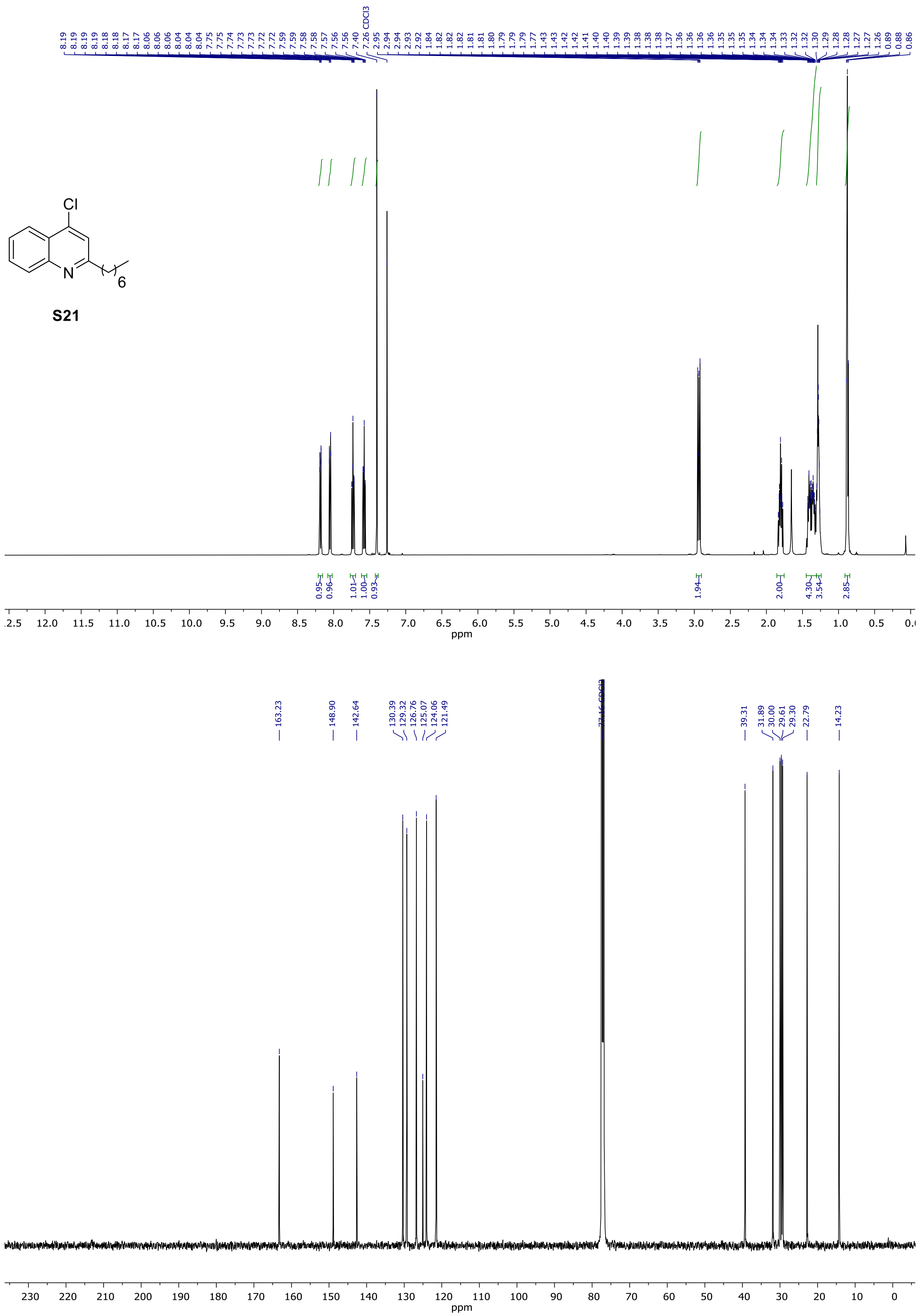

Supplementary Figure 32. ^{1}H and ^{13}C NMR of **S21**.

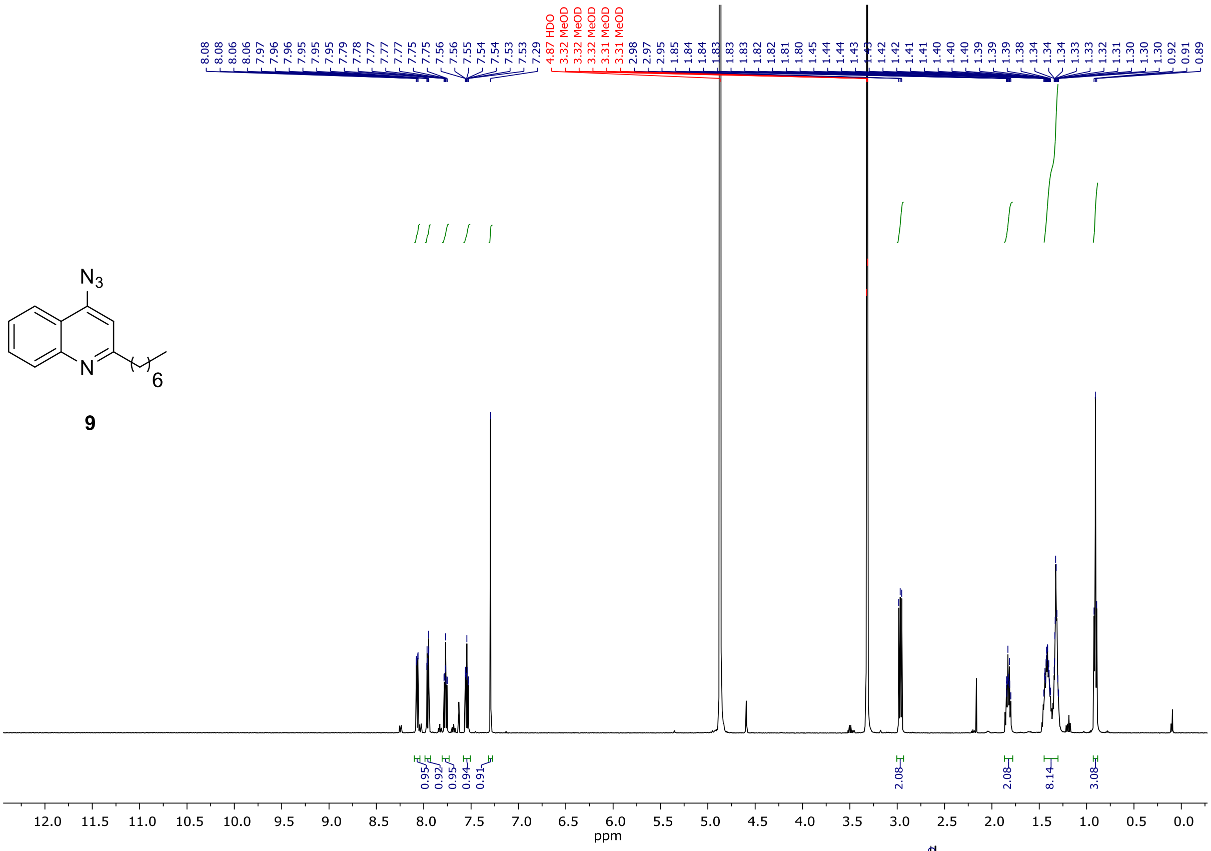

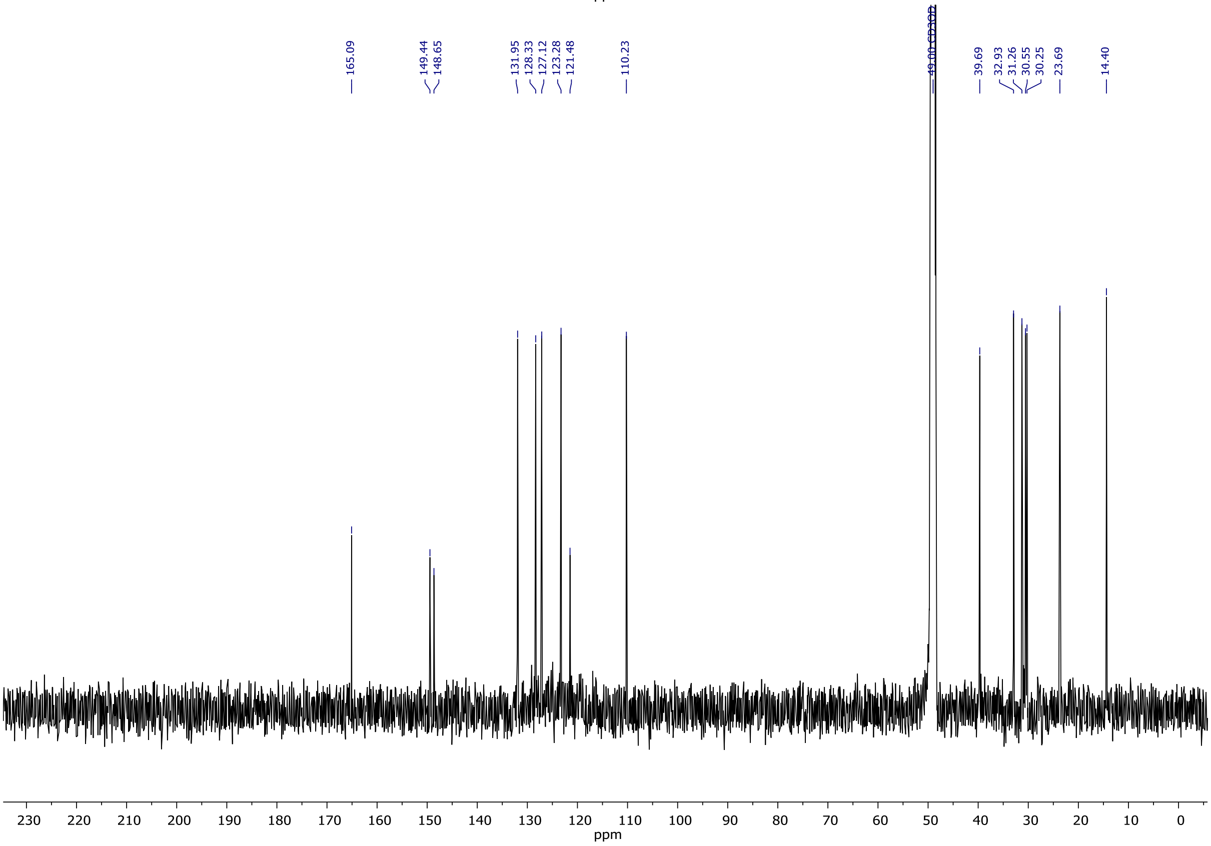

Supplementary Figure 33. ^{1}H and ^{13}C NMR of **9**.

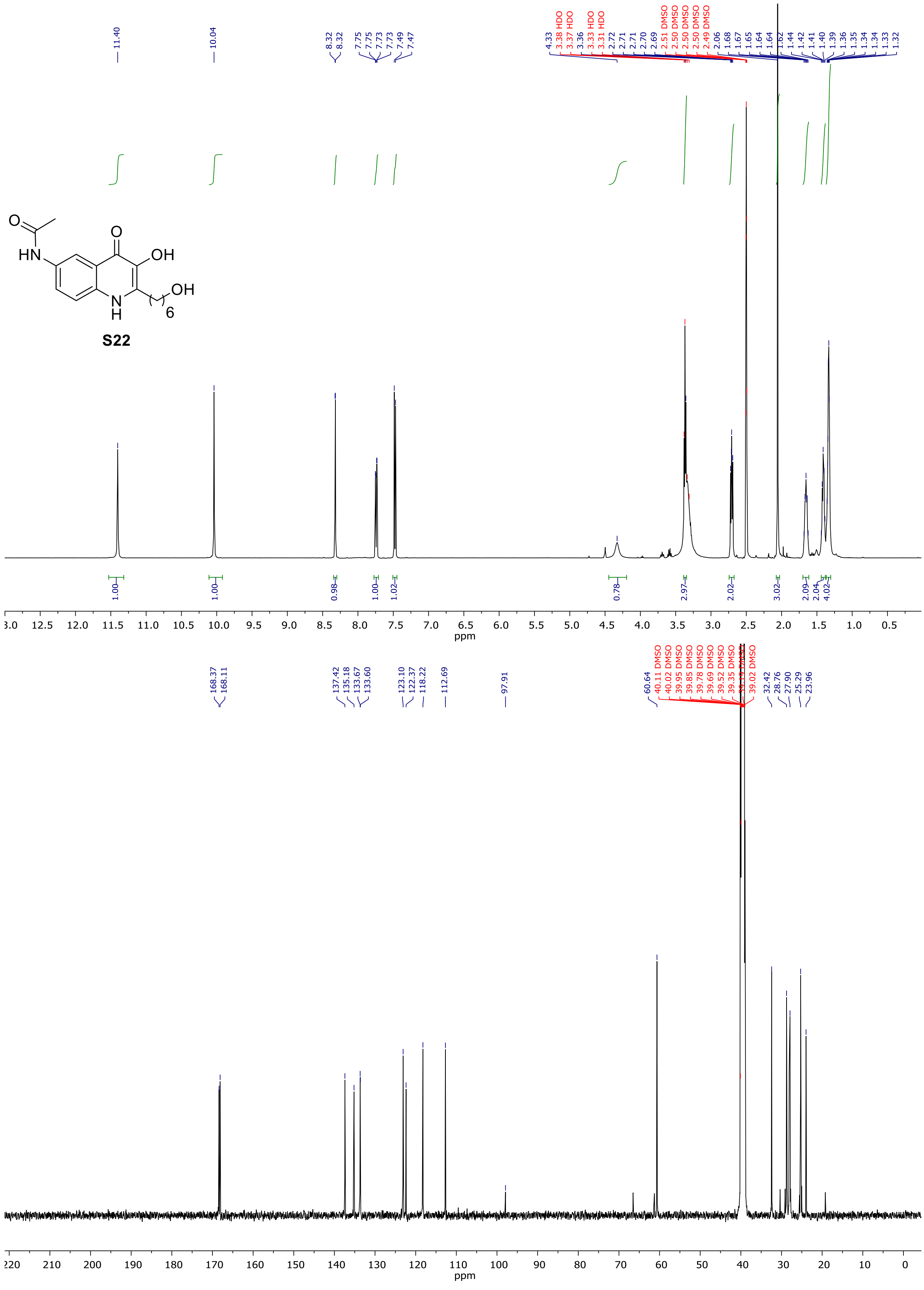

Supplementary Figure 34. ^{1}H and ^{13}C NMR of **S22**.

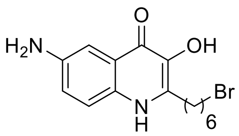

S23

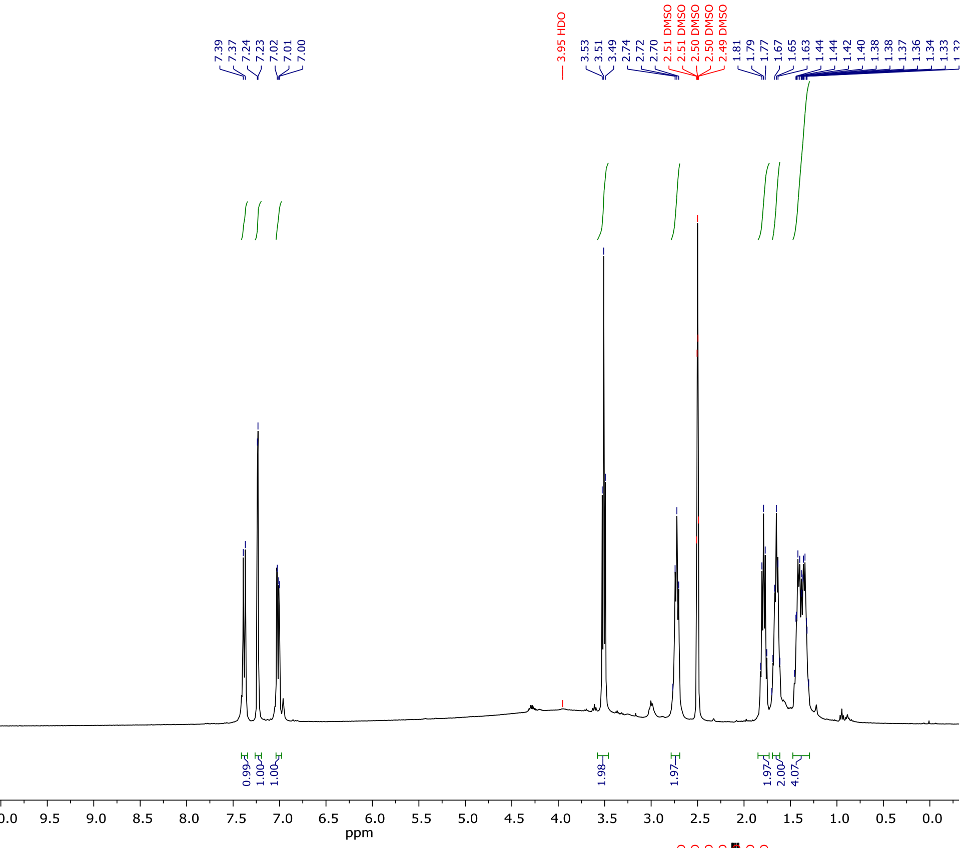

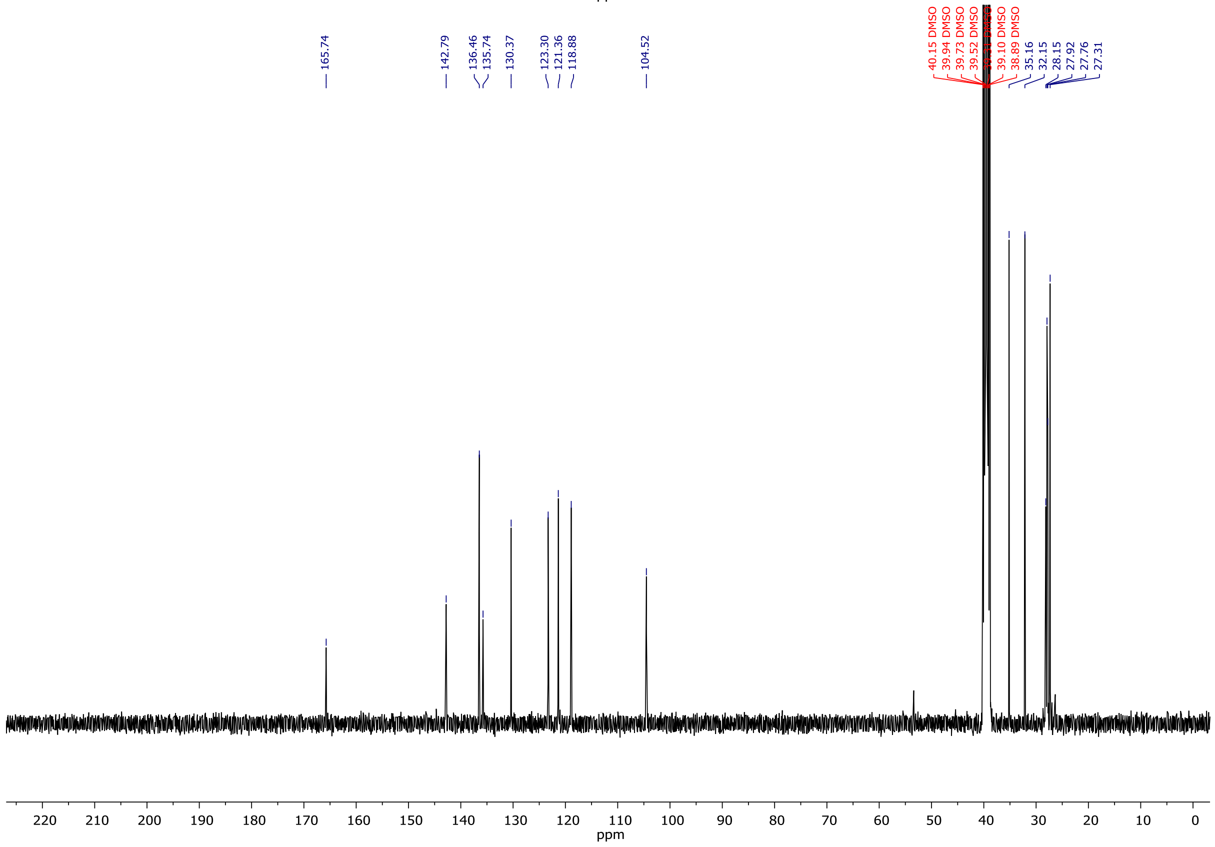

Supplementary Figure 35. ^{1}H and ^{13}C NMR of **S23**.

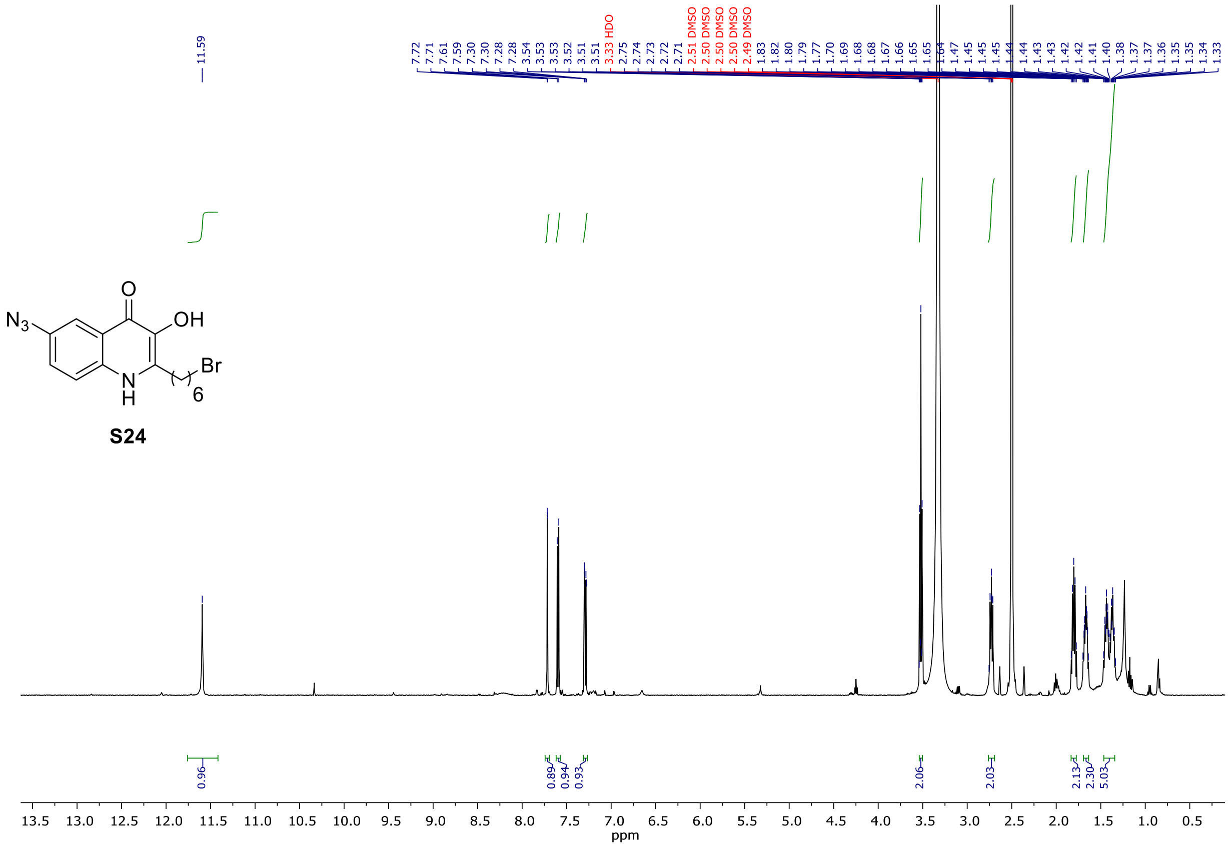

S24

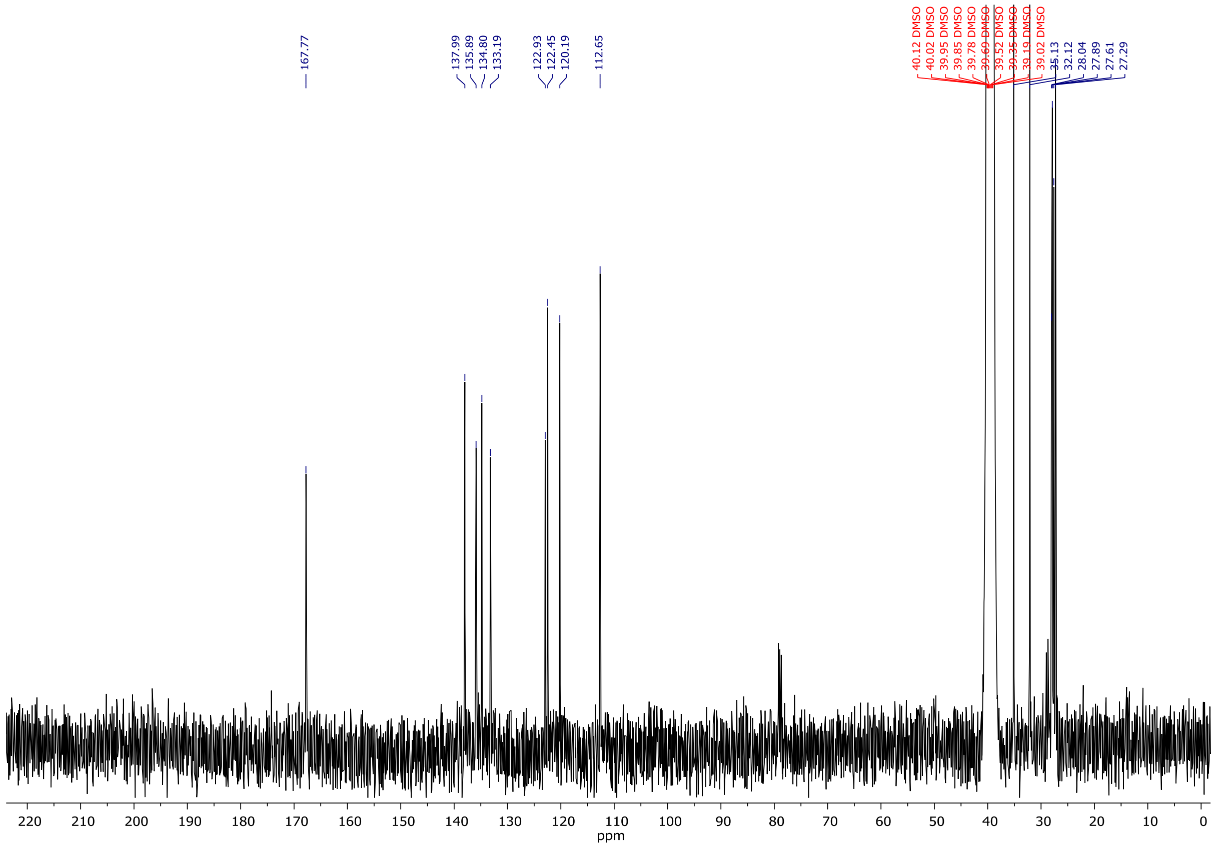

Supplementary Figure 36. ^{1}H and ^{13}C NMR of **S24**.

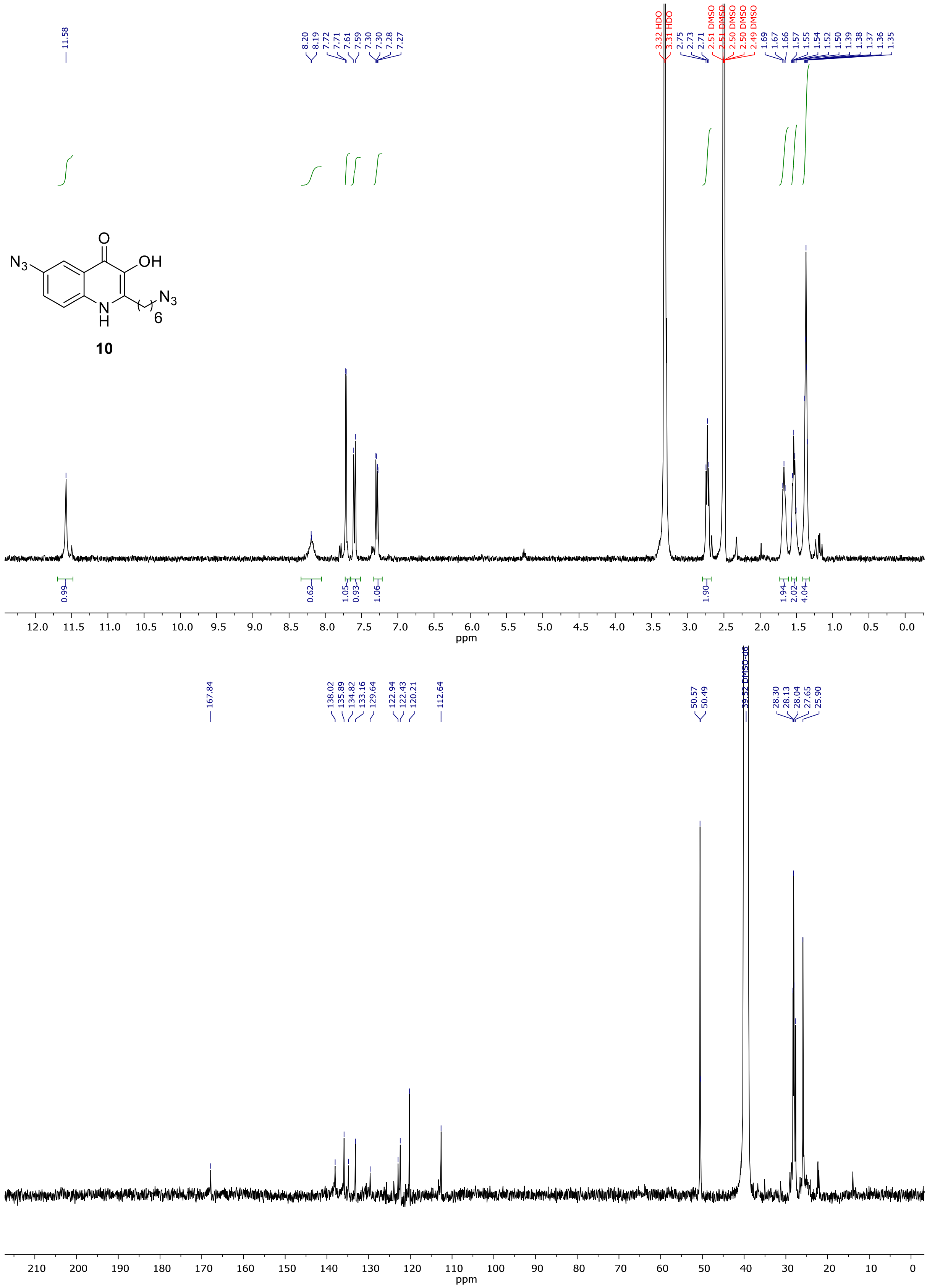

Supplementary Figure 37. ^{1}H and ^{13}C NMR of **10**.

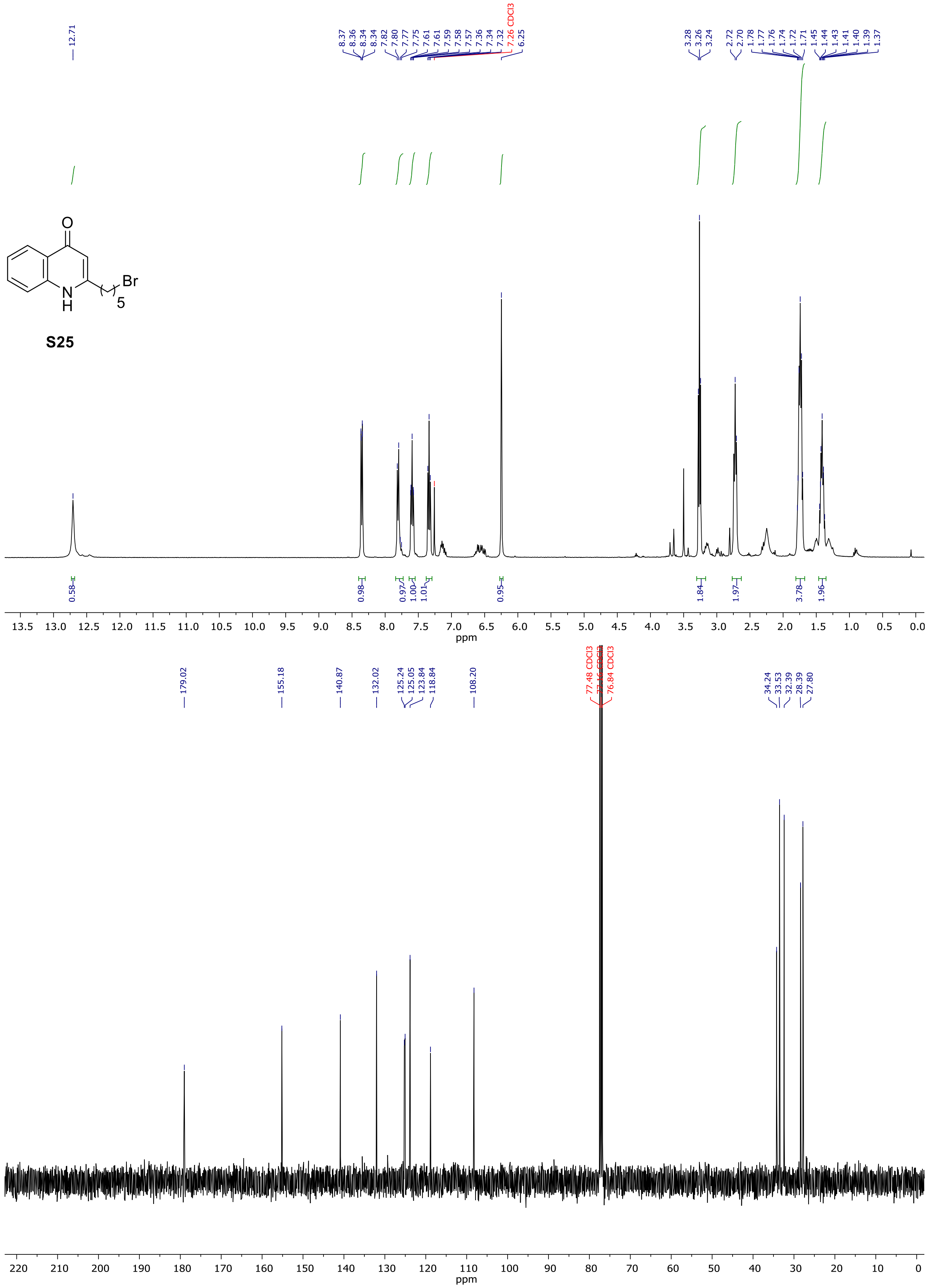

Supplementary Figure 38. ^{1}H and ^{13}C NMR of **S25**.

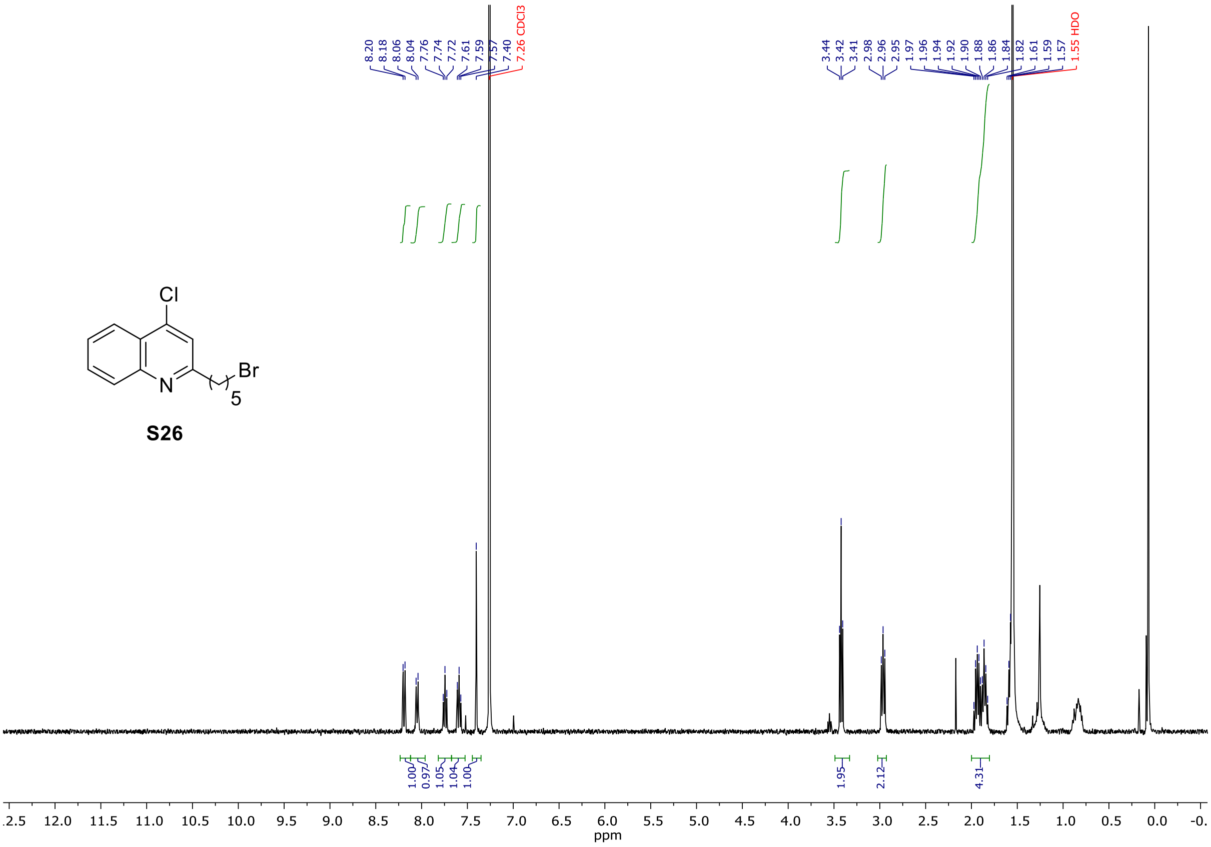

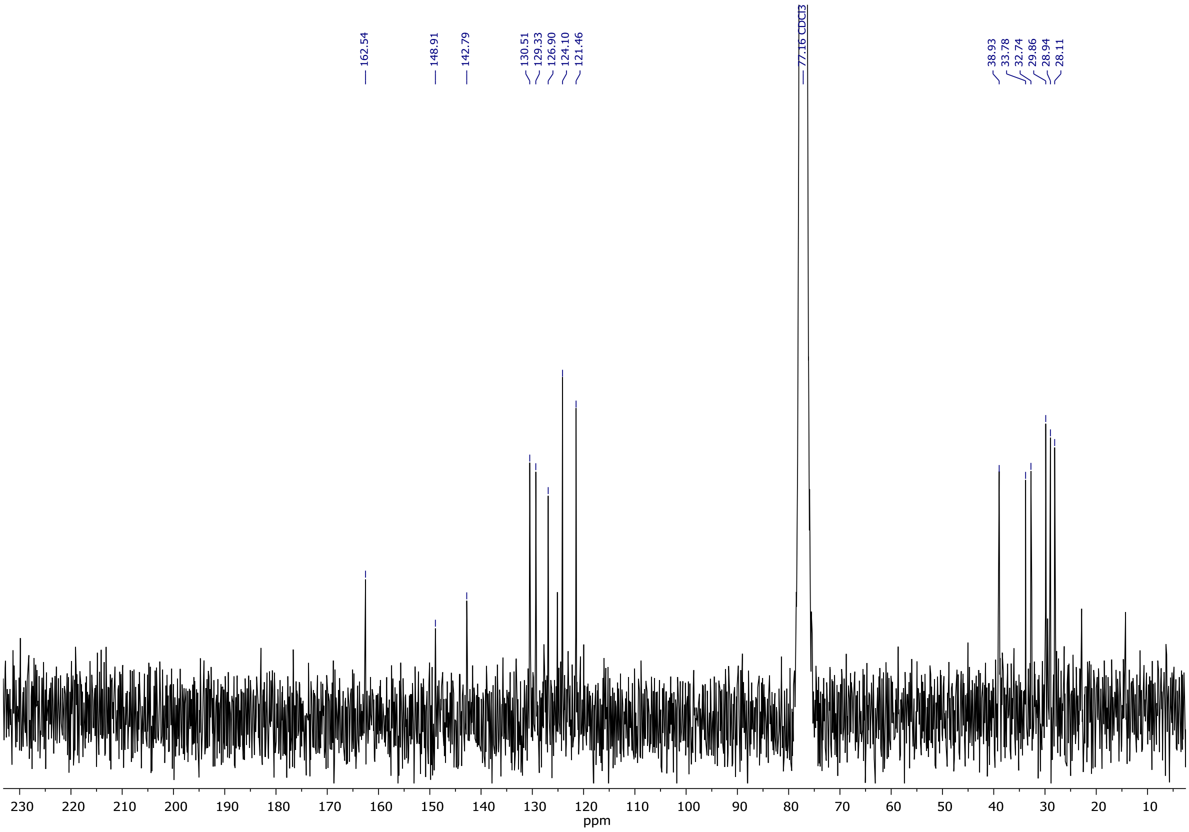

Supplementary Figure 39. ^{1}H and ^{13}C NMR of **S25**.

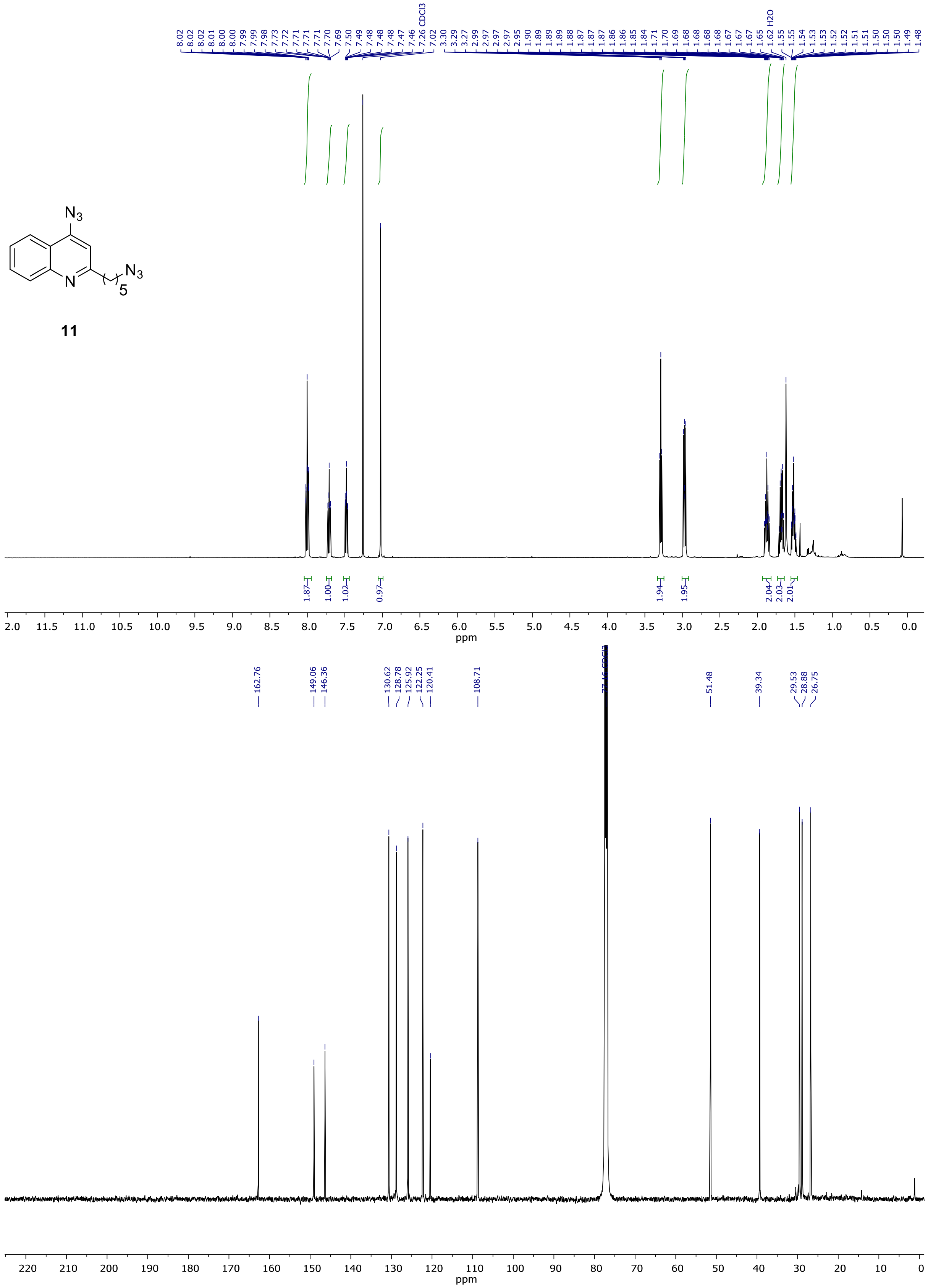

Supplementary Figure 40. ^{1}H and ^{13}C NMR of **11**.

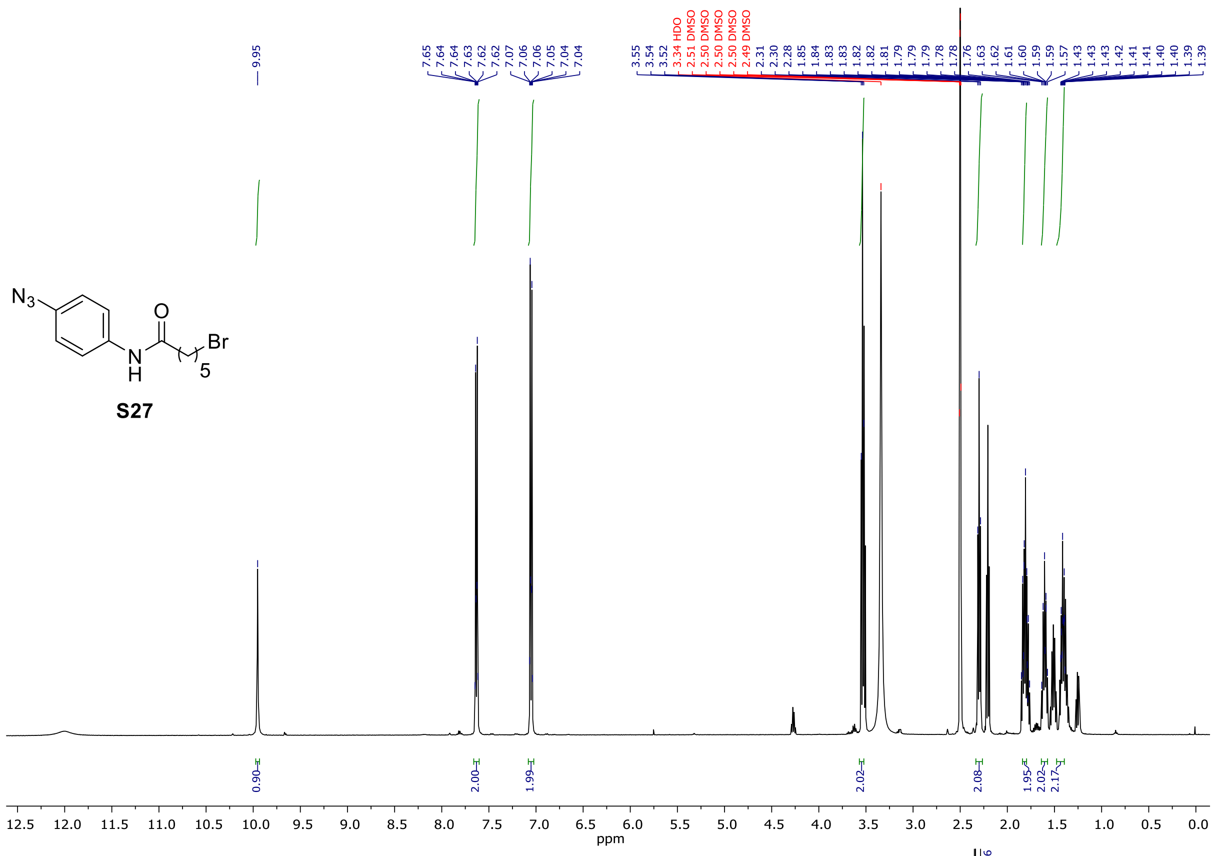

S27

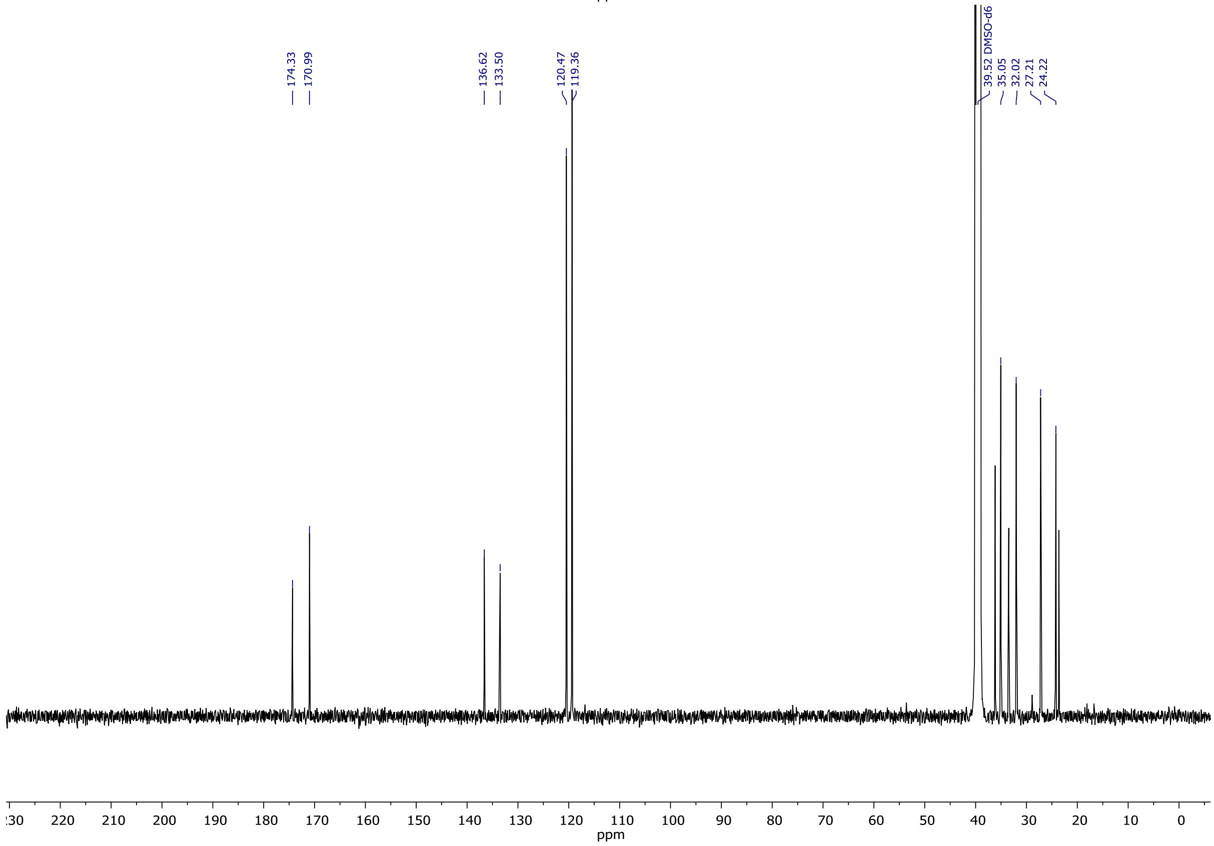

Supplementary Figure 41. ^{1}H and ^{13}C NMR of **S27**.

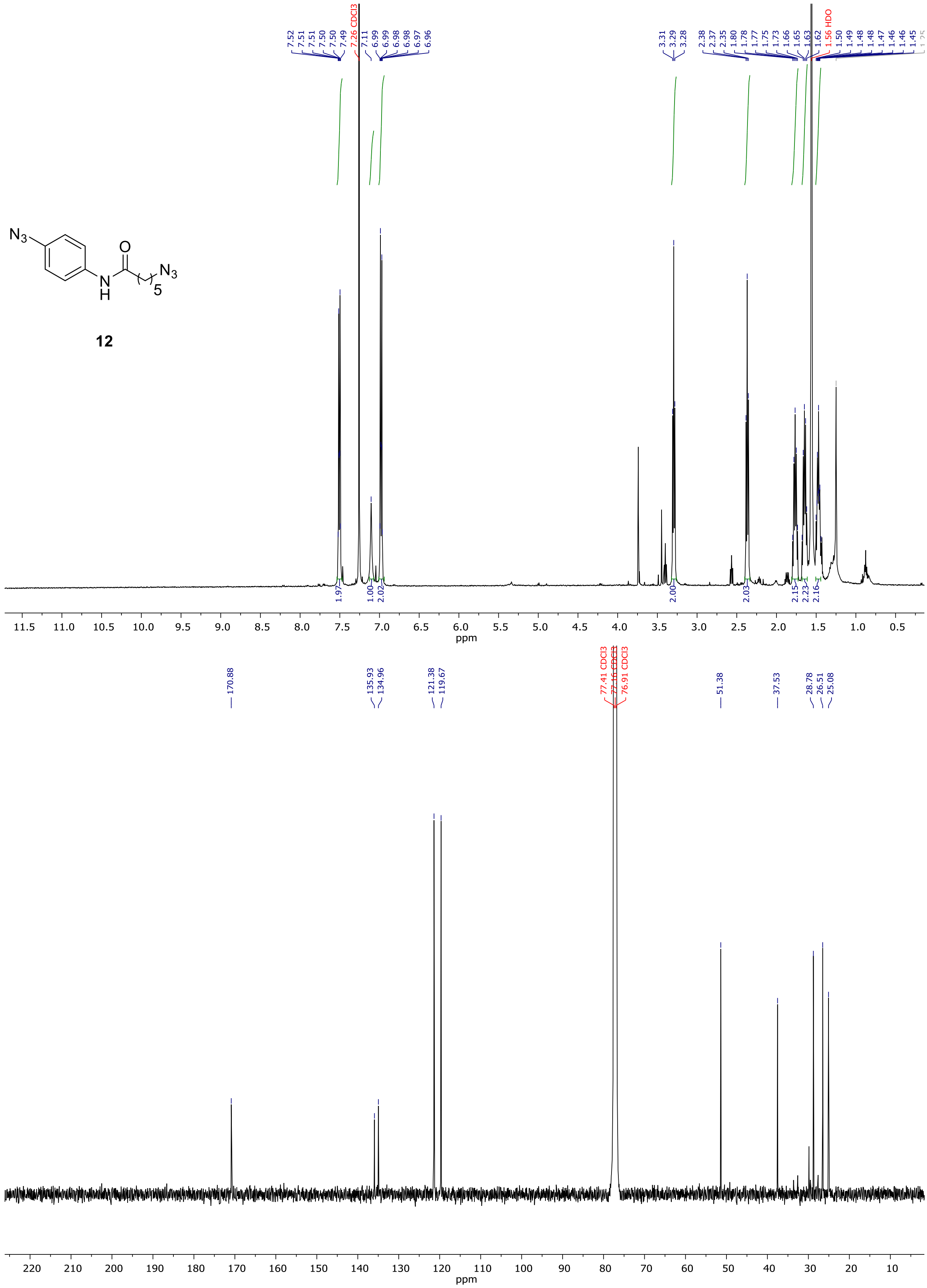

Supplementary Figure 42. ^{1}H and ^{13}C NMR of **12**.

5 Supplementary Appendix 1- UV traces for probes 10-12.

UV trace for probe **10**:

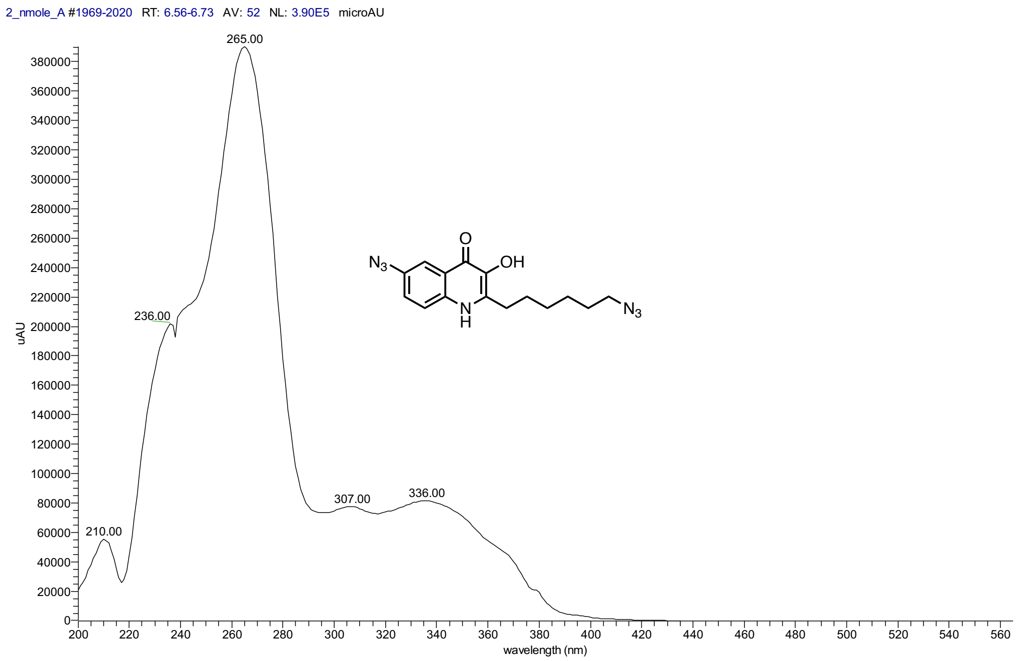

UV trace for probe **11**:

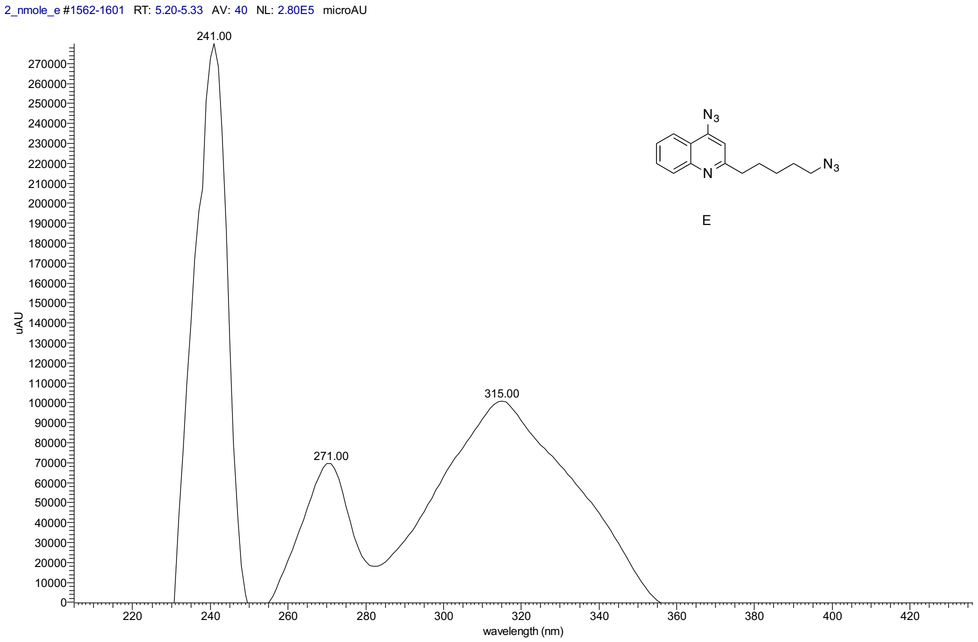

UV trace for probe **12**:

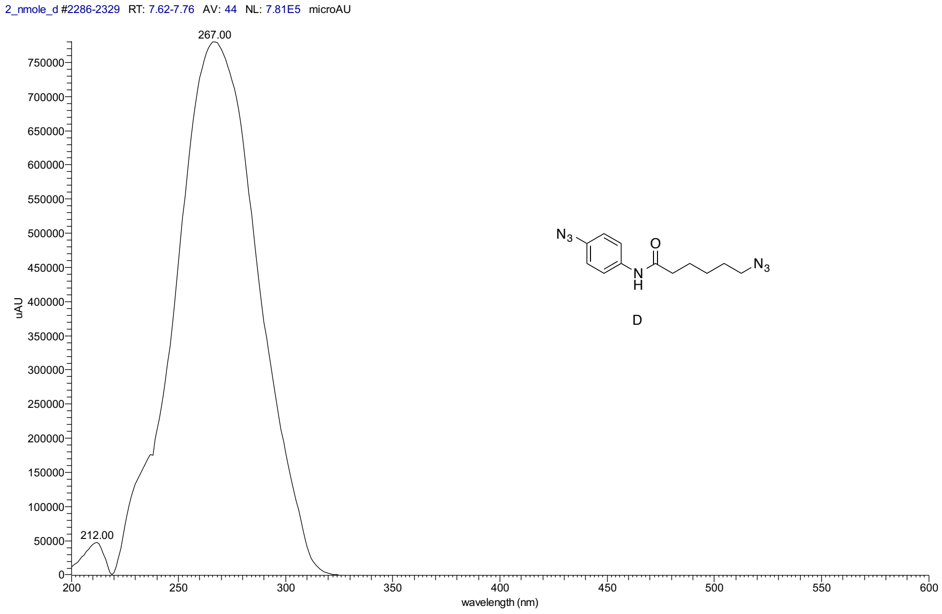

6 Supplementary Appendix 2 - PqsD cloning and purification and fluorimetry protocol

Cloning. The coding region of *pqsD* was amplified by PCR from *P. aeruginosa* genomic DNA using synthetic primers (5'-ATATACTACATATGATGGGTAATCCGATCCTGGC-3' and 5'-ATATGGATCCTCAACATGGCCGGTTCACCT-3') designed according to the published nucleotide sequence of *pqsD*. The PCR product was digested with NdeI and BamHI. The digested PCR product was subsequently cloned into the expression vector, pET-19m.[26] *E. coli* BL21 (DE3) cells containing the plasmid pRARE2 were then transformed with the resulting pET-19m-*pqsD* plasmid.

Protein expression. To overexpress PqsD, overnight cultures of *E. coli* strain BL21 (DE3) carrying pRARE 2 and pET-19m-*pqsD* were inoculated into 2 x 1L of LB containing 50 µg/ml carbenicillin and 25 µg/ml chloramphenicol. Cell cultures were grown at 37ºC with agitation until they reached an absorbance of 0.6 at 600 nm. Isopropyl-β-D-thiogalactopyranoside (IPTG) was added to the cultures at a final concentration of 0.3 mM. The cultures were incubated overnight with agitation at 20ºC. Cells were harvested by centrifugation (11,899 x g, 20 min, 4ºC), and stored at -20ºC for future use. Cell pellets were thawed, and suspended in 10 ml of ice-cold lysis buffer (NaCl 300 mM, 50 mM Tris-HCl pH 8.0, 5% glycerol (v/v), 10 mM imidazole, 5 mM β-mercaptoethanol) containing an EDTA-free protease inhibitor cocktail™ tablet (Roche). Samples were lysed by sonication (5 rounds of 30 seconds at 13 ampere with 1 minute rest on ice between pulses) and clarified by centrifugation (20,000 x g, 30 min, 4ºC). The supernatant was collected and filtered through 0.45 µM membrane filters (Sartorius).

Purification of PqsD. Unless otherwise stated, all purification steps were carried out at 4ºC. The PqsD supernatant was loaded onto a Ni-NTA agarose column (Qiagen) pre-equilibrated with lysis buffer. The column was subsequently washed O/N with approximately 200 ml of lysis buffer, after which PqsD was eluted with elution buffer (NaCl 300 mM, 50 mM Tris-HCl pH 8.0, 5% glycerol (v/v), imidazole 250 mM, 5 mM β-mercaptoethanol). PqsD was concentrated using Sartorius' Vivaspin 2 ultrafiltration column (MWCO: 10,000) and washed several times in dialysis buffer (NaCl 100 mM, 50 mM Tris-HCl pH 8.0, glycerol 5% (v/v)). PqsD was judged pure by SDS-PAGE analysis. The protein was separated into aliquots, flash-frozen in liquid nitrogen, and stored at -80ºC.

Fluorimetry analysis. All fluorescence spectra were generated on a Perkin Elmer LS 55 luminescence spectrometer. PqsD protein (10 µM final concentration) was added to 50 mM Tris-HCl, pH 8.0, 100 mM NaCl, and 10% (v/v) glycerol to a final volume of 1 ml in a quartz, optical cuvette with a 1 cm path length. Spectra illustrated (Fig. 1 below) represent an average of three traces, with no significant variation between each of the three replicates. The excitation wavelength used was 292 nm. At a rate of 10 nm/min, emission spectra were recorded between 300 and 450 nm with the excitation and emission slit widths set at 2.5 nm.

A

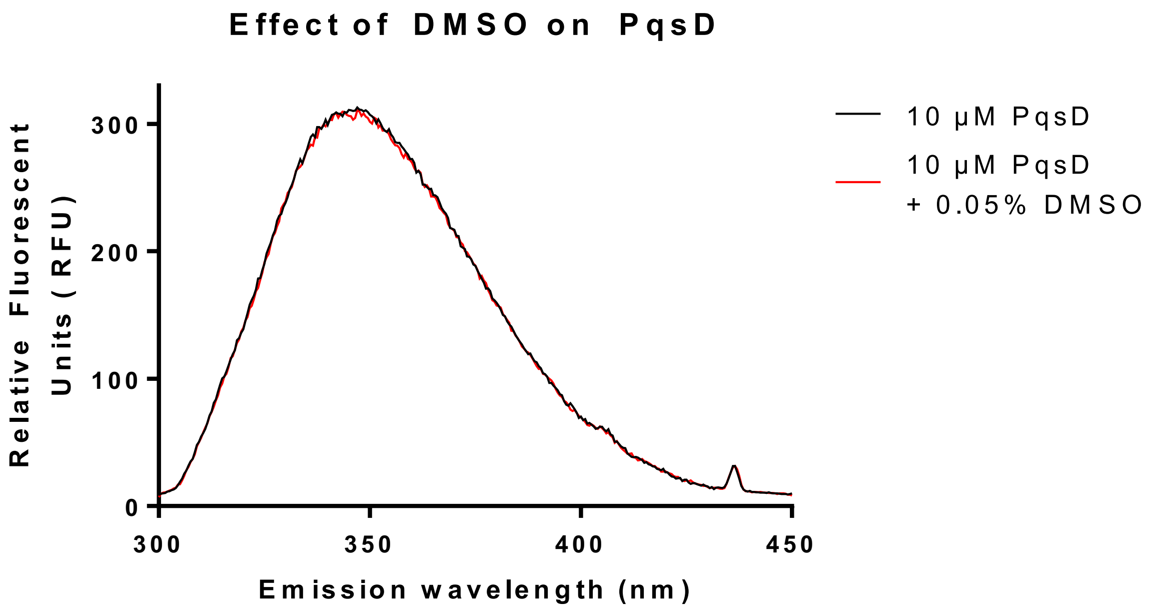

B

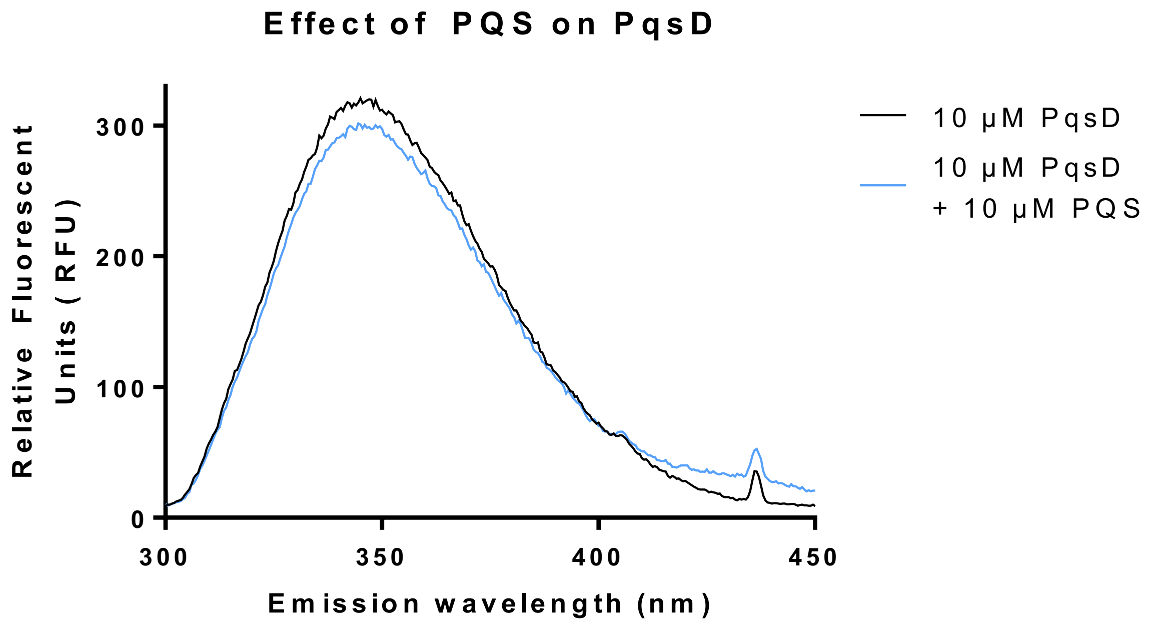

Figure 1. A) 10 μM PqsD with 0.05 % DMSO (vehicle control) and **B)** 10 μM PqsD with 10 μM PQS

7 References

1 M. Welch, J. T. Hodgkinson, J. Gross, D. R. Spring and T. Sams, *Biochemistry*, 2013, **52**, 4433–4438.

2 H. Cao, G. Krishnan, B. Goumnerov, J. Tsongalis, R. Tompkins and L. G. Rahme, *Proc. Natl. Acad. Sci. U. S. A.*, 2001, **98**, 14613–14618.

3 L. Mashburn-Warren, J. Howe, K. Brandenburg and M. Whiteley, *J. Bacteriol.*, 2009, **191**, 3411–3414.

4 E. Cascales, A. Bernadac, M. Gavioli, J.-C. Lazzaroni and R. Lloubes, *J. Bacteriol.*, 2002, **184**, 754–9.

5 A. K. Wessel, J. Liew, T. Kwon, E. M. Marcotte and M. Whiteley, *J. Bacteriol.*, 2013, **195**, 213–219.

6 C. Cugini, M. W. Calfee, J. M. Farrow, D. K. Morales, E. C. Pesci and D. A. Hogan, *Mol. Microbiol.*, 2007, **65**, 896–906.

7 M. A. Welsh, N. R. Eibergen, J. D. Moore and H. E. Blackwell, *J. Am. Chem. Soc.*, 2015, **137**, 1510–1519.

8 M. J. Gambello, S. Kaye and B. H. Iglewski, *Infect. Immun.*, 1993, **61**, 1180–1184.

9 J. M. Farrow, Z. M. Sund, M. L. Ellison, D. S. Wade, J. P. Coleman and E. C. Pesci, *J. Bacteriol.*, 2008, **190**, 7043–51.

10 L. A. Gallagher, S. L. McKnight, M. S. Kuznetsova, E. C. Pesci and C. Manoil, *J. Bacteriol.*, 2002, **184**, 6472–6480.

11 S. A. Beatson, C. B. Whitchurch, A. B. T. Semmler and J. S. Mattick, *J. Bacteriol.*, 2002, **184**, 3598–3604.

12 S. P. Diggle, S. Matthijs, V. J. Wright, M. P. Fletcher, S. R. Chhabra, I. L. Lamont, X. Kong, R. C. Hider, P. Cornelis, M. Cámara and P. Williams, *Chem. Biol.*, 2007, **14**, 87–96.

13 D. Wessel and U. I. Flügge, *Anal. Biochem.*, 1984, **138**, 141–143.

14 N. Li, C.-L. Kuo, G. Paniagua, H. van den Elst, M. Verdoes, L. I. Willems, W. A. van der Linden, M. Ruben, E. van Genderen, J. Gubbens, G. P. van Wezel, H. S. Overkleeft and B. I. Florea, *Nat. Protoc.*, 2013, **8**, 1155–1168.

15 H. E. Gottlieb, V. Kotlyar and A. Nudelman, *J. Org. Chem.*, 1997, **62**, 7512–7515.

16 J. T. Hodgkinson, W. R. J. D. Galloway, M. Welch and D. R. Spring, *Nat. Protoc.*, 2012, **7**, 1184–1192.

17 A. Woschek, M. Mahout, K. Mereiter and F. Hammerschmidt, *Synthesis*, **2007**, 1517–1522.

18 M. Gómez-García, J. M. Benito, R. Gutiérrez-Gallego, A. Maestre, C. O. Mellet, J. M. G. Fernández and J. L. J. Blanco, *Org. Biomol. Chem.*, 2010, **8**, 1849.

19 F. Jahani, M. Tajbakhsh, H. Golchoubian and S. Khaksar, *Tetrahedron Lett.*, 2011, **52**, 1260–1264.

20 C. Mugnaini, C. Falciani, M. De Rosa, A. Brizzi, S. Pasquini and F. Corelli, *Tetrahedron*, 2011, **67**, 5776–5783.

21 J. T. Hodgkinson, J. Gross, Y. R. Baker, D. R. Spring and M. Welch, *Chem. Sci.*, 2016, **7**, 2553–2562.

22 N. Hashimoto, T. Funatomi, T. Misaki and Y. Tanabe, *Tetrahedron*, 2006, **62**, 2214–2223.

23 W. Wang, B. Xu and G. B. Hammond, *J. Org. Chem.*, 2009, **74**, 1640–1643.

24 Y. Oikawa, K. Sugano and O. Yonemitsu, *J. Org. Chem.*, 1978, **43**, 2087–2088.

25 A. L. Garner and K. D. Janda, *Angew. Chemie Int. Ed.*, 2010, **49**, 9630–9634.

26 S. K. Dolan, T. Bock, V. Hering, R. A. Owens, G. W. Jones, W. Blankenfeldt and S. Doyle, *Open Biol.*, 2017, DOI: 10.1098/rsob.160292.
